# Structural mechanism for noncanonical GPCR signaling in the Hedgehog pathway

**DOI:** 10.1038/s41594-026-01800-z

**Published:** 2026-04-30

**Authors:** William P. Steiner, Nathan Iverson, Guibing Liu, Varun Venkatakrishnan, Jian Wu, Tomasz Maciej Stepniewski, Zachary Michaelson, Jan W. Bröckel, Ju-Fen Zhu, Jessica G. H. Bruystens, Annabel Lee, Isaac Nelson, Daniela Bertinetti, Corvin D. Arveseth, Gerald Tan, Paul Spaltenstein, Jiewei Xu, Ruth Hüttenhain, Michael S. Kay, Friedrich W. Herberg, Erhu Cao, Jana Selent, Ganesh S. Anand, Roland L. Dunbrack, Susan S. Taylor, Benjamin R. Myers

**Affiliations:** 1 Department of Oncological Sciences, Huntsman Cancer Institute, University of Utah School of Medicine, Salt Lake City, UT USA; 2 Department of Biochemistry, University of Utah School of Medicine, Salt Lake City, UT USA; 3 Department of Bioengineering, University of Utah, Salt Lake City, UT USA; 4 Department of Chemistry, Pennsylvania State University, University Park, PA USA; 5 Department of Pharmacology, University of California, San Diego, La Jolla, CA USA; 6 Research Programme on Biomedical Informatics (GRIB), Hospital del Mar Medical Research Institute (IMIM) and Pompeu Fabra University (UPF), Barcelona, Spain; 7 InterAx Biotech AG, Villigen, Switzerland; 8 Department of Biochemistry, Institute for Biology, University of Kassel, Kassel, Germany; 9 Department of Cellular and Molecular Pharmacology, University of California, San Francisco, San Francisco, CA USA; 10 Department of Molecular and Cellular Physiology, Stanford University School of Medicine, Stanford, CA USA; 11 The Huck Institutes of the Life Sciences, Pennsylvania State University, University Park, PA USA; 12 Institute for Cancer Research, Fox Chase Cancer Center, Philadelphia, PA USA; 13 Department of Biochemistry and Molecular Biophysics, University of California, San Diego, La Jolla, CA USA; 14 Present Address: Idaho College of Osteopathic Medicine, Meridian, ID USA; 15 Present Address: Program in Cell and Molecular Biology, Duke University School of Medicine, Durham, NC USA; 16 Present Address: Medical Scientist Training Program, Washington University School of Medicine, St. Louis, MO USA

**Keywords:** G protein-coupled receptors, Kinases, Molecular modelling, Cell signalling, Intrinsically disordered proteins

## Abstract

The Hedgehog (Hh) pathway is fundamental to embryogenesis, tissue homeostasis and cancer. Hh signals are transduced through an unusual mechanism; upon agonist-induced phosphorylation, the noncanonical G-protein-coupled receptor (GPCR) Smoothened (SMO) binds the protein kinase A (PKA) catalytic subunit (PKA-C) and physically blocks its enzymatic activity. Here, by combining computational structural approaches with biochemical and functional studies, we show that SMO mimics strategies prevalent in canonical GPCR and PKA signaling complexes, despite little sequence or secondary-structure homology. The intrinsically disordered SMO cytoplasmic domain binds the PKA-C active site, resembling the regulatory subunit within PKA holoenzymes, while the SMO transmembrane domain binds a conserved PKA-C interaction hub. Unlike prevailing GPCR signal transduction models, phosphorylation of SMO promotes intramolecular electrostatic interactions that stabilize structural elements within its cytoplasmic domain, thereby remodeling it into a PKA-inhibiting conformation. Our work provides a structural mechanism for a central step in Hh signaling and defines a principle for disordered GPCR domains to transmit signals intracellularly.

## Abstract (web summary)

The authors performed computational and experimental analyses to reveal how Smoothened directly inhibits PKA through an intrinsically disordered region, defining a central step in Hedgehog signaling and a mechanism of G-protein-coupled receptor–kinase regulation.

## Main

Communication between G-protein-coupled receptors (GPCRs) and protein kinase A (PKA) is fundamental to human physiology and malfunctions in GPCR–PKA communication can lead to disease^1,2^. Canonical GPCRs regulate PKA by coupling to heterotrimeric G proteins that control cyclic AMP (cAMP), a second messenger that unleashes PKA catalytic (PKA-C) subunits from inhibition by PKA regulatory (PKA-R) subunits in PKA holoenzymes^1,2^. In contrast, during vertebrate Hedgehog (Hh) signal transduction, the noncanonical class F GPCR Smoothened (SMO) inhibits PKA independently of classical G protein and cAMP-dependent cascades^3–5^. Instead, activation of SMO by sterol agonists^6–12^ triggers insertion of a PKA pseudosubstrate motif in SMO’s intracellular membrane-proximal C terminus (pCT) into the PKA-C active site^13–15^. This binding interrupts PKA-C’s catalytic cycle^13–15^, relieving glioma-associated (GLI) transcription factors from phosphorylation-induced inhibition and enabling Hh pathway target gene expression^13–15^. These events occur within the primary cilium, a small cell-surface compartment believed to promote key Hh pathway biochemical reactions because of its high surface-area-to-volume ratio, compartmentalized architecture and distinctive protein and lipid composition^16–18^.

**Fig. 1:**
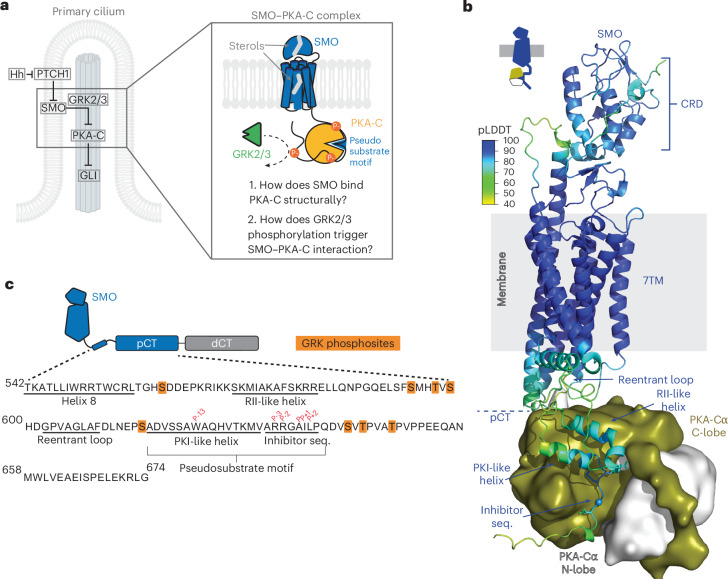
AlphaFold modeling of the SMO–PKA-C complex. **a**, Left, schematic diagram of Hh pathway regulation in the primary cilium, showing key components and their functional relationships. Right, central questions investigated in this study are highlighted (details in the text). **b**, AlphaFold3 model of phosphorylated mouse SMO in complex with mouse PKA-Cα (rendered as a surface with small N-lobe in white and large C-lobe in olive). Positions of the SMO extracellular CRD, 7TM domain and pCT are indicated and the approximate location of the membrane is shown in gray. The PKI-like helix, inhibitor sequence, RII-like helix and reentrant loop are labeled (details in the text). Top left, the AlphaFold3 pLDDT score (0–100, with higher values indicating greater confidence) is represented by a color scale. The turquoise sphere in SMO’s inhibitor sequence represents the P-site. **c**, Sequence of SMO helix 8 and pCT. Secondary-structure elements are underlined, GRK2/3 phosphorylation sites are highlighted in orange and key amino acids in the pseudosubstrate motif are labeled in red.

SMO–PKA-C interactions are regulated such that only the active SMO conformation inhibits PKA-C, ensuring proper control of GLI transcription and preventing pathological outcomes^13,15^. Thus, GPCR kinases 2 and 3 (GRK2/3) recognize active, sterol-bound SMO and phosphorylate the pCT, thereby enhancing interactions between the SMO pseudosubstrate motif and PKA-C^13,15^. Although phosphorylated GPCRs often bind cytoplasmic signaling proteins indirectly through β-arrestin scaffolds^19–22^, in vitro reconstitution studies established that GRK2/3 phosphorylation of SMO triggers a direct SMO–PKA-C interaction^15^. The SMO–GRK2/3–PKA pathway is essential to Hh signal transduction^13–15,23–26^, although other processes contribute (Discussion); however, how SMO binds PKA-C in structural terms and how GRK2/3 phosphorylation facilitates this process are unknown (Fig. 1a).

Numerous structural studies have explored how canonical GPCRs bind conventional downstream effectors^19–21,27^, as well as how PKA-C engages classical pseudosubstrates^2,28–30^. The SMO–PKA-C interaction differs from these signaling complexes in two fundamental ways. First, PKA-C is unique among GPCR effectors; it is unrelated to G proteins or β-arrestins and lacks obvious GPCR-interacting domains^19,20^. Second, unlike conventional PKA inhibitors, such as the heat-stable protein kinase inhibitor (PKI) proteins or PKA-R subunits, which interact with PKA-C constitutively or under cAMP-depleted conditions, respectively^28,31^, SMO requires GRK2/3 phosphorylation to bind and inhibit PKA-C^13–15^. Consistent with this unprecedented mode of PKA regulation, SMO displays homology to PKI proteins and PKA-R subunits only within its pseudosubstrate region^14^ and lacks the additional structural elements needed in these proteins for efficient PKA-C binding^32–35^. Thus, existing GPCR and PKA structures offer little insight into the SMO–PKA-C complex.

The intracellular segments (CT and intracellular loop 3 (ICL3)) of both canonical and noncanonical GPCRs (including SMO) are predicted to be intrinsically disordered regions (IDRs)^36^. IDRs adopt a continuum of conformational states^37–40^, presenting a formidable challenge for structural characterization. Indeed, these GPCR IDRs—critical for signaling—are largely unresolved in or absent from most GPCR crystallography or cryo-electron microscopy (cryo-EM) structures^36,41,42^, including the >20 SMO structures solved to date^7,11,43–46^. To overcome these limitations, we analyzed the SMO–PKA-C complex by integrating computational structure prediction and molecular dynamics (MD) simulations with biochemical, biophysical and functional studies. The resulting structural model reveals key mechanistic insights into this previously inaccessible signaling complex.

## Results

### AlphaFold modeling of the SMO–PKA-C complex

**Extended Data Fig. 1:**
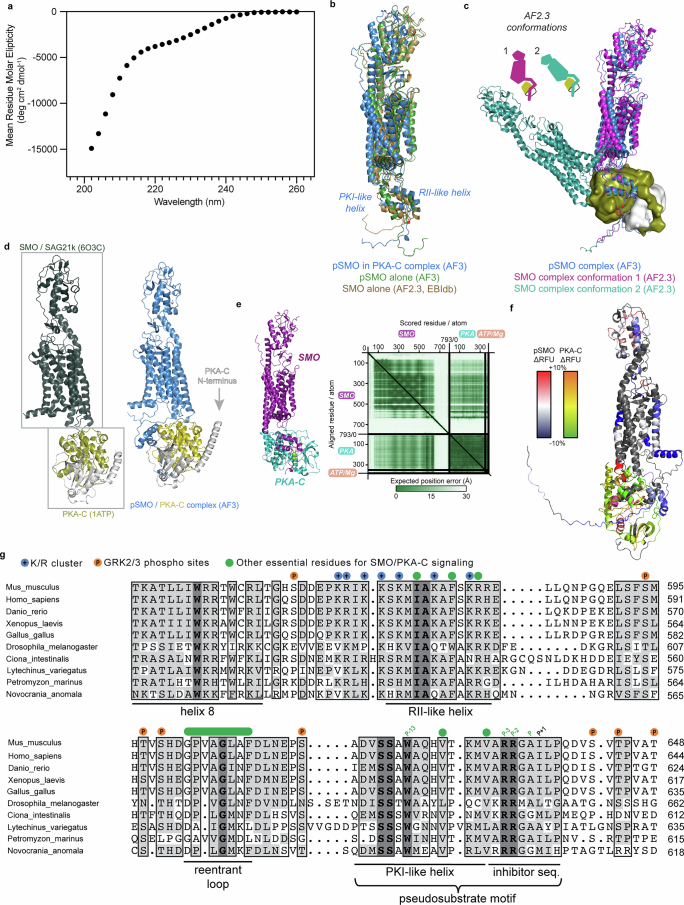
SMO binds PKA-C via its intrinsically disordered C-terminus. **a**, CD analysis of the purified SMO pCT, spanning residues 565–657 (40 μM). **b**, AlphaFold 3 models of SMO from the phosphorylated SMO / PKA-C complex (blue) aligned with the AlphaFold 3 model of SMO alone, with phosphorylation sites included (green), or the AlphaFold 2.3.0 model of SMO alone, downloaded from the EBI protein structure database^50^, in which phosphorylation sites were not explicitly specified (brown). **c**, Comparison of different SMO / PKA-C conformational states captured by AlphaFold 3 (blue) vs AlphaFold 2.3.0 (magenta or teal for conformations 1 or 2 (Conf1 or Conf2), respectively) (see Supplementary Discussion 2). Conf1 and Conf2 display near-identical positions for the PKI-like helix, RII-like helix, and reentrant loop, but with the PKA-C rotated such that it presents a new surface to the SMO 7TM domain. ipTM scores for Conf1 and Conf2 are 0.84 and 0.80, respectively. **d**, Left: empirical structures of agonist (SAG21k)-bound, active SMO (PDB: 6O3C) and PKA-C (PDB: 1ATP). Right: AlphaFold 3 model of the SMO / PKA-C complex. Note that in the AlphaFold model, the PKA-C N-terminus is pointing up towards the membrane. **e**, AlphaFold 3 model and PAE plot of the mouse SMO / PKA-C complex. **f**, HDX-MS data mapped onto conf2 of the SMO / PKA-C complex, displayed as in Fig. 2b in heatmap format. **g**, Multiple sequence alignment of helix 8 and pCT domains from SMO orthologs. Conserved K/R residues (blue circles), GRK2/3 phosphorylation sites (orange), and other essential residues for SMO / PKA-C signaling (green, see main text for detailed explanation) are indicated above the alignment, while secondary structural elements defined here or in prior studies are indicated below. Source data

Prior computational predictions suggest that the SMO pCT is an IDR^13^, consistent with the inability to resolve this region of SMO in cryo-EM^45^. A soluble portion of SMO encompassing almost the entire SMO pCT (residues 565–657) showed a mixed weak α-helical and random coil fingerprint in circular dichroism (CD) spectroscopy (Extended Data Fig. 1a), indicating limited secondary structure or an ensemble of disordered and partially structured states.

Whereas some IDRs are constitutively unstructured, others display conditional folding; they are unstructured in their apo, unmodified forms but adopt partially or fully structured conformations upon post-translational modification and/or binding to other proteins^37–39^. Although these ‘conditionally folded’ IDRs are challenging to characterize experimentally^36–38,40^, AlphaFold can often predict them accurately^47,48^ (Supplementary Discussion 1). Intriguingly, the full-length SMO model available from the EBI database of AlphaFold structure predictions^49,50^ shows several helical regions in the pCT (Extended Data Fig. 1b), suggestive of conditional folding. AlphaFold modeling may, therefore, provide structural insights into the SMO–PKA-C interaction.

We used AlphaFold to model mouse SMO bound to PKA-Cα (the best-studied and most ubiquitously expressed PKA-C isoform). We initially used AlphaFold 2.3.0 (ref. ^51^) but ultimately switched to AlphaFold3 (released during the final stages of this project)^52^, as version 3 enabled us to explicitly model SMO phosphorylation sites and Mg-ATP, all essential for SMO–PKA-C interactions^13–15^. The models produced by AlphaFold 2.3.0 and AlphaFold3 are similar, likely because the contributions from GRK2/3 phosphorylation are already reflected in the sequence–structure relationships in the multiple-sequence alignments^47^ (Supplementary Discussion 1 and Supplementary Data Files 1 and 2). AlphaFold 2.3.0 also predicts a slightly lower-confidence complex (Extended Data Fig. 1c), possibly an alternative conformation (Supplementary Discussion 2). Unless noted, we refer to the AlphaFold3 model below.

In the AlphaFold model, the phosphorylated SMO (pSMO)–PKA-C complex consists of an extended binding interface in which the SMO pCT and ICLs cradle PKA-C’s C-lobe and active-site cleft, with the kinase’s C-lobe pointing upward toward the SMO seven-transmembrane (7TM) domain (Fig. 1b). The complex harbors several key features (Fig. 1b). Within the pCT, the pseudosubstrate motif consists of an inhibitor sequence that engages the PKA-C active site, immediately preceded by an amphipathic helix (615–630) that we refer to as the ‘PKI-like helix’ (Fig. 1b,c) for its resemblance to one found in PKI proteins^29,53^. The PKI-like helix interacts at an approximately 30° angle with a second, longer amphipathic helix within the SMO pCT, which we designate the ‘RII-like helix’ (Fig. 1b,c), as it structurally mimics features of the PKA-RII holoenzyme^35^, as discussed below. The region between these helices contains several GRK2/3 phosphorylation sites along with a sequence, which we call the ‘reentrant loop’ (602–609), that interacts intramolecularly with a cavity formed by transmembrane helix 3 (TM3), TM6 and TM7 of the SMO 7TM domain (Fig. 1b,c). Lastly, other portions of the helices and ICLs from the SMO 7TM domain interact with PKA-C’s C-lobe, while the kinase’s N-terminal helical domain points toward the membrane (Extended Data Fig. 1d).

**Fig. 2:**
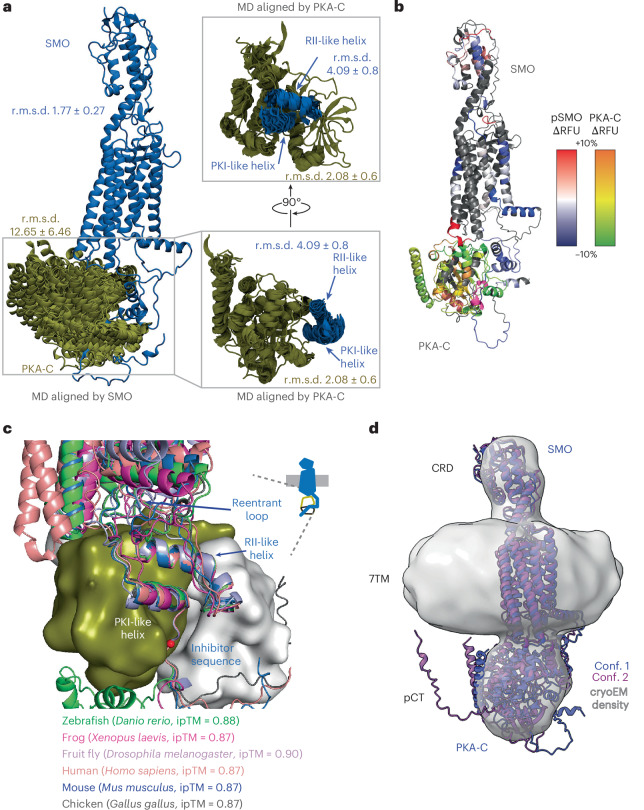
Overview of experimental, computational and evolutionary analysis of the SMO–PKA model. **a**, Structural snapshots of the complex between phosphorylated mouse SMO (blue) and PKA-C (olive) as assessed by MD simulations (one snapshot every 300 ns, 3 × 2 μs of simulation time). Note that MD simulations (unlike the AlphaFold models) include the SMO ligand cholesterol and the PKA-C *N*-myristoyl modification. Left, overall view of the complex, aligned on the SMO 7TM domain. Average r.m.s.d. values ± s.d. are shown for the backbone of the SMO 7TM domain and PKA-C helices. Right, zoomed-in view of the interface between PKA-C and SMO, aligned to the PKA-C helices, highlighting the stability of the PKI-like helix, inhibitor sequence and RII-like helix. **b**, Mapping of HDX-MS results onto the AlphaFold3 model of the SMO–PKA-C complex, rendered as a heat map. Peptides in SMO or PKA-C showing decreased or increased exchange (that is, increased or decreased protection) are shown in their respective color scales, indicated on the right. RFU, relative fractional uptake. **c**, AlphaFold3 models of SMO–PKA-C complexes from the indicated species, aligned on PKA-C from each complex. Note that, although the 7TM domain of human SMO appears slightly shifted compared to other SMO orthologs, this difference falls within the range of variability observed for this interface in the mouse complex (Extended Data Fig. 8d). **d**, Docking of two AlphaFold 2.3.0 models (Supplementary Discussion 2) into a low-resolution cryo-EM map obtained from an SMO–PKA-C sample that is reconstituted into MSP1E3D1 lipid nanodiscs. Conformations 1 and 2 are colored blue and dark magenta, respectively. Cryo-EM density is shown in gray and displayed at a contour level of 0.1.

Multiple lines of evidence support the accuracy of our AlphaFold model. Firstly, the model agrees with prior empirical structures of PKA-Cα^34,35,53,54^ and the SMO extracellular and 7TM domains^7,11,45,46^ (Extended Data Fig. 1d). Secondly, the highest-ranked model achieved an interface predicted template modeling (ipTM) score of 0.87, indicating high global confidence^51^, along with high (>70) predicted local distance difference test (pLDDT) values and low predicted aligned error (PAE) values indicating high local confidence in key interface-forming regions^55,56^ (Fig. 1b, Extended Data Fig. 1e and Supplementary Fig. 1). Thirdly, PKA-C remained bound to SMO in extended (3 × 2 µs) all-atom MD simulations and the pCT–PKA-C interface was highly stable despite flexibility at the 7TM–C-lobe contact (Fig. 2a and Supplementary Table 1), Fourthly, amide hydrogen–deuterium exchange mass spectrometry (HDX-MS) studies demonstrated protection of key binding interface elements (Fig. 2b and Extended Data Fig. 1f), as discussed below. Lastly, the SMO–PKA-C interface is conserved at the sequence and structural level across metazoans (Fig. 2c and Extended Data Fig. 1g).

**Extended Data Fig. 2:**
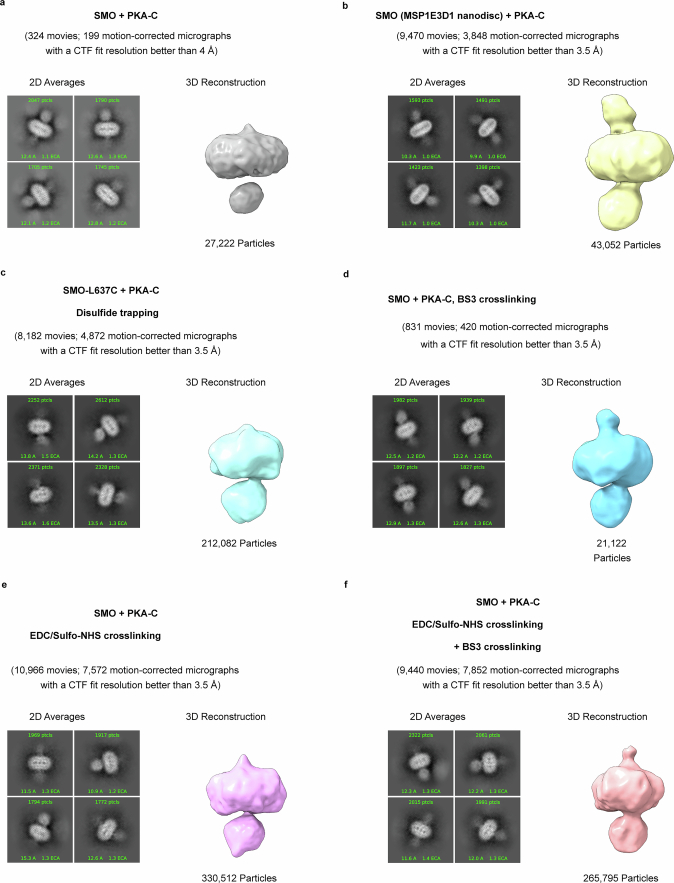
Representative 2D class averages and 3D reconstructions of the SMO / PKA-C cryo-EM samples. SMO / PKA-C mixed immediately prior to grid preparation (**a**), SMO / PKA-C complex in MSP1E3D1 nanodiscs (**b**, which is shown in Fig. 2d), Disulfide-trapped SMO-L637C / PKA-C complex (**c**), BS3-crosslinked SMO / PKA-C complex (**d**), EDC/Sulfo-NHS-crosslinked SMO / PKA-C complex (**e**), SMO / PKA-C complex subjected to dual EDC/Sulfo-NHS and BS3 crosslinking (**f**). All SMO samples were reconstituted in GDN detergent except for the nanodisc sample in **b**. The numbers of raw movies collected and motion-corrected micrographs with a CTF fit resolution better than 3.5 Å (or 4 Å, which were used for particle picking and extraction) are indicated for each sample. 2D class averages were derived from 500 or fewer selected micrographs for comparison purposes. Unsharpened cryoEM maps from Non-Uniform Refinement were roughly aligned to the same orientation and are displayed at suitable contour levels. Data processing details are provided in Supplementary Data File 3.

To directly visualize the purified SMO–PKA-C complex, we performed single-particle cryo-EM, collecting ~40,000 videos across seven sample conditions (Fig. 2d, Extended Data Fig. 2, Supplementary Table 2, Supplementary Fig. 2 and Supplementary Data File 3). The datasets converged on similar architectures, with a sample in MSP1E3D1 nanodiscs yielding slightly more detail than others (Extended Data Fig. 2, Supplementary Data File 3 and Supplementary Table 2). The map (8.57-Å nominal resolution) revealed a large central ellipsoid corresponding to the nanodisc, with two protruding lobes—one small and one large. Rigid-body fitting of the AlphaFold models showed good agreement with this cryo-EM density, supporting the overall architecture and relative positioning of SMO and PKA-C (Fig. 2d). Although the resolution is insufficient at present to visualize main-chain or side-chain features or to distinguish between the two conformations of the complex, these empirical structural data nevertheless demonstrate that AlphaFold models provide accurate initial representations of the SMO–PKA-C interaction and a starting point for biochemical and functional studies.

### A central role for the SMO pseudosubstrate motif in the SMO–PKA-C complex

**Fig. 3:**
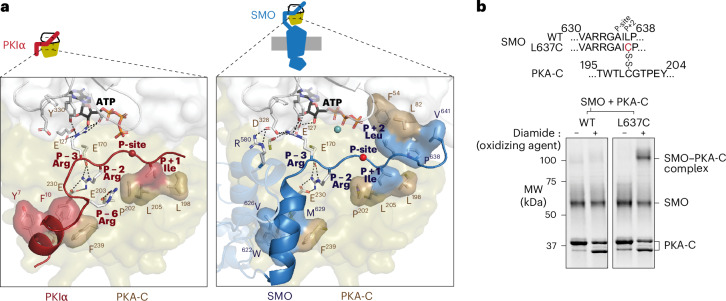
A central role for the SMO pseudosubstrate motif in the SMO–PKA-C complex. **a**, Left, empirical structure of PKIα (residues 5–24) (PDB 1ATP; red) in complex with PKA-C (olive and white, as in Fig. 1b). Right, AlphaFold model of pSMO (blue) in complex with PKA-C. In this and all subsequent figures, the SMO cartoon (top) represents the orientation of the SMO–PKA-C complex (in this case, with SMO at the bottom and PKA-C at the top, with the PKA-C N-lobe pointing upward and the C-lobe pointing downward). Locations of residues discussed in the text are indicated. Note the salt bridge between the P − 6 arginine in PKIα and PKA-C E203 (left); this interaction is absent in SMO (right). **b**, SDS–PAGE analysis of purified, near-full-length WT or L637C mutant SMO incubated with PKA-C treated with or without the oxidizing agent diamide (to induce disulfide bond formation). Sequence alignments above the gel image show the substitution present in SMO and the location of the disulfide bond between SMO L637C and PKA-C C199. The endogenous cysteine in the SMO intracellular domain (C554) in WT SMO provides a negative control for nonspecific SMO–PKA-C disulfide bond formation. The locations of SMO, PKA-C and the disulfide-trapped SMO–PKA-C complex are indicated on the right. PKA-C runs as a doublet following diamide treatment because of the formation of an intramolecular disulfide bond between C199 and C343, as shown previously^107,108^. Data are representative of *n* = 4 biological replicates. MW, molecular weight. Source data

In our model, the SMO pseudosubstrate motif forms the linchpin of the SMO–PKA-C interaction, with the inhibitor sequence adopting a canonical orientation between the kinase’s N and C lobes^53^. Like other PKA-C inhibitors^32,34,35,53^, the pseudosubstrate site (P-site) alanine, P − 2 and P − 3 arginines and P + 1 isoleucine residues interact extensively with the PKA-C active-site cleft (Fig. 3a). The P + 1 isoleucine (SMO I636) packs hydrophobically against PKA-C L198, P202 and L205 and the P + 2 leucine, P + 3 proline, and P + 6 valine (SMO L637, P638 and V641) pack hydrophobically against PKA-C L82 and F54 (Fig. 3a). In addition, SMO residues W622, V626 and M629, located in the PKI-like helix, hydrophobically pack against Y235 and F239 within the PKA-C αF–αG region, resembling interactions in PKIα^32,53^ (Fig. 3a). Consistent with our AlphaFold model, mutational analysis showed that the P − 2 and P − 3 arginines, P-site alanine and P − 13 tryptophan (W622) are essential for SMO–PKA-C interactions and Hh signal transduction^14^.

**Extended Data Fig. 3:**
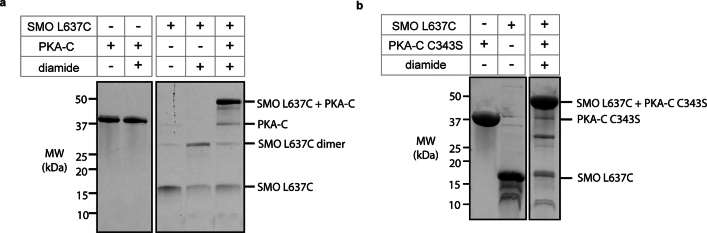
Evolutionary conservation of sequence and structural elements in SMO / PKA-C complexes. **a**, Disulfide trapping of PKA-Cα with soluble SMO pCT L637C (that is, lacking the CRD and 7TM domains), performed as in Fig. 3b. The SMO pCT has no endogenous cysteines, so trapping must be with L637C (the engineered Cys at P + 2). **b**, Comparable disulfide trapping occurs with a PKA-C C343S mutant. Because PKA-Cα has only two endogenous cysteines (C199 and C343), this result indicates that disulfide trapping involves PKA-C C199. Data are representative of n = 3 biological replicates with similar results. Source data

To further evaluate the predicted SMO–PKA-C binding mode, we performed disulfide trapping with the mouse SMO–PKA-C complex. In PKA-RII holoenzymes, the P + 2 cysteine forms a disulfide bond with PKA-C C199 under oxidizing conditions^57,58^. If the SMO and PKA-RII inhibitor sequences bind similarly to PKA-C, then substitution of the SMO P + 2 leucine with cysteine (L637C) should produce a disulfide bond between SMO and PKA-C C199, similar to the P + 2 cysteine in PKA-RII. Using purified proteins in vitro, we observed that PKA-C formed a disulfide trap with L637C versions of near-full-length SMO or the isolated pCT, whereas wild-type (WT) SMO exhibited little if any trapping (Fig. 3b and Extended Data Fig. 3a,b).

Whereas PKI proteins are constitutive PKA-C inhibitors^29,31^, only the active, GRK2/3-phosphorylated conformation of SMO can bind and inhibit PKA-C under cellular conditions^13,15^. Accordingly, peptides encompassing the minimal active-site-binding portion of PKIα bind PKA-C with low nanomolar affinity^59^ but a peptide encompassing the corresponding region of SMO binds ~1,000-fold more weakly^14^. The PKI-like helix in SMO is elongated and reorientated in our models compared to the one in PKIα, eliminating a key salt bridge (PKIα P − 6 arginine to PKA-C E203)^53^ (Fig. 3a). This reorientation likely accounts for the lower affinity of the SMO pseudosubstrate motif.

Thus, our AlphaFold model provides a high-confidence structural prediction of the SMO–PKA-C complex structure and establishes the central role of SMO’s PKA pseudosubstrate motif in mediating this interaction.

### SMO structurally mimics the PKA-RIIβ holoenzyme

**Fig. 4:**
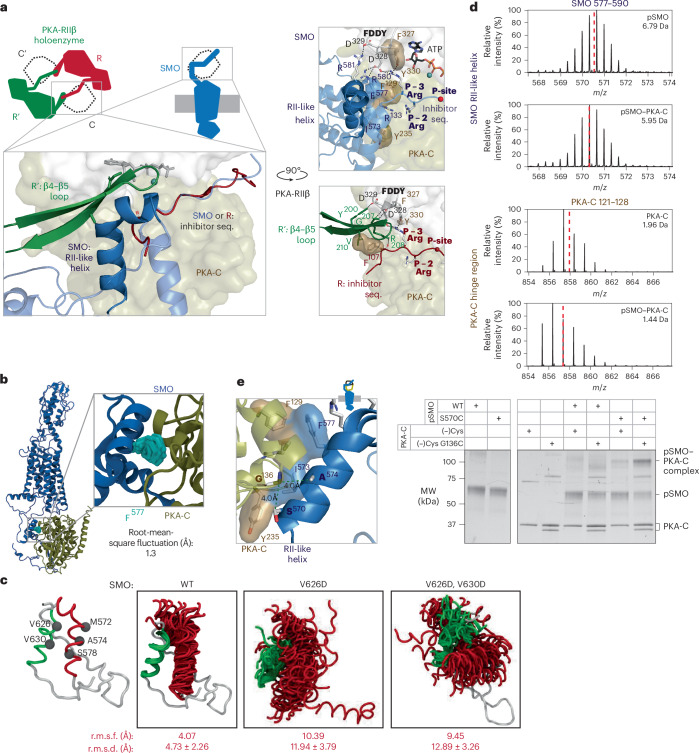
Structural mimicry of the PKA-RIIβ holoenzyme by an SMO amphipathic helix. **a**, Top, cartoon of tetrameric PKA-RIIβ holoenzyme (RIIβ_2_-C_2_; left cartoon) where each PKA-C (C; dotted outline) contacts the inhibitor sequence of one PKA-R (R; red) and the β4–β5 loop of the other (R′; green) and vice versa. Bottom, overlay between PKA-RIIβ holoenzyme empirical structure (PDB 3TNP) and pSMO–PKA-C AlphaFold3 model aligned on the PKA-C in each complex. SMO is shown in blue, with the RII-like helix in foreground (dark blue) and additional SMO sequences, including inhibitor sequence and PKI-like helix, in background (light blue). Right, SMO and PKA-RIIβ complexes shown separately, with key residues and motifs indicated. **b**, MD snapshots of SMO F577 (light blue, one every 150 ns, 3 × 2 μs of simulation time) within the SMO C-tail (dark blue); frames are aligned on the PKA-C helical backbone (brown). **c**, MD structural snapshots (one every 30 ns, 3 × 500 ns of simulation time) show the positions of the PKI-like helix (residues 615–630; green) and RII-like helix (residues 570–581; red) during simulations of WT SMO or the indicated SMO mutants (model on the left). Simulations are aligned using the backbone of SMO residues 615–630. Overall root-mean-square fluctuations (r.m.s.f., in Å) and r.m.s.d. for backbones of indicated residues are shown below each simulation. **d**, Representative HDX-MS envelope (deuterium exchange time *t*_ex_ = 10 min) of peptides in the indicated region in pSMO or PKA-C alone (top spectrum in each pair) or in complex with PKA-C (bottom spectrum in each pair). Top right, deuterium uptake shown for each spectrum. Centroids are indicated by red dashed lines. SMO peptide, residues 577–590; PKA-C peptide, residues 121–128. **e**, Right, SDS–PAGE analysis of diamide-induced disulfide trapping between pSMO S570C and PKA-C G136C in a (−)Cys background (Methods). WT SMO and (−)Cys PKA-C served as negative controls. Left, locations of SMO S570, A574 and PKA-C G136 indicated on cartoon structure. Quantification is shown in Fig. 6e. Data are representative of *n* = 3 biological replicates. Source data

To bind PKA-C with high affinity, PKA-R subunits combine a ‘central’ interaction at the PKA-C active-site cleft with ‘auxiliary’ interactions engaging additional PKA-C surfaces^2,28,30^. In the PKA-RIIβ holoenzyme (RIIβ_2_-C_2_), the inhibitor sequence from one PKA-RIIβ subunit binds the PKA-C active-site cleft, while the β4–β5 loop from the other PKA-RIIβ subunit engages the PKA-C hinge region (between the kinase’s N-lobe and C-lobe)^60^ and ‘FDDY’ motif essential for ATP binding^35^ (Fig. 4a). Together, these central and auxiliary interactions lock PKA-C into a closed conformation and interrupt catalysis^2,28,30^.

Surprisingly, a 12-aa SMO amphipathic helix (mouse SMO, residues 570–581), which we term the RII-like helix, mimics key structural features of both PKA-R subunits in the PKA-RIIβ holoenzyme. Despite lacking sequence or structural homology to PKA-RIIβ, this SMO helix binds to the same regions engaged by the β4–β5 loop of one PKA-RIIβ subunit and the backside of the inhibitor sequence from the other^35^ (Fig. 4a). Furthermore, an analogous pattern of hydrophobic and hydrogen-bonding interactions underlies both SMO–PKA-C and PKA-RIIβ–PKA-C complexes. SMO F577 hydrophobically packs against PKA-C F129 and R133, mirroring the hydrophobic interactions of F107 at the P − 5 site from the PKA-RIIβ inhibitor sequence and V210 from the β4–β5 loop^35^ (Fig. 4a). SMO R580 and R581 hydrogen bond with D328/D329 from the FDDY motif, mimicking interactions between PKA-RIIβ Y200, G207 and R208 with these same PKA-C residues^35^ (Fig. 4a). A cation–*π* interaction between SMO R580 and SMO F577 is expected to enhance these ionic interactions (Fig. 4a). Additional residues contributing to this region of the SMO–PKA-C complex are summarized in Fig. 4a. Thus, despite distinct primary sequences, secondary-structure elements and binding stoichiometries, SMO and PKA-RIIβ use remarkably similar central and auxiliary interactions to engage PKA-C.

**Extended Data Fig. 4:**
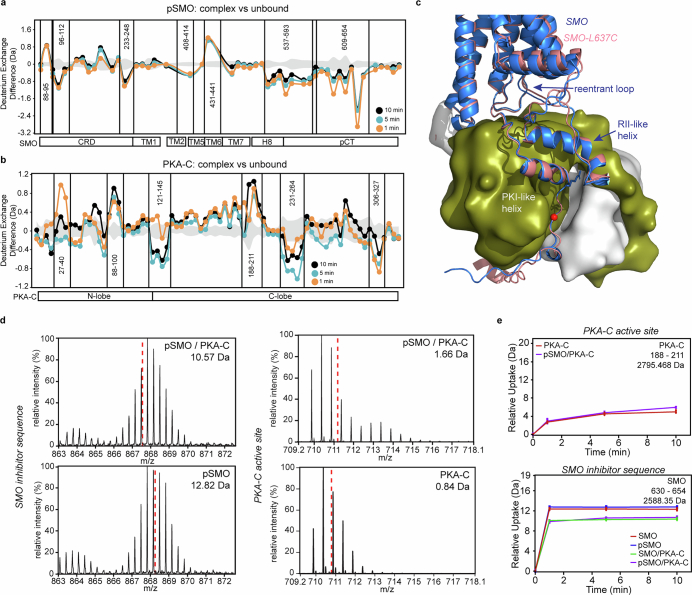
HDX-MS studies of phosphorylated SMO / PKA-C complexes vs. unbound proteins. Deuterium exchange difference (average number of deuterons) mapped for SMO (**a**) or PKA-C (**b**) in the phosphorylated SMO (pSMO) / PKA-C complex vs. the unbound protein, at the indicated timepoints following deuterium exchange. Key peptides are indicated on the plot, Negative differences denote decreased exchange (that is, increased protection) in the complexed vs uncomplexed forms. Domain organization of SMO and PKA-C are indicated below each plot: CRD = cysteine rich domain, TM = transmembrane helix (numbered 1–7), pCT = membrane proximal C-tail. Standard deviations from replicate measurements are in gray. **c**, Superposition of PKA-C complexes with wild-type SMO (blue) or SMO L637C (salmon), with key peripheral SMO structural elements indicated. P-site alanine is red. Although the PKI-like helix and RII-like helix are near-superimposable in both models, the reentrant loop is in slightly different positions within the SMO 7TM cavity; however, this is within the range of variability observed for this region even in the wild-type protein (see Fig. 7d, Extended Data Fig. 8b). Representative HDX-MS mass spectral envelope (t = 10 min) (**d**) or uptake vs. time plots (**e**) of the indicated peptides within the PKA-C active-site cleft or the SMO inhibitor sequence.

**Extended Data Fig. 5:**
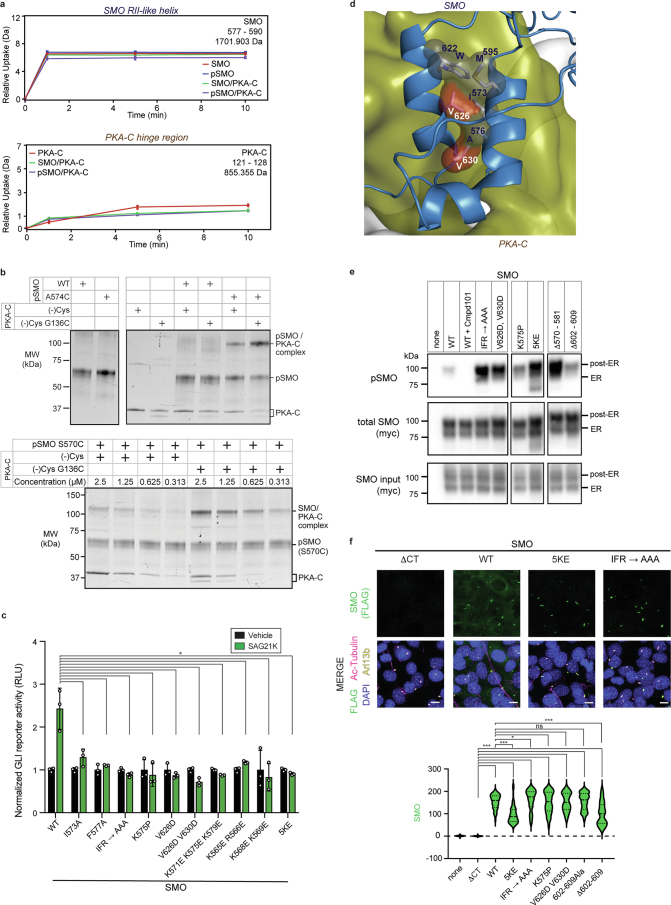
Additional biochemical, cell biological, and functional studies of the SMO RII-like helix. **a**, HDX-MS uptake vs. time plots for peptides in the SMO RII-like helix (top) and PKA-C hinge region (bottom). **b**, Top: SDS-PAGE analysis of diamide-induced disulfide trapping between minimal-cysteine PKA-C and SMO A574C, as in Fig. 4e. Data are representative of n = 4 replicates with similar results. Bottom: Optimization of disulfide trapping signal-to-noise. Fixed amounts of pSMO S570C were titrated against varying concentrations of PKA-C lacking (lanes 1–4) or containing (lanes 5–8) the G136C mutation. Trapping was consistently greater for the G136C mutant at each concentration tested. Data are representative of n = 3 replicates with similar results. **c**, GLI transcriptional reporter assay using wild-type or mutant SMO constructs in Smo-/- MEFs, performed as in Fig. 5a with SAG21k (green) or vehicle (black). Data represent mean ± s.d., n = 3 independent biological replicates. **d**, Locations of SMO V626 and V630 (red) and interacting residues at the PKI-like/RII-like helix interface. **e**, SAG21k-induced phosphorylation of myc-tagged SMO constructs (or empty vector) in HEK293 cells following anti-myc pulldown and immunoblotting with anti-pSMO (top) and anti-myc (bottom). GRK2/3 inhibitor Compound 101 (Cmpd101) serves as a negative control. Note that although several of these SMO mutants exhibit stronger phosphorylation than the wild-type protein, none of them exhibit less phosphorylation, making it unlikely that their deficits in GLI transcriptional reporter assays arise from an inability to undergo GRK2/3-mediated phosphorylation. Data are representative of n = 3 replicates with similar results. **f**, Ciliary localization of FLAG-tagged SMO constructs in NIH3T3 cells following SAG21k treatment. Top: representative images (FLAG-SMO, green; AcTub, magenta; Arl13b, yellow; DAPI, blue). SMOΔCT serves as a negative control. Bottom: quantification of FLAG-SMO localization to primary cilia (n = 100 cells per condition, two independent experiments). Mutants show at best only modest (<50%) reductions in ciliary localization relative to wild-type SMO. Scale bar = 10 μm. P < 0.05 (*); P < 0.001 (**); ns = not significant. See Supplementary Table 4 for full statistical analysis. Source data

The SMO PKI-like helix, inhibitor sequence and RII-like helix remain stably engaged with the PKA-C hinge region and active-site cleft during all-atom MD simulations, with SMO F577 exhibiting marked stability (Figs. 2a and 4b,c). These observations are indicative of an energetically favorable binding configuration. To empirically define the binding interface, we used HDX-MS (Extended Data Fig. 4a,b, Supplementary Table 3, Supplementary Figs. 3 and 4 and Supplementary Discussion 3)^61–64^ on an SMO–PKA-C complex disulfide-trapped at the interface between the SMO pseudosubstrate motif and PKA-C active-site cleft (SMO L637C + PKA-C C199; Fig. 3b); this highly specific disulfide bond is expected to stabilize the complex at its central interaction while allowing other peripheral interactions to form freely (Extended Data Fig. 4c), thereby facilitating HDX-MS of this likely unstable complex. HDX-MS analysis of the pSMO–PKA-C complex revealed deuterium exchange protection in the SMO pseudosubstrate motif and the PKA-C active-site cleft relative to the uncomplexed proteins (Extended Data Fig. 4d,e and Supplementary Discussion 3). The SMO RII-like helix (577–590) and the PKA-C hinge region (122–128) also showed significant deuterium exchange protection (>0.5 Da) in the complex (Fig. 4d and Extended Data Fig. 5a). Overall, these findings support binding of SMO RII-like helix to the PKA-C C-lobe as predicted by our model.

To directly evaluate the binding between the SMO RII-like helix and the PKA-C hinge region with single-residue precision, we conducted additional disulfide-trapping studies. In our model, SMO S570 and A574 on the hydrophobic face of the RII-like helix reside within 2.7 and 4.4 Å of PKA-C G136, respectively. Accordingly, purified, pSMO S570C or A574C formed a specific disulfide bond with PKA-C G136C (Fig. 4e, Extended Data Fig. 5b and Supplementary Discussion 4), demonstrating that the SMO RII-like helix directly contacts the PKA-C hinge region, consistent with the AlphaFold model.

**Fig. 5:**
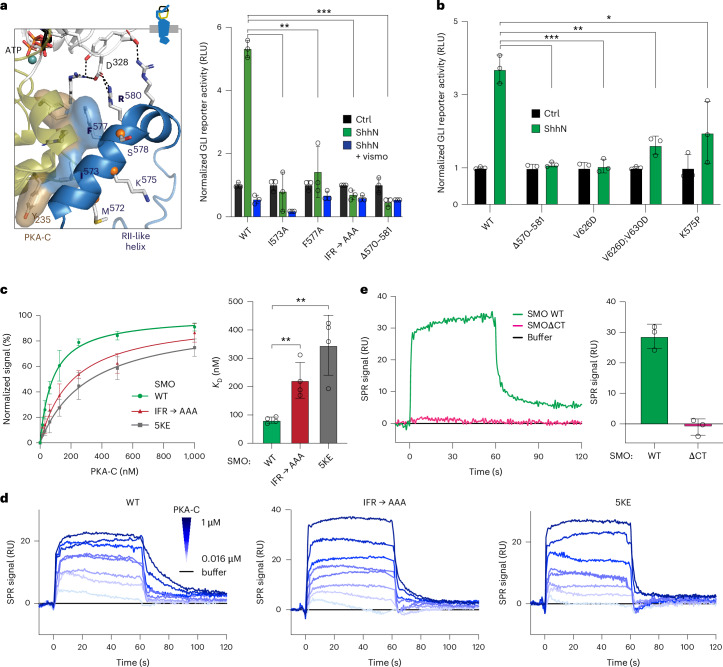
The SMO RII-like helix is essential for Hh signal transduction. **a**, GLI transcriptional reporter assay in *Smo*^−/−^ MEFs transfected with GFP (negative control), WT SMO or the indicated SMO mutant constructs. Cells were treated with conditioned medium containing the N-terminal signaling domain of Sonic Hh (ShhN) alone (green), or in the presence of the SMO inverse agonist vismodegib (ShhN + vismo; blue) or control conditioned medium lacking ShhN (black). Data are normalized to the expression of each mutant relative to WT SMO (Methods) and represent the mean ± s.d. (*n* = 3 independent biological replicates). Locations of SMO residues are indicated in the cartoon at left. **b**, A second panel of SMO mutants were analyzed as in (**a**). Data represent the mean ± s.d. (*n* = 3 independent biological replicates). **c**, Left, steady-state SPR analysis of binding interactions between PKA-C and WT SMO or the indicated SMO mutants reconstituted into nanodiscs (definition and functional characterization of the 5KE mutant in Fig. 6). Data represent the mean ± s.d. (*n* = 3 independent biological replicates). Right, *K*_D_ determinations for the experiments shown on the left (*n* = 4 measurements per condition). **d**, SPR sensorgram for binding of myristoylated PKA-Cα, in concentrations ranging from 0.016 μM to 1 μM, to phosphorylated WT SMO or the indicated SMO mutants reconstituted into nanodiscs. **e**, Left, SPR sensorgram for 250 nM PKA-Cα binding to WT SMO (green) but displaying no binding to a negative control version of SMO lacking the entire CT (SMOΔCT, magenta), consistent with prior findings^13,15^. Right, quantification of SMO WT or SMOΔCT SPR binding signal with data representing the mean ± s.d. (*n* = 3 biological replicates). *P < 0.05, **P < 0.01 and ***P < 0.001. Full statistical analysis is presented in Supplementary Table 4. RLU, relative luminescence units. Source data

To assess the impact of the SMO RII-like helix on SMO–PKA-C interactions and Hh signal transduction, we used mutational analysis. Prior systematic SMO scanning mutagenesis identified eight amino acids that, when substituted individually to alanine (or glycine, for alanines in the WT sequence), prevent Hh-induced activation of a GLI transcriptional reporter, through an unknown mechanism^65^. Six of these eight residues are within the SMO RII-like helix and four of the six (S570, I573, F577 and R580) directly engage PKA-C in our model (Fig. 5a). We confirmed that I573A or F577A, alone or combined with R580A (IFR→AAA), abolished GLI reporter activation (Fig. 5a and Extended Data Fig. 5c). The remaining substitutions (A574G and A576G)^65^ likely hindered formation of the RII-like helix, as glycine is a helix-destabilizing residue. Accordingly, whereas alanine substitution of SMO K575, a non-PKA-C-interacting residue within the RII-like helix, only modestly (<50%) decreased GLI reporter activation^65^, substitution with proline, a potent helix disruptor, had a markedly stronger effect (Fig. 5b and Extended Data Fig. 5c). Lastly, destabilizing the hydrophobic interface between the RII-like helix and the PKI-like helix (V626D;V630D; Fig. 4c and Extended Data Fig. 5d) strongly blocked GLI transcriptional activation (Fig. 5b and Extended Data Fig. 5c). Control experiments established that the inability of these mutants to signal cannot be explained by defects in SMO expression, ciliary localization or agonist-induced GRK2/3 phosphorylation (Extended Data Fig. 5e,f), consistent with previous findings^13,65,66^.

A parsimonious explanation for the Hh signaling loss-of-function mutations in the SMO RII-like and PKI-like helices is that these mutations compromise a key interface between the SMO RII-like helix and PKA-C hinge region. Consistent with this hypothesis, the region of SMO encompassing the RII-like helix (570–581) is critical for interactions with PKA-C in bioluminescence resonance energy transfer assays^13^. To quantify this interaction, we developed a surface plasmon resonance (SPR) assay using near-full-length, GRK2/3-phosphorylated SMO reconstituted into nanodiscs in vitro. WT pSMO bound PKA-C with high affinity (*K*_D_ = 82 ± 11 nM), while the IFR→AAA substitution significantly weakened the interaction (*K*_D_ = 222 ± 63 nM) (Fig. 5c–e and Supplementary Discussion 5). The effects of the substitution were less pronounced in SPR than in GLI reporter assays (Fig. 5a), possibly reflecting differences in each assay’s sensitivity and dynamic range (Supplementary Discussion 5). Nevertheless, these results support a key role for the RII-like helix in mediating SMO–PKA-C binding.

In sum, AlphaFold modeling, HDX-MS, disulfide-trapping and mutational analyses demonstrate that SMO structurally mimics the PKA-RIIβ subunit in PKA holoenzymes.

### Phosphorylation generates structural elements that stabilize the SMO–PKA-C complex

**Extended Data Fig. 6:**
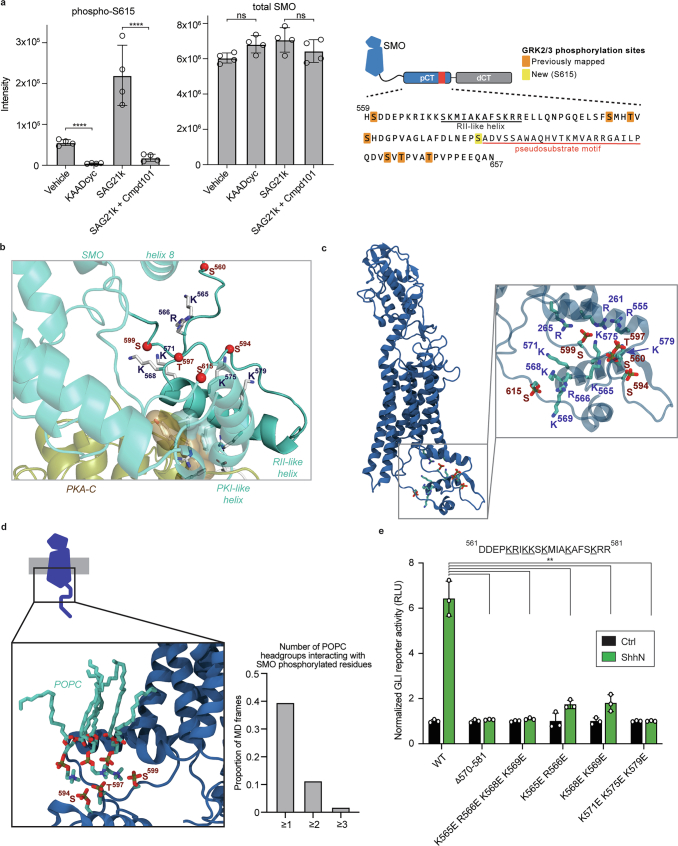
Additional biochemical and functional studies of SMO phosphorylation. **a**, Targeted MS-based quantification of phosphorylation of a phosphopeptide including pSMO 615 (left) and total SMO protein in each sample (middle), using FLAG-SMO protein isolated from HEK293 cells treated with vehicle, SMO agonist SAG21k, SMO inverse agonist KAADcyc, or GRK2/3 inhibitor Cmpd101. Data represent the mean ± s.d., n = 4 independent biological replicates. SMO sequence diagram indicating the location of S615 (yellow) along with the GRK2/3 phosphorylation sites previously mapped via MS^13^, is shown at right. ‘Intensity’ is a measurement of the abundance of phosphorylation sites (left) or total protein (right), derived from model-based estimation in Msstats^139^ which combines individual peptide intensities. Inset depicts SMO pCT sequence with new (yellow) and previously mapped^13^ (orange) GRK2/3 phosphorylation sites. **b**, Location of GRK2/3-phosphorylated S/T residues and K/R residues in the alternative conformation of the SMO / PKA-C complex (AlphaFold 2.3.0 Conf2, see Extended Data Fig. 1c). **c**, Locations of positively charged (K/R) and phosphorylated (S/T) SMO C-tail residues highlighted in the MD contact maps (Fig. 6b), mapped onto a structural snapshot from the SMO / PKA-C MD simulations**. d**, Structural snapshot showing positively charged POPC headgroups interacting with phosphorylated residues of the SMO C-tail. Graph shows quantification of interactions between SMO and the POPC headgroups, expressed as the fraction of frames in which at least one headgroup was located within 4 Å of a studied phosphate group. **e**, GLI transcriptional reporter assay on *Smo*^*−/−*^ MEFs transfected with the indicated SMO mutants and treated/analyzed as in Fig. 6c. SMO sequence from 561–581 is indicated, and residues mutated in this experiment are underlined. Data represent the mean ± s.d., n = 3 independent biological replicates. P < 0.01 (**); P < 0.0001 (****); ns = not significant. See Supplementary Table 4 for full statistical analysis. Source data

GRK2/3 dramatically enhances the SMO–PKA-C interaction^15^ by phosphorylating active SMO at eight conserved serines and threonines within the pCT^13,15^ (Extended Data Fig. 6a). Five (pS560, pS594, pT597, pS599 and pS615) reside N-terminal to the PKI-like helix, while three (pS642, pT644 and pT648) are C-terminal to the inhibitor sequence (Fig. 1c). Our AlphaFold models immediately suggest how phosphorylation in each region promotes SMO–PKA-C binding.

**Fig. 6:**
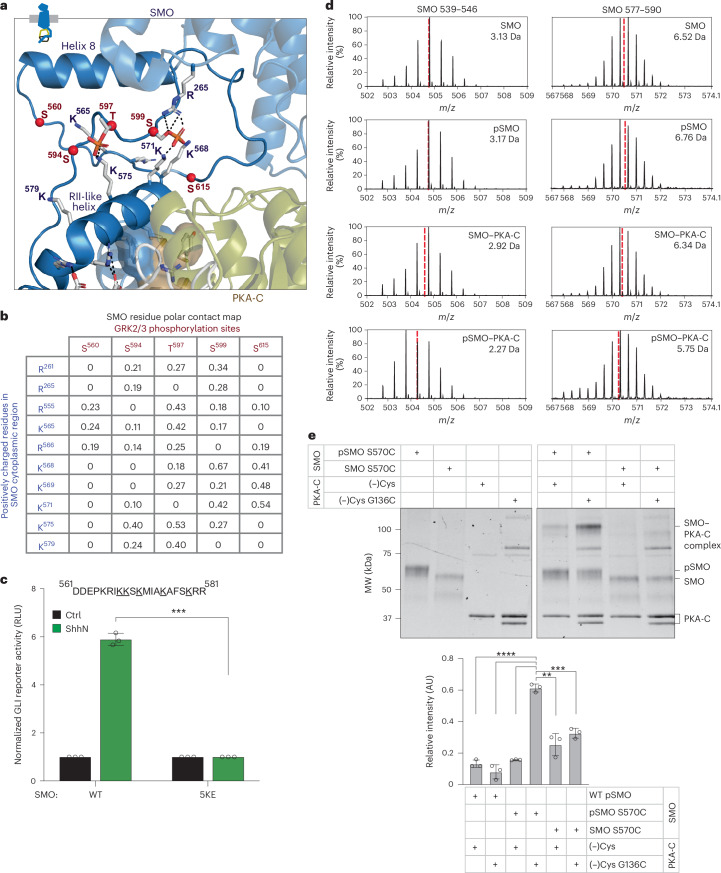
SMO phosphorylation generates structural elements that stabilize the PKA-C complex. **a**, AlphaFold model of the SMO–PKA-C complex (SMO in blue, PKA-C in olive and white), with GRK2/3-phosphorylated S/T residues (red) and conserved K/R residues highlighted in the SMO pCT. SMO helix 8 and the RII-like helix are indicated for orientation. **b**, Contact maps for GRK2/3-phosphorylated residues (red; each column is a distinct residue) and K/R residues (blue, each row is a distinct residue) in SMO intracellular regions, quantified from MD simulations of the SMO–PKA-C complex. Contact occupancies during the simulation (0–1, corresponding to 0–100%) were computed using the getcontacts module (Methods). **c**, WT SMO or the 5KE mutant (underlined K/R residues substituted to E) were analyzed using a GLI transcriptional reporter assay in *Smo*^*−/−*^ MEFs, performed as in Fig. 5b. Data represent the mean ± s.d. (*n* = 3 independent biological replicates). **d**, Representative HDX-MS envelope (*t*_ex_ = 5 min) of the indicated peptide in SMO helix 8 (residues 539–546) or the RII-like helix (residues 577–590) in SMO–PKA-C (third row) versus pSMO–PKA-C (fourth row). The deuterium uptake is shown for each spectrum to the top right. Centroids are indicated by red dashed lines. **e**, Disulfide trapping of PKA-C WT or G136C (in a (−)Cys construct) with phosphorylated versus nonphosphorylated, SAG21k-bound SMO (pSMO versus SMO, respectively), as in Fig. 4e. Bottom, quantification of band intensity. Data represent the mean ± s.d. (*n* = 3 biological replicates). Representative gel image corresponding to disulfide trapping of phosphorylated WT versus S570C mutant pSMO is shown in Fig. 4e. **P < 0.01, ***P < 0.001 and ****P < 0.0001. Full statistical analysis is presented in Supplementary Table 4. Source data

The five N-terminal phosphorylated residues cluster near a conserved stretch of lysines and arginines (K/R) (Fig. 6a and Extended Data Fig. 6b). We hypothesize that phosphorylation fosters electrostatic interactions between the negatively charged phosphoserine and phosphothreonine (pS/pT) and positively charged K/R residues. This will constrain the otherwise disordered SMO cytoplasmic tail into a more compact, ordered state conducive to forming secondary structures in the PKA-C-binding interface, namely the RII-like helix and the PKI-like helix. In this manner, a phosphorylation-driven intramolecular electrostatic switch in SMO drives SMO–PKA-C interactions.

**Extended Data Fig. 7:**
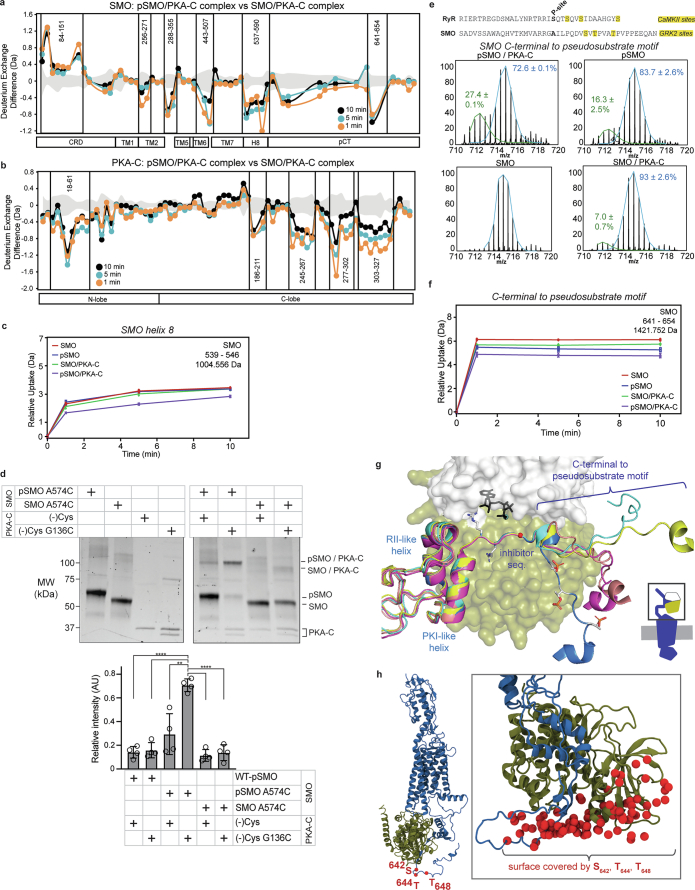
HDX-MS and disulfide trapping studies to monitor effects of phosphorylation on SMO / PKA-C complexes. Deuterium exchange difference (average number of deuterons, presented as in Extended Data Fig. 4) mapped for SMO (**a**) or PKA-C (**b**) in the phosphorylated vs nonphosphorylated SMO / PKA-C complex. **c**, HDX-MS uptake vs. time plots for the indicated peptide in SMO helix 8. **d**, Disulfide trapping of SAG21k-bound, phosphorylated vs nonphosphorylated SMO A574C / PKA-C complex, performed as in Fig. 6e. Quantification is below the gel and represents the mean ± s.d. from 4 biological replicates. **e**, HDX-MS uptake vs. time plots for the indicated peptide in the SMO region C-terminal to the pseudosubstrate motif. GRK2/3 phosphorylation sites in SMO, and CaMKII phosphorylation sites in RyR, are highlighted in yellow. Phosphorylated (pSMO) and non-phosphorylated (SMO) forms of SMO are displayed on the top or bottom row, respectively. Bimodal deconvolution of the peptide revealed high-exchanging (blue) and low-exchanging (green) populations, suggesting that this region may interact with PKA-C via a conformational selection mechanism. **f**, HDX-MS uptake vs. time plots for the indicated peptide C-terminal to the pseudosubstrate motif (see **e**). **g**, Overlay of top 5 AlphaFold 3 models indicating a propensity of the region C-terminal to the pseudosubstrate motif to wrap around PKA-C in some cases. **h**, Left: Positions of phosphorylated residues within this region of SMO, mapped onto a snapshot from the SMO / PKA-C MD simulations. Right: Structural flexibility of the phosphorylated SMO residues (S642, T644, T648) across multiple frames from MD simulations, leading to many transient contacts distributed across the surface of PKA-C’s N-lobe (one snapshot every 200 ns, 30 snapshots in total). P < 0.01 (**); P < 0.0001 (****). See Supplementary Table 4 for full statistical analysis. Source data

Several results support our proposed mechanism. First, MD simulations show that pSMO residues form polar interactions with nearby K/R residues (Fig. 6b and Extended Data Fig. 6c) and positively charged lipid headgroups (Extended Data Fig. 6d). Second, substitution of the SMO K/R residues (5KE) reduced PKA-C affinity (Fig. 5d,e) and eliminated GLI reporter activation (Fig. 6c and Extended Data Fig. 6e), without blocking SMO expression, trafficking or phosphorylation (Extended Data Fig. 5e,f). Third, HDX-MS of pSMO–PKA-C versus SMO–PKA-C complexes (Supplementary Discussion 3) revealed phosphorylation-dependent protection (>0.5 Da) in a peptide comprising the majority of the SMO RII-like helix (residues 577–590) and another comprising most of the SMO amphipathic helix 8 (residues 539–546) (Fig. 6d and Extended Data Figs. 5a and 7a–c), which runs parallel to the membrane and lies just above the pS/pT and K/R residues (Fig. 6a). Lastly, disulfide trapping between the SMO RII-like helix (S570C or A574C) and the PKA-C hinge (G136C) is weaker when SMO is not phosphorylated by GRK2/3 (trapping is 54.0% dependent on phosphorylation for S570C) (Fig. 6e and Extended Data Fig. 7d), directly demonstrating that phosphorylation stabilizes an essential SMO–PKA-C interface.

In addition to these five GRK2/3-phosphorylated residues, SMO contains three GRK2/3-phosphorylated residues C-terminal to the pseudosubstrate motif. A similar arrangement occurs in the ryanodine receptor (RyR), a PKA-C substrate, where phosphorylation of Ca^2+^-calmodulin-dependent protein kinase 2 (CaMKII) sites C-terminal to the PKA-C substrate motif (Extended Data Fig. 7e) enhances RyR–PKA-C interactions by stabilizing PKA-C-interacting structural elements^67^. The corresponding region of SMO is strongly protected upon GRK2/3 phosphorylation in HDX-MS studies, consistent with a disorder-to-order transition, and this protection is enhanced when PKA-C is present (Extended Data Fig. 7e,f), suggesting that GRK2/3 phosphorylation in this region may similarly enhance PKA-C interactions. While our AlphaFold models are of limited confidence in this SMO region, they hint that these GRK2/3-phosphorylated residues may help SMO wrap around PKA-C (Extended Data Fig. 7g). Accordingly, MD simulations revealed SMO conformations that span a broad surface of the PKA-C N-lobe (Extended Data Fig. 7h), potentially stabilizing the SMO–PKA-C complex.

Thus, SMO phosphorylation promotes intramolecular and intermolecular electrostatic interactions that stabilize conditionally folded regions within the SMO cytoplasmic domain, thereby remodeling it into a PKA-C-inhibiting conformation.

### Structural mimicry of canonical GPCR–effector interactions by the SMO–PKA-C complex

**Fig. 7:**
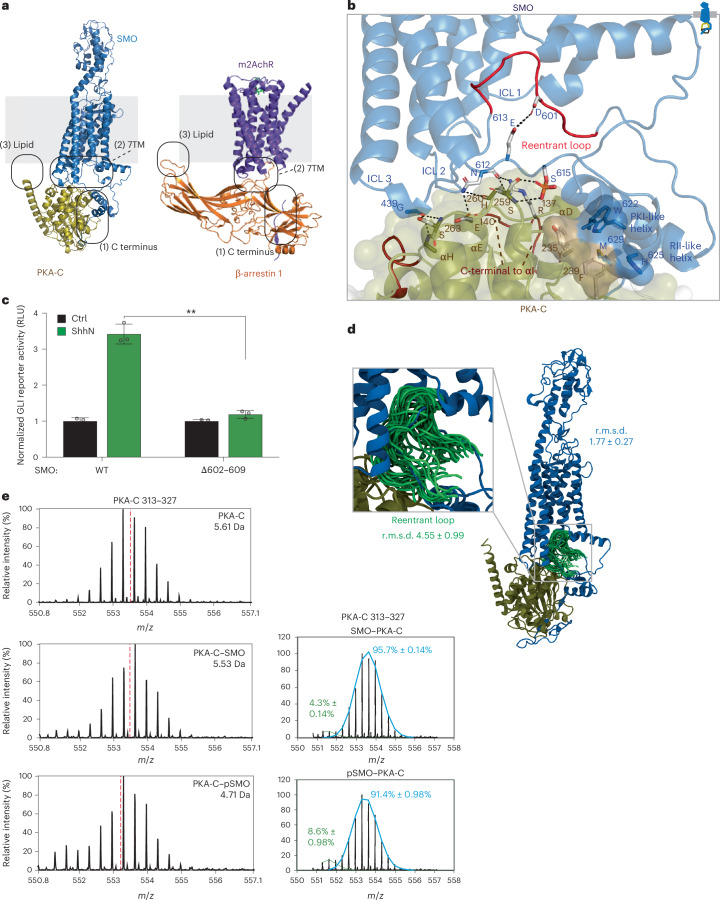
Structural mimicry of canonical GPCR–effector signaling complexes. **a**, Comparison of the SMO–PKA-C model (left) with the M2AchR–β-arrestin 1 structure (right). Locations of interaction sites in the CT (including the reentrant loop), 7TM domain (including ICLs) and membrane are indicated in each complex. **b**, The SMO 7TM interface. Residues in the αD–αE and αG–αH loops of PKA-C interacting with SMO ICL domains are indicated. The reentrant loop region of the SMO pCT (residues 602–609) is shown in red. **c**, GLI reporter assay for the indicated deletions in the SMO reentrant loop (**b**). Data represent the mean ± s.d. (*n* = 3 independent biological replicates). **d**, Structural snapshots of the reentrant loop (green; residues 599–614) in the complex between pSMO (blue) and PKA-C (olive) (one snapshot every 300 ns, 3 × 2 μs of simulation time). Each frame is aligned on the SMO 7TM region. **e**, HDX-MS data for region of PKA-C C-terminal to the αI helix (residues 313–327) in its apo (top), SMO-bound (middle) or pSMO-bound (bottom) forms, determined at *t*_ex_ = 10 min and plotted as in Fig. 4d. Bimodal deconvolution of the PKA-C (residues 313–327) spectrum revealed high-exchanging (blue) and low-exchanging (green) populations, suggesting two noninterconverting states of the SMO–PKA-C complex. **P < 0.01. Full statistical analysis is presented in Supplementary Table 4. Source data

Beyond structural similarities of SMO to classical PKA-C inhibitors (PKI proteins and PKA-R subunits), the SMO–PKA-C complex structurally mimics canonical GPCR–effector assemblies^19,20,27^. Comparing our SMO–PKA-C model to one such assembly, the M2 muscarinic receptor (M2AchR)–β-arrestin 1 complex^68^ reveals an overall architectural similarity (Fig. 7a), as well as similar contributions of the 7TM domain and membrane lipids.

**Extended Data Fig. 8:**
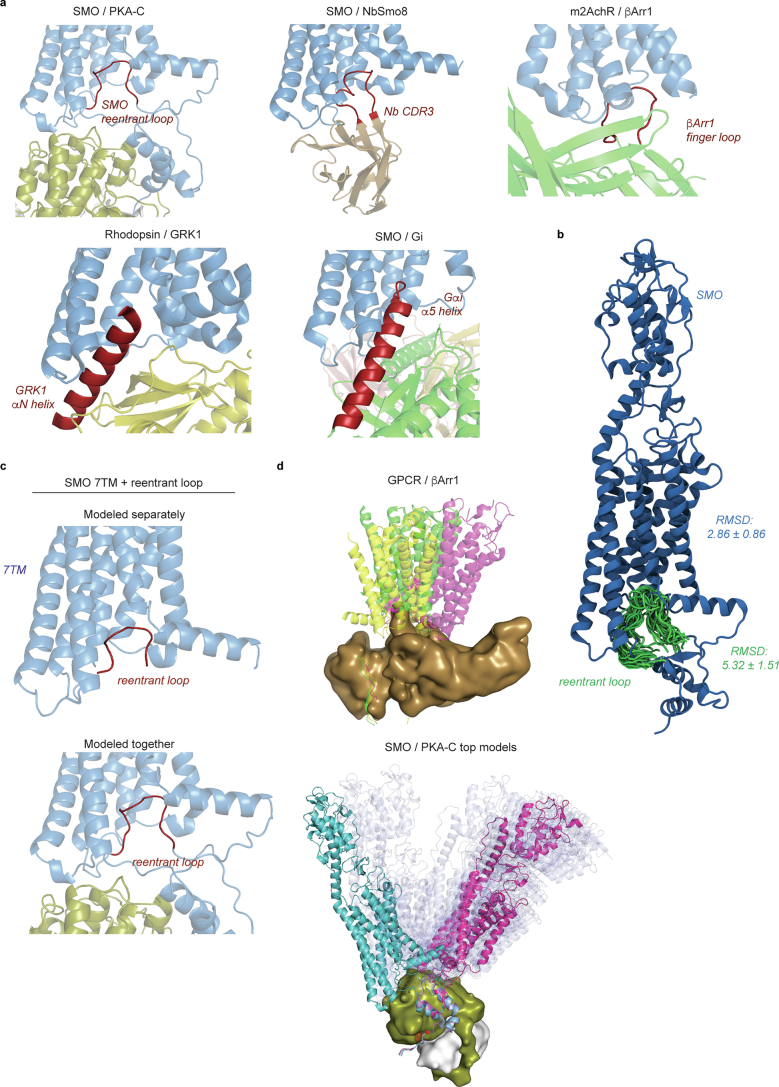
The SMO / PKA-C complex structurally mimics other GPCR-effector complexes. **a**, Comparison of the SMO / PKA-C complex to SMO or other GPCRs bound to the indicated proteins. In each structure, the GPCR portion is colored cyan, and the structural elements that engage the intracellular 7TM cavity are colored maroon. PDB numbers: SMO/NbSmo8 (6O3C), m2AchR/Barr1 (6U1N), Rhodopsin/GRK1 (7MT9), SMO/Gi (6XBL). **b**, MD simulations of SMO alone, without PKA-C, Snapshots of the reentrant loop are shown in green (1 snapshot every 100 ns, 3 × 1 μs of simulation time). **c**, Top: AlphaFold model of complex between a C-terminally truncated SMO construct (‘7TM’) and the reentrant loop in the pCT, modeled as separate sequences. Bottom: AlphaFold model of near-full-length SMO (including the entire pCT) from the SMO / PKA-C complex, for comparison. The reentrant loop sequence is colored maroon in both models. **d**, Top: overlay of the following GPCR / β-arrestin structures, aligned on the β-arrestin: β1-adrenergic receptor /β-arrestin1 (6TKO), M2AchR / β-arrestin1 (6U1N), neurotensin receptor / β-arrestin1 (6UP7). Bottom: overlay of top 24 AlphaFold models of SMO / PKA-C complex (light blue), along with conf1 (pink) and conf2 (cyan) as shown in Extended Data Fig. 1c.

First, a region of the SMO pCT, termed the reentrant loop (residues 602–609), inserts into a cavity at the intracellular face of the 7TM domain (Fig. 7b). In canonical GPCRs, this cavity opens upon receptor activation to directly engage G proteins^69,70^, GRKs^71,72^, β-arrestins^68,73,74^ or conformation-specific nanobodies^11,75,76^ (Extended Data Fig. 8a). For SMO, in contrast, the cavity engages PKA-C indirectly, through the reentrant loop; binding of the reentrant loop to the cavity is expected to draw the pCT inward, positioning the pseudosubstrate motif and RII-like helix to optimally bind to the PKA-C hinge region and active-site cleft (Fig. 7b). Accordingly, deletion of residues in the reentrant loop strongly inhibited GLI reporter activation (Fig. 7c), without substantially affecting SMO expression, ciliary trafficking or phosphorylation (Extended Data Fig. 5e,f). Although AlphaFold’s pLDDT scores in this region (50–70) are modest, the interaction between the SMO reentrant loop and 7TM cavity remains throughout all-atom MD simulations (Fig. 7d) even in the absence of PKA-C (root-mean-square deviation (r.m.s.d.) values of 4.55 ± 0.99 Å in the PKA-bound state and 5.32 ± 1.51 Å in the apo state; Extended Data Fig. 8b), occurs in SMO–PKA-C complexes from multiple orthologs (Fig. 2c) and persists even when the SMO 7TM domain and reentrant loop are modeled as separate polypeptide sequences (Extended Data Fig. 8c). These results underscore how the SMO 7TM cavity, like those in other GPCRs, enables critical interactions with downstream effectors.

**Extended Data Fig. 9:**
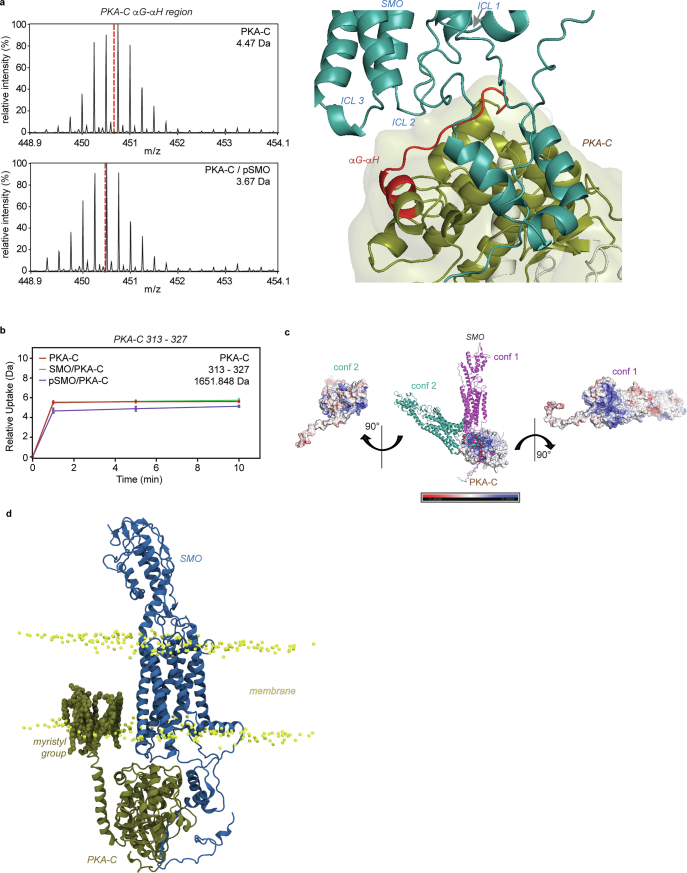
Influence of SMO ICLs and membrane lipids on SMO / PKA-C interactions. **a**, Left: representative HDX-MS mass spectral envelope (at t_ex_ = 5 min) for PKA-C 247–261 in the αG-αH loop. Right: αG-αH sequence protected in the SMO / PKA-C complex mapped onto conf2 of the SMO / PKA-C complex**. b**, Uptake vs. time plots of PKA-C 313–327. **c**, Electrostatic surface potential for each conformation (conf1, conf2) of the SMO / PKA-C complex. Scale is shown at bottom. **d**, Structural snapshots of the PKA-C myristoyl moiety (depicted as brown spheres, 1 snapshot every 300 ns, 3 × 2 μs of simulation time). Membrane phosphate atoms are depicted in yellow.

Second, ICL1 and ICL2 of the SMO 7TM domain engage the surface of PKA-C’s C-lobe, similar to ICL interactions in canonical GPCR complexes with their effectors^68,69,71,73^ (Extended Data Fig. 8d). In GPCR–β-arrestin complexes, the ICLs can bind to several distinct β-arrestin surfaces (Extended Data Fig. 8d), leading to a range of conformations that may enable diverse signaling outcomes^19,20,77,78^. The SMO–PKA-C complex may exhibit similar conformational heterogeneity, as AlphaFold produces two classes of conformations of the SMO–PKA-C complex, with the ICLs engaging distinct C-lobe interfaces (Extended Data Fig. 8d) involving the PKA-C αD–αE and αG–αH loops (Fig. 7b, Extended Data Fig. 9a and Supplementary Discussion 2). These binding interfaces resemble docking sites used by other PKA-C interactors, such as PKA-R’s cyclic nucleotide-binding domains^34,54^ and the cystic fibrosis transmembrane regulator channel^79^; furthermore, the αG–αH loop functions as a hub for protein–protein interactions in many kinases^80^. HDX-MS studies revealed strong protection of αG–αH in the SMO–PKA-C complex compared to PKA-C alone (Extended Data Fig. 9a), supporting the formation of this interface in the SMO–PKA-C complex.

Third, our AlphaFold models suggest roles for lipids in stabilizing SMO–PKA-C interactions, similar to conventional GPCR–effector complexes^68,71,81–86^ (Supplementary Discussion 6). HDX-MS studies revealed protection of a PKA-C C-lobe region (residues 313–327) oriented toward the membrane in the SMO–PKA-C complex (Fig. 7e and Extended Data Fig. 9b), consistent with membrane engagement. These interactions may be electrostatic in nature (Extended Data Figs. 6d and 9c) or may derive from the PKA-C *N*-myristoyl group, which may insert into the membrane as suggested by MD simulations (Extended Data Fig. 9d).

**Extended Data Fig. 10:**
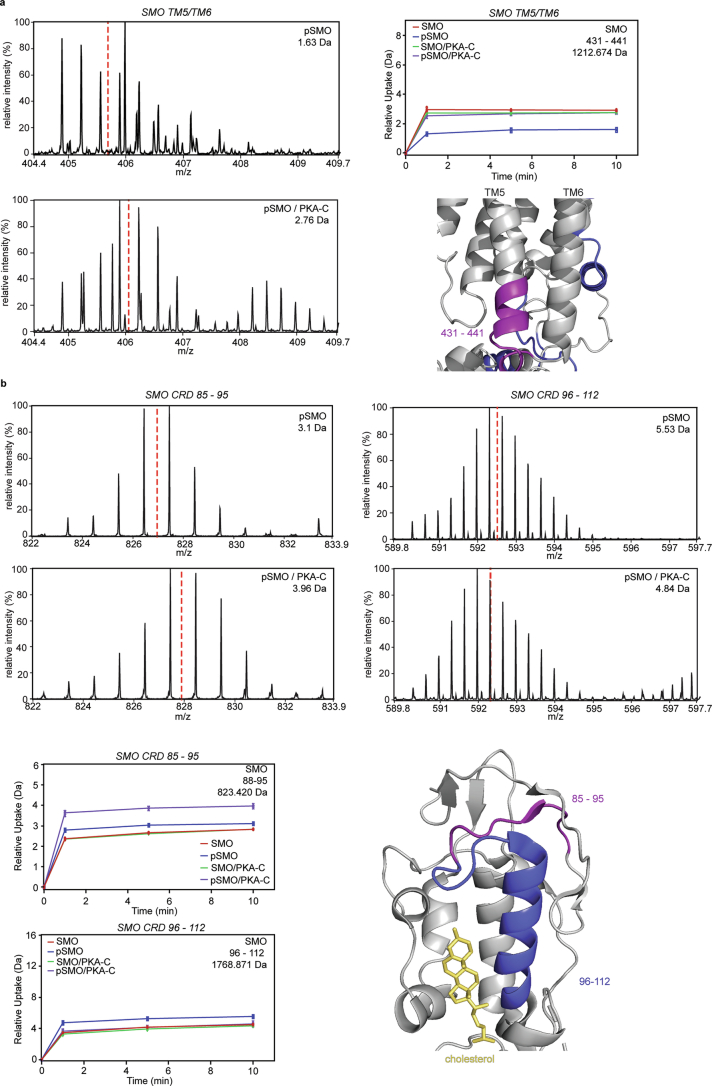
Allosteric changes in SMO induced by PKA-C binding. HDX-MS analysis of the indicated peptide in the TM5-TM6 region (**a**) or CRD (**b**) in phosphorylated SMO (pSMO) alone (top spectrum in each pair), or in complex with PKA-C (bottom spectrum in each pair). In each panel, representative HDX-MS mass spectral envelope and uptake vs. time plots are indicated at left, and regions of SMO that have undergone increased (purple) or decreased (dark blue) deuterium exchange upon SMO / PKA-C interaction are mapped onto the SMO-SAG21k-NbSmo8 structure (PDB: 6O3C)^11^.

Lastly, HDX-MS measurements revealed that PKA-C binding favors conformational changes in several regions of SMO that do not directly contact the kinase, including TM5 and TM6 (Extended Data Fig. 10a), as well as its sterol-binding extracellular cysteine-rich domain (CRD)^6–11^ (Extended Data Fig. 10b). These conformational changes are reminiscent of the classical allosteric interactions described by the ternary complex model of GPCR pharmacology, in which ligand binding influences coupling to cytoplasmic partners and vice versa^87–91^.

Overall, our findings illustrate how the SMO–PKA-C complex, despite an entirely different molecular composition, mimics the protein and lipid interfaces, as well as the allosteric conformational changes, seen in canonical GPCR assemblies with their effectors.

### A model for transmission of Hh signals by the SMO–PKA-C complex

**Fig. 8:**
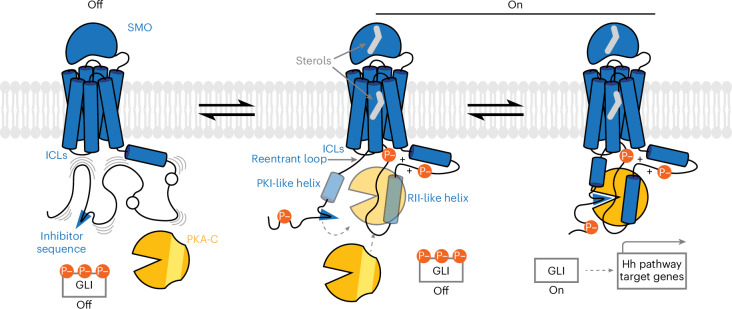
A structural mechanism for Hh signal transduction by the SMO–PKA-C complex. Left, in the Hh pathway off state, SMO (blue) is inactive, with its pCT largely disordered (indicated by wavy lines) and unable to effectively engage PKA-C (orange) because of insufficient phosphorylation by GRK2/3. Middle, in the Hh pathway on state, inhibition of PTCH1 (not shown) enables sterols (gray) to bind the SMO extracellular and 7TM domains, leading to SMO activation and phosphorylation by GRK2/3. This stabilizes secondary structures (PKI-like and RII-like helices) in the pCT and enables SMO to form a complex with PKA-C. PKA-C binding reinforces the SMO pCT secondary structures, further enhancing complex formation. Right, the SMO inhibitor sequence (blue wedge) enters the PKA-C active site to interrupt PKA-C’s catalytic cycle. Consequently, GLI is released from phosphorylation-induced inhibition, leading to the transcription of Hh pathway target genes.

On the basis of our findings, we propose a model for how Hh signals are transduced across the membrane by the SMO–PKA-C complex (Fig. 8).

In the pathway ‘off’ state, SMO is inactive. As a result, the pCT is not efficiently phosphorylated by GRK2/3, remains largely disordered and cannot effectively engage PKA-C (Fig. 8, left).

In the pathway ‘on’ state, Hh proteins bind to and inactivate PTCH1. Consequently, SMO binds membrane sterols^6–12^, favoring an active SMO conformation^11,46^; this enables recognition and phosphorylation by GRK2/3 (Supplementary Discussion 7). SMO phosphorylation stabilizes the pCT through intramolecular electrostatic contacts and interactions with surrounding lipids, enabling formation of secondary structures such as the RII-like helix and the PKI-like helix that mimic PKA-C-interacting regions in classical PKA inhibitors. This nucleates the SMO–PKA-C interaction and binding of PKA-C provides positive feedback to reinforce these structures, similar to the ‘folding upon binding’ that occurs when other IDRs engage their specific targets^32,37,38^. The SMO 7TM cavity stabilizes the SMO–PKA-C complex by bringing the PKI-like and RII-like helices closer together, while the SMO ICLs and membrane lipids provide additional PKA-C-docking surfaces that enhance SMO–PKA-C complex formation (Fig. 8, middle). These interactions cooperate to facilitate insertion of the SMO inhibitor sequence into PKA-C’s active site, which interrupts the enzyme’s catalytic cycle, inhibiting GLI phosphorylation to ultimately elicit transcription of Hh pathway target genes (Fig. 8, right).

Thus, SMO active-state-dependent phosphorylation remodels SMO into a ‘parallel holoenzyme’, analogous to the PKA-R–PKA-C holoenzyme, that blocks PKA-C activity using principles borrowed from canonical GPCR and kinase signaling assemblies.

## Discussion

By merging computational modeling with HDX-MS, biochemical and functional studies, we delineated a structural mechanism for a pivotal step in Hh signal transduction: how active SMO directly binds and inhibits PKA-C to transmit Hh signals intracellularly. These findings deepen our understanding of canonical Hh signaling throughout development and disease and suggest broader principles relevant to GPCR and kinase signaling, as discussed below. Our study was enabled by AlphaFold^49,50,52^, which provided key structural snapshots of SMO’s intrinsically disordered CT in its conditionally folded, PKA-C-bound conformation. The AlphaFold model agrees with our computational and experimental findings and can be docked into our low-resolution cryo-EM map, suggesting that it depicts a physiological state of the complex. While limitations and caveats apply to AlphaFold modeling (Supplementary Discussion 8) and an atomic-resolution experimental structure remains an important goal, our work highlights the power of integrated computational and experimental approaches to probe protein assemblies that have eluded empirical structural approaches. The strategy used here may be more broadly applicable to conditionally folding IDRs, a ubiquitous, essential and poorly understood class of sequences^37–39,92^.

Our work reveals striking parallels between the SMO–PKA-C complex and canonical GPCR and PKA signaling assemblies. Like PKA-R subunits, SMO binds PKA-C by combining a central active-site-binding inhibitor sequence with auxiliary elements contacting peripheral kinase surfaces^2,28,30^. This mimicry is exemplified by the SMO RII-like helix, which, in a remarkable example of evolutionary convergence, uses nearly the same strategy to engage PKA-C as the PKA-RIIβ β4–β5 loop^35^, despite no discernible sequence or secondary-structure homology. In the same vein, the SMO–PKA-C interaction echoes the tripartite architecture of canonical GPCR–β-arrestin complexes, which use the receptor CT, intracellular 7TM surface and membrane lipids^19,20^.

However, a notable distinction between SMO and canonical GPCR signaling complexes lies in how phosphorylation triggers effector binding. Whereas GPCR–β-arrestin complexes rely on insertion of a phosphorylated receptor peptide into a preformed, positively charged β-arrestin groove^68,73,93^, SMO phosphorylation induces a large-scale rearrangement of the SMO CT into a partially ordered state defined by PKA-C-interacting structural elements. This mechanism couples SMO activation to PKA-C binding while also exploiting well-established PKA-C docking motifs^34,35,54^. The GRK2/3-phosphorylated SMO residues may directly stabilize the RII-like helix, as suggested by their proximity to K/R residues in or near this helix. Alternatively, interactions between GRK2/3 phosphorylation sites and K/R residues may facilitate the folding of the SMO pCT by stabilizing key intermediates, similar to other phosphorylated IDRs^37,94,95^.

Beyond defining the SMO–PKA-C complex in structural terms, our work raises several broader questions regarding SMO–GLI communication that can be addressed in future studies, including whether the SMO–PKA-C mechanism is evolutionarily conserved, how pseudosubstrate binding integrates with other regulatory mechanisms of PKA in cells, whether scaffolds contribute to SMO–PKA-C binding in cilia and how SMO activation coordinates additional steps in GLI activation such as phosphorylation by GLI-activating kinases (Supplementary Discussion 9). Blocking the SMO–PKA-C interfaces characterized here may also provide a therapeutic strategy to thwart ectopic Hh signaling in cancers, especially when resistance to SMO orthosteric site inhibitors develops^96,97^. Beyond Hh signaling, our study has general implications for kinases and GPCRs. How PKA-C recognizes full-length physiological substrates remains unclear, as existing structures have largely focused on short peptides^29,53,98,99^. Our study suggests that substrate IDRs, where most kinase phosphorylation motifs reside^100–102^, facilitate substrate recognition by forming transient PKA-C-interacting elements similar to the SMO IDR. The SMO–PKA-C complex also provides a model to study structural interplay between GRK and PKA phosphorylation on GPCRs, a widespread but poorly understood regulatory mechanism^103–105^. Lastly, GRK phosphorylation-induced remodeling of GPCR IDRs may operate broadly throughout the GPCR superfamily to enable interactions with conventional^106^ or unconventional effectors, leading to an expanded array of regulatory complexes, signaling outputs and biological outcomes. Understanding and controlling these processes across GPCRs represent exciting future challenges.

## Methods

### AlphaFold modeling

AlphaFold 2.3.0 modeling was performed with localcolabfold (https://github.com/YoshitakaMo/localcolabfold)^109^ on a Dell Alienware desktop machine with an RTX 4090 GPU of 24 GB. We produced 50 models with ten seeds and all five AlphaFold 2.3.0 weight sets without the use of templates from the Protein Data Bank (PDB). This was performed for various constructs (for example, full-length mouse SMO and various truncations, as well as sequences from different species). A sample command is as follows: colabfold_batch --model-type alphafold2_multimer_v3 --zip --sort-queries-by none --amber --use-gpu-relax --num-seeds 10 --num-recycle 10 --recycle-early-stop-tolerance 1.0 smo_pka.fasta SMO/>SMO.out

Structures were ranked with the default function in Colabfold (0.8 × ipTM + 0.2 × pTM). Structures were visualized in PyMOL (version 2.5; Schrödinger).

AlphaFold3 models were obtained from the AlphaFold3 server (https://golgi.sandbox.google.com/), which was the only form of AlphaFold3 available at the time. For models with PKA-C, two Mg^2+^ ions and ATP were included as ligands. Phosphorylation sites were manually entered using the PTM option on the server. Models were produced of complexes of phosphorylated mouse PKA-C (pT197 and pS338) and phosphorylated mouse SMO (GRK2/3 sites: pS560, pS594, pT597, pS599, pS642, pT644 and pT648), along with non-GRK2/3 sites S578 and S666, all described previously^13^, as well as pS615, identified and described here (Extended Data Fig. 6a). Five random seeds were used (by submitting identical jobs five times) for a total of 25 models for each construct. The models were ranked by pairwise ipTM scores of PKA-C and SMO, with the top-scoring model analyzed and discussed in the Results.

As discussed above, AlphaFold 2.3.0 produced two distinct orientations of the GPCR domain of SMO with respect to the kinase domain of PKA-C, which we designated conf1 and conf2. The top ipTM scores of conf1 and conf2 were 0.84 and 0.80, respectively. AlphaFold3 models most closely resembled conf1 AlphaFold 2.3.0 models. The highest scoring AlphaFold3 model had a chain_pair_ipTM of 0.78 for the SMO–PKA-C interaction.

For clarity, the sequences of the highly disordered mouse SMO N terminus (residues 1–64) and distal CT (residues 675–793) were removed from the structural models in the figure panels.

AlphaFold models discussed in this manuscript are available for download from Zenodo (10.5281/zenodo.13826712)^110^.

### Molecular biology

For SMO protein expression and purification, FLAG-tagged mouse SMO (residues 64–674, with N-terminal HA signal sequence, FLAG tags and TEV protease site) and FLAG–SMOΔCT (as above, but with SMO residues 64–566) in pVLAD6 were described previously^13,15^. For expression and purification of the soluble SMO pCT, we used the pHTSHP vector as previously described^14^. For GLI reporter assays, full-length mouse SMO in the pGEN vector with C-terminal his and Myc tags was described previously^6,13,14^. For ciliary localization studies, FLAG-SMO-nanoluc-IRES-mNG3k/pEF5-FRT-hygro was previously described^15^. N-terminally His-tagged GRK2/pFastBac^111^ and mouse PKA-C in pRSET-b^14^ were previously described. A minimal-cysteine ((−)Cys) PKA-C construct (PKA-C-C199A) was used as the background to introduce the PKA-C G137C substitution. Mutant DNA constructs in the above vectors were prepared in-house by Gibson assembly or commercially (Epoch Life Sciences) and verified by Sanger and/or next-generation sequencing before use.

### Cell culture and transfections

HEK293 suspension cells were cultured and transfected or BacMam-infected as previously described^13–15^. *Smo*^*−/−*^ mouse embryonic fibroblasts (MEFs) were cultured and transiently transfected as previously described^12–14^. NIH3T3 Flp-in cells were cultured and stably transfected by Flp-in integration, as previously described^15^.

### Small molecules, antibodies and other reagents

SAG21k was obtained from BioTechne. Vismodegib was obtained from LC Laboratories (V-4050). KAADcyc was obtained from Toronto Research Chemicals (K171000). Cmpd101 was obtained from Hello Bio (HB2840). Diamide was obtained from Sigma-Aldrich (D3648). BS3 was obtained from BioVision (2327-50). EDC (1-ethyl-3-[3-dimethylaminopropyl]carbodiimide hydrochloride) was obtained from G Biosciences (BC25-5). Sulfo-NHS (*N*-Hydroxysulfosuccinimide, sodium salt) was obtained from Cayman (20680). Control or ShhN conditioned medium was prepared as previously described^112^. The following antibodies were used in this study: mouse anti-FLAG M2 (Sigma, F3165), rat anti-Arl13b (BiCell Scientific, 90413), rabbit anti-acetylated tubulin (Enzo Life Sciences, BML-SA452-0100), rabbit anti-pSMO^15^ (7TM Antibodies, 7TM0239A) and mouse anti-Myc (clone 4A6, Millipore 05-724). Alexa-conjugated (Thermo Fisher) or horseradish-peroxidase-conjugated (Promega) secondary antibodies were used for immunofluorescence or western blotting detection, respectively.

### Protein purification and in vitro phosphorylation

To obtained pSMO, FLAG–SMO was expressed in HEK293 Freestyle or HEK293 GnTI^−^ cells, along with GRK2–eGFP, using the BacMam approach in the presence of 10 mM sodium butyrate and 1 µM SAG21k and purified by FLAG affinity chromatography and gel-filtration chromatography, followed by in vitro GRK2 phosphorylation as previously described^15^. To obtain nonphosphorylated SMO, we followed the same procedure except that (1) SMO inverse agonist vismodegib was used in place of SMO agonist SAG21k during expression and purification and (2) the GRK2 coexpression and GRK2 in vitro phosphorylation steps were omitted, as previously described^15^. The soluble SMO pCT was expressed and purified from *Escherichia* *coli* by sequential Ni-NTA affinity, SUMO tag cleavage, reverse Ni-NTA, cation exchange and gel filtration as previously described^14^, with the following modifications: (1) *E*. *coli* was grown in LB rather than TB; (2) expression was induced by adding IPTG and incubating the cells at 30 °C for 5 h; and (3) the SUMO tag cleavage and overnight 4 °C dialysis steps were combined. MSP1E3D1 scaffold protein was expressed and purified as previously described^113^.

His-tagged GRK2 was expressed in High Five cells by baculovirus and purified by Ni-NTA affinity chromatography and gel-filtration chromatography as previously described^15,111^.

WT, nonmyristoylated mouse PKA-C was used for HDX-MS and disulfide-trapping studies; it was expressed in *E*. *coli* and purified by IP20 chromatography as previously described^14,114^. SPR studies used myristoylated PKA-C, which was prepared by coexpression with yeast *N*-myristoyltransferase in *E*. *coli* BL21(DE3) cells as described previously^115^. A K7C substitution was introduced into PKA-C to increase myristoylation efficiency in *E*. *coli*, as described previously^116,117^, and myristoylated PKA-C was purified by IP20 chromatography. To confirm myristoylation, IP20 purified PKA-C was run over a MonoS column and eluted protein samples were dialyzed into buffer containing 20 mM KH_2_PO_4_ pH 6.5, 50 mM KCl and 1 mM TCEP and submitted to the Molecular MS Facility at University of California, San Diego. Intact protein analysis was performed by using an Agilent 6230 time-of-flight MS (TOFMS) instrument coupled with an Agilent 1260 liquid chromatography (LC) system. The Jet Stream electrospray ionization source was operated under positive ion mode with the following parameters: capillary voltage, 3,500 V; fragmentor voltage, 175 V; drying gas temperature, 325 °C; sheath gas temperature, 325 °C; drying gas flow rate, 10 L min^−1^; sheath gas flow rate, 10 L min^−1^; nebulizer pressure, 40 psi. The chromatographic separation was performed at room temperature on a Phenomenex Aeris wide-pore C-4 column (inner diameter: 2.1 mm; length: 50 mm; particle size: 3.6 µm). Mobile phase A was high-performance LC (HPLC)-grade water with 0.1% TFA and mobile phase B was HPLC-grade acetonitrile with 0.1% TFA. The mobile phase was delivered at a rate of 0.3 ml min^−1^ under gradient conditions as follows: increase from 5% mobile phase B to 90% mobile phase B in 12 min, hold at 90% mobile phase B for 2 min, return to 5% mobile phase B in 1 min and equilibration with 5% mobile phase B for 7 min. Agilent MassHunter software was used for data acquisition and analysis.

### SPR studies

For SPR studies, SMO was reconstituted into biotinylated MSP1E3D1 nanodiscs using a minor modification of previously published procedures^113^, summarized below. His-tagged MSP1E3D1 scaffold protein biotinylated as previously described^113^ except that a 1:10 molar ratio of MSPE3D1 to NHS-biotin was used and the extent of biotinylation was assessed by monitoring capture of biotinylated MSPE3D1 on streptavidin magnetic beads (Thermo Fisher, 88816). SMO proteins were subjected to in vitro phosphorylation and purification as previously described^15^, except that we eliminated the gel-filtration step following FLAG purification of the GRK2 phosphorylation reaction and proceeded directly to nanodisc reconstitution. Nanodiscs were assembled using a 1:10:857:2,571 molar ratio of SMO, MSP1E3D1, lipid and cholate, respectively, in 1× HEPES–NaCl–EDTA (HNE) buffer. A lipid mixture consisting of 92 mol% 3:2 POPC and POPG and 8 mol% cholesterol (dissolved in chloroform) was placed in a borosilicate tube and dried under a stream of N_2_ gas to eliminate chloroform, followed by a 1-h vacuum desiccation at room temperature. Subsequently, sodium cholate was introduced to the lipid mixture, followed by bath sonication for 10 min. Upon addition of water, an additional 5 min of sonication was performed to ensure complete lipid dissolution. Then, 20× HNE buffer (400 mM HEPES, 2000 mM NaCl and 20 mM EDTA), MSP1E3D1 protein and SMO were added to the lipid mixture and incubated for 3 h at 4 °C. Detergent removal was achieved by adding Bio-Beads (Bio-Rad) at a concentration of 14.5 mg per 100-μl reaction volume, followed by overnight rotation at 4 °C. The reconstituted SMO was supplemented with 5 mM CaCl_2_ and applied to an M1 FLAG affinity column (to remove excess MSP1E3D1 and GRK2 leftover from the in vitro phosphorylation step). The sample was washed with 20 mM HEPES pH 7.5, 100 mM NaCl and 5 mM CaCl_2_ and then eluted using a buffer containing 20 mM HEPES pH 7.5, 100 mM NaCl, 1 mM EDTA and 0.2 mg ml^−1^ FLAG peptide.

SPR interaction studies were performed in running buffer (20 mM MOPS pH 7.0, 150 mM NaCl, 100 μM EDTA, 1 mM ATP, 10 mM MgCl_2_ and 0.001% P20 surfactant) at 25 °C using Biacore 3000 instrument (GE Healthcare). Measurements were performed using a Biotin CAPture Kit (Cytiva) to capture biotinylated SMO nanodiscs according to the manufacturer’s instructions. Briefly, Biotin CAPture reagent was immobilized to a level of 3,000 response units (RU) on the sensor chip CAP at the beginning of each cycle, followed by the sequential capture of respective SMO nanodiscs (WT, ΔCT, 5KE and IFR→AAA) with a capture level of 250–350 RU on separate flow cells (flow rate: 10 μl min^−1^). Serial dilutions of myr-PKA-Cα-K7C (16 nM–1 μM) were diluted in running buffer and injected with increasing concentrations at a flow rate of 10 μl min^−1^ for 60 s (association) followed by 60 s of dissociation in running buffer without PKA-Cα. Data were corrected (double referencing) for nonspecific binding and buffer effects by subtracting SPR signals from a flow cell with only CAPture reagent, as well as blank runs by injecting buffer without the analyte, using Biacore 3000 Evaluation Software 4.1.1 (Cytiva). The sensor chip was regenerated by three sequential injections of 6 M guanidinium chloride and 0.25 M NaOH to remove the CAPture reagent until the baseline level was reached. Steady-state analysis was performed with GraphPad Prism 8.0.1 (GraphPad Software).

### Analytical-scale SMO–PKA-C disulfide-trapping studies

Phosphorylated or nonphosphorylated forms of WT or mutant FLAG–SMO were prepared as described above and gel-filtered into 20 mM HEPES pH 7.5, 150 mM NaCl, 0.025% GDN and 1 μM SAG21K. PKA-C proteins were buffer-exchanged into this same buffer using Zeba 7k desalting columns. To accurately compare disulfide-trapping efficiencies between WT and mutant proteins, we quantified each protein’s concentration by A_280_ (adjusted for the extinction coefficient) and then subjected each sample to a twofold set of serial dilutions, which were analyzed by stain-free imaging of SDS–PAGE gels; the concentrations of each protein were then manually adjusted as necessary to ensure uniform concentrations between each set of WT and mutant proteins. For the reaction setup, 4 μl of SMO (12.5 μM), 4 μl of PKA (6.25 μM), 1 μl of 10x reaction buffer (10 μM SAG21K, 10 mM ATP and 100 mM MgCl_2_) and 1 μl of 50 mM NaOH (to adjust reaction pH to 8.0), except for Extended Data Fig. 5b, in which [PKA-C] was titrated to the concentrations indicated in the figure panel. Samples were then incubated for 30 min at room temperature to promote SMO–PKA-C complex formation. Following the initial incubation, diamide (prepared fresh before use) was added to each reaction at a final concentration of 5 mM to initiate disulfide bond formation and samples were incubated for 1 h at room temperature. Subsequently, 10 μl of 2× Laemmli sample buffer (without reducing agent) was added to each sample, which were then loaded onto a 4–20% SDS–PAGE gel. Disulfide-trapped quantification was completed by analyzing adjusted, background-subtracted band intensities using Fiji. For each disulfide-trapping condition, the adjusted intensity of the disulfide-bonded product was normalized by dividing it by the sum of the adjusted intensities of both the disulfide-bonded product and free SMO.

A similar protocol was used for the disulfide trapping of soluble SMO pCT L637C with human PKA-C, with some modifications. Briefly, both SMO pCT L637C and human PKA-C (WT or C343S) were buffer-exchanged into 20 mM HEPES pH 8 and 150 mM NaCl following gel filtration. During complex formation, 10 mM ATP and 100 mM MgCl_2_ were added and the final concentrations of SMO pCT and PKA-C were 40 μM and 20 μM, respectively. The mixture was incubated for 3 h following the addition of 5 mM diamide and analyzed by SDS–PAGE as described above for the near-full-length FLAG–SMO experiments.

### CD studies

SMO pCT (residues 565–657) was purified as described previously^14^. Peak fractions from gel filtration containing intact SMO pCT were pooled and subjected to dialysis. Dialysis was performed using tubing with a 3.5-kDa molecular weight cutoff in 1× PBS (pH 7.4) at 4 °C for two overnight cycles. After dialysis, the protein concentration was adjusted to 40 μM. CD measurements were conducted using an AVIV model 410 CD spectrometer. Samples were analyzed in a quartz cuvette (path length: 1 mm) at 25 °C, recording data every 2 nm across a wavelength range of 200–260 nm with a 3-s averaging time. Each condition, including a blanking control, was measured in quintuplicate. The collected raw data were averaged and blank-subtracted before normalization to mean residue molar ellipticity ([*θ*] = 100 × θ/(*C* × *l* × *n*), where *C* is the concentration of protein (mM), *l* is the path length (cm) and *n* is the number of peptide bonds in the protein) using a custom Python script. Data points with dynode voltages exceeding 500 V were excluded (specifically at a wavelength of 200 nm).

### Preparative scale SMO–PKA-C disulfide trapping for HDX-MS

Large-scale pSMO-L637C–PKA-C disulfide trapping for HDX-MS followed a similar procedure as described in the preceding section, except that SMO and PKA-C were present at 12 μM and 24 μM, respectively, the reaction was scaled up to 0.5 ml and the diamide-induced disulfide bond formation proceeded for 2 h. The reaction was then subjected to gel-filtration chromatography on a Superdex 200 Increase 10/300 GL column equilibrated in 20 mM HEPES pH 8, 50 mM NaCl, 0.01% GDN, 1 μM SAG21k, 10 mM MgCl_2_ and 1 mM ATP. Fractions were analyzed by nonreducing SDS–PAGE and those containing the pSMO–PKA-C complex were pooled and concentrated. The same procedure was used for disulfide trapping of nonphosphorylated SMO-L637C–PKA-C; note that, to obtain nonphosphorylated SMO samples, we took the purification steps described above except that vismodegib was used in place of SAG21k, GRK2 was not coexpressed with SMO and purified SMO was not subjected to in vitro GRK2 phosphorylation.

### GLI reporter assays

GLI reporter assays on *Smo*^*−/−*^ MEFs transiently transfected with WT or mutant constructs, along with 8×Gli-Firefly and SV40-*Renilla* dual luciferase reporter plasmids, were performed as previously described^12,13^. GLI reporter assay data were normalized to total SMO expression, which we quantified by measuring the expression of each Myc-tagged SMO mutant in whole-cell lysates using anti-Myc immunoblotting (Supplementary Fig. 5).

### Monitoring phosphorylation of WT or mutant SMO by immunoblotting

HEK293 Freestyle cells were cultured in Freestyle 293 expression medium supplemented with 1% FBS, as previously described^12–15^. For transfection, 3 ml of HEK293 Freestyle cell culture (density: 3.0–4.0 million cells per ml) was transferred to a six-well plate and incubated with 5 mM sodium butyrate at 37 °C in a 5% CO_2_ atmosphere for 15 min. Transfection complexes were prepared by combining 1.25 μg of SMO WT or mutant DNA with 1.25 μg of GRK2–GFP DNA in 250 μl of Opti-MEM I reduced-serum medium. The mixture was vortexed, followed by the addition of 7.5 μl of TransIT-293 reagent. After vortexing for 15 s, the mixture was incubated at 25 °C for 15 min. The transfection complex was then added dropwise to the cell culture and incubated in a shaking incubator at 37 °C and 5% CO_2_ for 48 h. Cells were treated with either SAG21k or SAG21k + Cmpd101 for 4 h, harvested by centrifugation at 2,000*g* for 10 min, flash-frozen in liquid nitrogen and stored at −80 °C.

Cell pellets were resuspended in 500 μl of SDS-free RIPA buffer (50 mM Tris pH 7.5, 150 mM NaCl, 2% NP40, 0.25% sodium deoxycholate and 10% glycerol) supplemented with Pierce protease and phosphatase inhibitor tablet. The samples were homogenized by pipetting and incubated at 4 °C for 1 h with rotation. Lysates were clarified by centrifugation at 21,100*g* for 10 min. An input sample was separated and the remaining supernatant was incubated with 10 μl of ChromoTek Myc-Trap magnetic agarose at 4 °C for 1 h with rotation. The resin was washed three times with 1 ml of SDS-free RIPA buffer. Proteins were eluted by adding 50 μl of 2× Laemmli sample buffer containing β-mercaptoethanol and incubating at 25 °C for 5 min.

Input and eluted samples were separated by SDS–PAGE using Criterion stain-free gels, transferred to PVDF membranes, blocked with 5% milk and probed overnight with either rabbit anti-pSMO (7TM Antibodies, 7TM0239A) or mouse anti-Myc (clone 4A6, Millipore 05-724). The relevant secondary antibodies were applied for 2 h, followed by chemiluminescence detection using the ChemiDoc system (Bio-Rad).

### SMO ciliary localization studies

Ciliary localization studies on FLAG-tagged SMO stably expressed in NIH3T3 Flp-in cells were formed as previously described^15^. Briefly, stably transfected cells were grown to confluency on glass coverslips, switched to low-serum medium (DMEM + 0.5% FBS + penicillin, streptomycin and glutamine) overnight to induce ciliation and then treated for 4 h to induce SMO ciliary accumulation. Cells were washed, fixed in PFA, permeabilized with 0.1% Triton X-100 and blocked overnight in Tris-buffered saline and Tween-20 supplemented with 2% BSA. Cells were stained with anti-FLAG to identify stably expressed SMO and anti-Arl13b and anti-acetylated tubulin to mark cilia, followed by appropriate secondary antibodies + DAPI counterstain. Coverslips were mounted onto a slide with SlowFade mounting medium. Images were acquired on a Leica SP8 laser-scanning confocal microscope using a ×40 water-immersion lens. Identical zoom factors, exposure times and gain settings were used in all experiments. The SMO signal in cilia was quantified, background-subtracted and graphed. Data for each condition represent 100 cilia counted from two or more separate fields in two independent trials.

### MD simulations

Systems for simulations were generated using CHARMM-GUI^118–120^ with parameters for the protein component assigned from the CHARMM36m force field^121^ and other components assigned from the CHARMM36 force field^122^. All systems were solvated using TIP3P water, with the net charge kept at 0 with a 0.15 M concentration of NaCl ions. All simulation were carried out using the ACEMD engine^123^.

#### Simulations of SMO–PKA-C AlphaFold models

The AlphaFold models of the SMO–PKA-C complex (conf1) were embedded in a membrane consisting of POPC and POPG (3:2) and 8% cholesterol, in line with the composition of the SMO-containing nanodiscs used in this study and previously^12^. The receptor was oriented using data from the OPM database^124^. Initial internal waters were approximated using the homolwat server^125^. On the SMO C-tail, the GRK2/3 phosphorlyation sites, defined here (Extended Data Fig. 6) and in our previous study^13^, were included in the model (phosphorylated S560, S578, S594, T597, S599, S615, S642, T644, T648 and S666), while, on PKA-C, residues S10, S197 and T338 were phosphorylated, as these residues are known to be phosphorylated in PKA-C purified from recombinant systems^126^ and G2 was N-terminally myristoylated, as PKA-C is uniformly cotranslationally myristoylated in eukaryotes.

The placement of ligands was carried out using previous crystallographic data (active-state SMO structure for cholesterol (PDB 6O3C), PKA-C–IP20 structure for ATP + Mg ions (PDB 1ATP)). Protonation of titratable residues was assigned using the Protonate 3D module available in the MOE package (version 2015.10; https://www.chemcomp.com/). Afterward, histidine protonation states were visually inspected and, in cases of uncertainty, we compared both protonation states by performing a quick minimization using MOE (Amber 10:EHT force field, Born solvation model with default electrostatic interaction cutoffs) to check whether one state promoted more interactions. The generated systems were equilibrated (3 × 400 ns), with constraints applied to protein backbone using a timestep of 4 fs. Pressure was kept at 1.0132 using the Monte Carlo barostat. This was followed by three production runs of 2 µs in NVT conditions, which were respawned from each individual NPT run.

#### Simulations of apo SMO AlphaFold models

To simulate the reentrant loop stability in the apo SMO model (without PKA-C), we followed the same building protocol as described above for the SMO–PKA-C complex. To facilitate comparison, we maintained the same protonation states as in the previous system, except that PKA-C was not included. We carried out three production runs of 1 µs in NVT conditions, which were respawned from each individual NPT run. During the NVT run, temperature was maintained at 310 K using a Langevin thermostat with a damping coefficient of 0.1 ps^−1^.

#### Simulations of SMO PKI-like and RII-like helices and their mutants

To assess the impact of mutations on the SMO PKI-like helix and RII-like helix, we simulated the region of the SMO C-tail spanning from residue D562 to R633, using the AlphaFold3 model of the SMO–PKA-C complex as a starting point. The system underwent NPT equilibration (50 ns with constant pressure of 1.0132 and constraints applied to protein backbone). The NPT time was shorter in comparison with the simulations of the full complex, as we only simulated a small protein fragment in a water box excluding any cell membrane environment. Subsequently, each system was simulated in NVT conditions for 500 ns in three replicates.

#### MD analysis

To assess model stability, we aligned studied simulations using either the transmembrane helical backbone of SMO or the helical backbone of the PKA-C domain. After aligning, the conformation and stability of specific elements were studied using the r.m.s.d. ± s.d. as calculated in the VMD package. For contact analysis of phosphorylated residues, we used the getcontacts package (http://getcontacts.github.io) with default values. As polar contacts, we quantified hydrogen bonds, salt bridges and water bridges (maintained by a maximum of one water molecule). To study potential contacts between positively charged lipid headgroups and pSMO residues in proximity of the membrane (residues 594, 597 and 599), we quantified the fraction of frames in which the N atom of at least one POPC headgroup was within 4 Å of at least one of the phosphorylated atoms of the studied residues. The number of frames was measured using an in-house script.

All MD simulations are available from the GPCRmd platform (https://www.gpcrmd.org/dynadb/publications/1537/)^127,128^.

### HDX-MS sample preparation and data acquisition

Because of the instability of the SMO–PKA-C complex during gel-filtration chromatography, described above, we used disulfide trapping to stabilize the complex. The high specificity of this crosslink (C199 of PKA-C to residue 637 of SMO (L637C) (Fig. 3b) enabled us to trap the complex without altering the biological activity. The deuterium exchange reaction was carried out by diluting 3 μl of sample at ~15–20 μM in 57 μl of deuterium exchange buffer (94.99% D_2_O, 20 mM HEPES pH 8.0, 150 mM NaCl, 0.025% GDN, 1 mM ATP and 10 mM MgCl_2_) for a final deuteration of 89.99%. Phosphorylated or nonphosphorylated SMO-L637C–PKA-C complexes were prepared as described above. HDX-MS runs carried out for pSMO were performed in the presence of agonist SAG21k (1 μM) while runs with nonphosphorylated SMO were performed in the presence of inverse agonist KAAD cyclopamine (1 μM), to maintain SMO in an active or inactive conformation. To maintain SMO solubility, the buffer was also supplemented with 0.025% GDN (a nonionic detergent that is MS compatible). The reactions were performed for free PKA-C, free pSMO, free SMO, pSMO–PKA-C complex and SMO–PKA-C complex. The reactions were carried out at 25 °C in triplicate for deuteration times of 1 min, 5 min and 10 min, after which they were quenched by the addition of 60 μl of quench buffer (1.5 M guanidinium hydrochloride and 0.25 M TCEP) to bring the reaction to pH 2.5. Undeuterated control runs were also performed in a buffer without D_2_O.

Next, 100 μl of quenched samples were injected into an Acquity nano-UPLC (ultra-HPLC) HDX manager (Waters) and proteolyzed in an immobilized BEH pepsin column in 0.1% formic acid running at a continuous flow rate of 100 μl min^−1^ to generate peptic peptides. The peptides were trapped in a VanGuard trap column (Waters) and loaded into a Acquity BEH C18 RPLC column (Waters) and eluted in an acetonitrile gradient (8–40%) in 0.1% formic acid, after which the peptides were ionized by electrospray ionization and sprayed into a Synapt XS Quadrupole TOFMS instrument (Waters), where data were acquired in HDMS^E^ mode. Ion mobility settings including a wave velocity of 600 m s^−1^ and transfer wave velocity of 197 m s^−1^ were used with collision energies of 4 V and 2 V for trap and transfer, respectively. Increasing high collision energy from 20–45 V with a cone voltage of 20 V was used to scan an *m*/*z* range of 50–2,000 *m*/*z* in positive ion mode. [Glu^1^]-fibrinogen peptide B (100 fmol min^−1^) at a flow rate of 5 μl min^−1^ was used as a lockspray reference. The entire run time was 15 min, consisting of a 3 min of proteolysis and 12 min of acquisition.

### HDX-MS data analysis

The mouse sequences of SMO (residues 64–674) (UniProt P56726) and PKA-C (UniProt P05132) were used for peptide identification in Protein Lynx Global server (PLGS; version 3.0, Waters) in HDMS^E^ mode with workflow parameters set to nonspecific proteolytic cleavage and variable phosphorylation modifiers at serine, threonine and tyrosine. The PLGS results were combined with the raw spectra for analysis in DynamX version 3.0. The DynamX filters were as follows: minimum intensity = 2,000, minimum products per amino acid = 0.2, minimum peptide length = 5, maximum peptide length = 25 and maximum mass tolerance = 10 ppm. Spectra were analyzed for each state by comparing the undertreated run to the time-point runs and state-wise comparisons were also performed. The final overall sequence coverage on SMO was 56.7%, yielding 72 peptides at an amino acid redundancy of 2.12. Correspondingly, the sequence coverage on PKA-C was 86%, yielding 76 peptides at an amino acid redundancy of 2.56. More than 50% of all peptides in our dataset showed HDX values ≥ 1 Da, indicating efficient proton–deuterium exchange. Backward exchange was estimated to be 19.8% for the most deuterated peptide on pSMO residues 541–546 by considering the deuteration percentage and the number of exchangeable amides from the fully deuterated data at 48 h but no correction was applied to the data. We designated 0.5 Da as the threshold value above which differences in HDX values between experimental conditions (either positive or negative differences) were considered meaningful; this value was established on the basis of experimental uncertainty in deuterium uptake across several proteins at a 98% confidence interval, as defined previously^129^.

Peptides displaying bimodal spectra were selected for bimodal analysis in HXexpress3 (refs. ^130,131^), with four peptides from SMO being chosen for the analysis (residues 96–112, 142–151, 431–441 and 641–654). However, because of poor signal-to-noise ratios, peptides 96–112 and 431–441 were excluded from the analysis. Bimodal analysis was performed for peptides 142–151 and 641–654 and bimodal spectra were assessed for statistical significance using a two-parameter metric: a *P* value < 0.02 and a confidence interval > 98% in the regression metric. Two additional parameters were also evaluated to prevent overfitting: the Δ*χ* metric and separation metric, as described previously^132^.

Deuteros (version 2.0) was used for significance testing of HDX-MS data^133^. A hybrid significance test (*P* < 0.02) was performed to determine statically significant deuterium exchange differences across different states^134^. For each comparison, data were plotted on a Woods plot^135^. All HDX-MS data were deposited to ProteomeXchange (PXD067578).

### Mapping SMO phosphorylation sites using targeted MS

FLAG-tagged mouse SMO (residues 64–674) was purified from HEK293 cells treated with SMO modulators and/or GRK2/3 inhibitors, as described previously^13^. The purified protein extracts from different conditions were denatured and reduced in 1.7 M urea, 50 mM Tris-HCl pH 8.0 and 1 mM DTT at 37 °C for 30 min and alkylated in the dark with 3 mM iodoacetamide at room temperature for 45 min; excess iodoacetamide was quenched with 3 mM DTT for 10 min at room temperature. For digestion, proteins were incubated with 1 μg of chymotrypsin at 37 °C overnight. To stop the digestion, samples were acidified with 0.5% trifluoroacetic acid (TFA). Digested samples were desalted for MS analysis using a BioPureSPE mini 96-well plate (20 mg PROTO 300 C18; The Nest Group) according to standard protocols.

Digested samples were analyzed on an Orbitrap Exploris 480 MS system (Thermo Fisher Scientific) equipped with an Easy nLC 1200 UPLC system (Thermo Fisher Scientific) interfaced through a Nanospray Flex nanoelectrospray source. For all analyses, samples were loaded onto a C18 reverse-phase column (inner diameter: 75 μm) packed with 25 cm of ReprosilPur 1.9-μm 120 Å particles (Dr. Maisch). Mobile phase A consisted of 0.1% FA and mobile phase B consisted of 0.1% FA and 80% acetonitrile. Peptides were separated by an organic gradient from 2% to 28% mobile phase B over 32 min, followed by an increase to 44% B over 19 min and a hold at 90% B for 9 min, at a flow rate of 300 nl min^−1^. Analytical columns were equilibrated with 6 μl of mobile phase A. To build a spectral library, the four biological replicates for each condition were pooled and acquired in a data-dependent manner. Data-dependent analysis was performed by acquiring a full MS1 scan over the *m*/*z* range of 350–1,250 in the Orbitrap at 120,000 resolution (200 m/z) with a normalized automatic gain control (AGC) target of 100%, a radiofrequency (RF) lens setting of 40% and a maximum ion injection time set to ‘auto’. Dynamic exclusion was set to 30 s, with a 10-ppm exclusion width setting. Peptides with charge states of 2–6 were selected for MS/MS interrogation using higher-energy collisional dissociation (HCD), with a set cycle time of 1 s. MS/MS scans were analyzed in the Orbitrap using isolation width of 1.3 *m*/*z*, normalized HCD collision energy of 30%, normalized AGC of 200% at a resolution of 15,000 and a maximum ion injection time set to auto. For all acquisitions, QCloud was used to control instrument longitudinal performance during the project^136^. All proteomic data were searched against the human UniProt database (UniProt-reviewed sequences downloaded July 2018) augmented with the sequence of the affinity-tagged mouse SMO. Peptide and protein identification searches, as well as label-free quantitation, were performed using the MaxQuant data analysis algorithm (version 1.6.12.0)^137^ using the above-described parameters. The database search results were used to generate a spectral library in Skyline (version 20.2.0.343)^138^ and to extract optimal coordinates for targeted proteomics assays (so-called parallel reaction monitoring (PRM) assays). PRM measurements were performed on all four biological replicates per condition separately using the above-described gradient for spectral library generation but operating the Orbitrap Exploris 480 in PRM mode. Targeted MS2 spectra were acquired using the following parameters: 60,000 resolution, scan range set to auto, normalized HCD collision energy of 30%, RF lens setting of 50%, an AGC target set to ‘standard’, the maximum injection time set to ‘dynamic’, desired minimum points across the peak set to 9 and an isolation window of 1.2 *m*/*z*. Selected SMO peptides were targeted in 3-min-wide transition windows. The resulting data were analyzed with Skyline (version 20.2.0.343)^138^ for identification and quantification of peptides. MSstats was used for statistical analysis^139^. Statistical analysis of unmodified SMO and detected phosphosites was performed separately for the different digestion conditions using the statistical framework Msstats^139^. Intensities were estimated using the sample quantification function in MSstats, which provides model-based estimation of phosphosite and protein abundance combining individual peptide intensities. Quantification was graphed using Prism 8 (GraphPad). Raw data and PRM transition files can be accessed and queried online (https://panoramaweb.org/SMO_phospho_2024.url)^140^.

### Purification of PKA-C from native tissue for cryo-EM studies

Native-source PKA-C was purified from a beef heart by IP20 chromatography. A fresh beef heart (Tooele Valley Meats) was transported on ice before the purification of PKA-C. Meat from the left ventricle of the beef heart was cut into small pieces. For 100 g of tissue, 200 ml of lysis buffer (50 mM Tris-HCl pH 7.4, 50 mM NaCl and 2 mM MgCl_2_) supplemented with Pierce EDTA-free protease inhibitor tablets was added. Then, the mixture was homogenized in a blender for five 1-min cycles with cooling intervals between cycles. The homogenized material was centrifuged at 13,000*g* and 4 °C for 20 min. The supernatant was centrifuged for a second round at 38,000*g* and 4 °C for 45 min. The supernatant was filtered through an empty resin column before batch binding with IP20 resin in 50-ml conical tubes. After supplementing with 100 µM cAMP, 5 mM MgCl_2_ and 3 mM ATP, the tubes were incubated at 4 °C overnight with gentle end-to-end rotation. The IP20 resin was separated by passing the mixture through a column. The resin was washed with W1 buffer (50 mM Tris-HCl pH 7.4, 50 mM NaCl, 2 mM MgCl_2_ and 0.4 mM ATP) and W2 buffer (50 mM Tris-HCl pH 7.4, 250 mM NaCl, 2 mM MgCl_2_ and 0.4 mM ATP). Bound protein was eluted with elution buffer (50 mM Tris-HCl pH 7.4, 200 mM arginine, 50 mM NaCl and 1 mM EDTA). The protein sample was concentrated and further purified using a Superdex 200 Increase 10/300 GL column equilibrated with storage buffer (20 mM MOPS pH 7.0, 150 mM NaCl and 2 mM β-mercaptoethanol). Monomeric fractions were pooled, concentrated, flash-frozen and stored at −80 °C before use.

### Preparation of SMO–PKA-C complex samples for cryo-EM studies

Six SMO–PKA-C complex samples were prepared for cryo-EM studies. Size-exclusion chromatography and SDS–PAGE profiles are provided in Supplementary Data File 3.

To obtain the SMO–PKA-C sample before grid preparation, purified and in vitro phosphorylated mouse SMO (residues 64–674) and native-source bPKA-C were mixed in binding buffer (20 mM HEPES pH 7.5, 150 mM NaCl, 0.01% GDN, 10 mM MgCl_2_, 1 mM ATP and 1 µM SAG21k) at a 1:1.2 molar ratio and incubated at room temperature for 20 min. Then the mixture was concentrated to approximately 18 mg ml^−1^ for cryo-EM studies.

To prepare the SMO–PKA-C sample in MSP1E3D1 nanodiscs, purified and in vitro phosphorylated mouse SMO (residues 64–674) was reconstituted into MSP1E3D1 nanodiscs with a lipid mixture of 92 mol% 3:2 POPC and POPG and 8 mol% cholesterol. Reconstituted SMO was purified by M1 anti-FLAG antibody-conjugated resin and mixed with native-source bPKA-C in a detergent-free binding buffer (20 mM HEPES pH 7.5, 150 mM NaCl, 10 mM MgCl_2_, 1 mM ATP and 1 µM SAG21k) at a 1:1.7 molar ratio and incubated at room temperature for 1.5 h. The sample was subjected to size-exclusion chromatography using a Superdex 200 Increase 10/300 GL column equilibrated with the detergent-free binding buffer. The peak fractions of monomeric complex were pooled and concentrated to 4.4 mg ml^−1^ for cryo-EM studies.

For the disulfide-trapped SMO-L637C–PKA-C complex, purified and in vitro phosphorylated mouse SMO-L637C (residues 64–674) and native-source bPKA-C were mixed in disulfide-trapping buffer (20 mM HEPES pH 8.0, 150 mM NaCl, 0.01% GDN, 10 mM MgCl_2_, 1 mM ATP and 1 µM SAG21k) at a 1:2 molar ratio and incubated on ice for 30 min. Then, diamide was added into the mixture at a final concentration of 5 mM and the sample was further incubated at room temperature for 1.5 h. The sample was subjected to size-exclusion chromatography using a Superdex 200 Increase 10/300 GL column equilibrated with the disulfide-trapping buffer. The peak fractions of monomeric complex were pooled and concentrated to 11.2 mg ml^−1^ for cryo-EM studies.

For the BS3-crosslinked SMO–PKA-C complex, purified and in vitro phosphorylated mouse SMO (residues 64–674) and native-source bPKA-C were mixed in binding buffer (20 mM HEPES pH 7.5, 150 mM NaCl, 0.01% GDN, 10 mM MgCl_2_, 1 mM ATP and 1 µM SAG21k) at a 1:1.2 molar ratio and incubated at room temperature for 30 min. Fresh BS3 dissolved in binding buffer was added to a final concentration of 1 mM and the sample was incubated at room temperature for 45 min. After incubation, the reaction was stopped by the addition of Tris-HCl pH 8.0 to a final concentration of 50 mM. Then, the complex was purified using a Superdex 200 Increase 10/300 GL column equilibrated with binding buffer. The peak fractions of monomeric complex were pooled and concentrated to 10 mg ml^−1^ for cryo-EM studies.

For the EDC/sulfo-NHS-crosslinked SMO–PKA-C complex, purified and in vitro phosphorylated mouse SMO (residues 64–674) and native-source bPKA-C were mixed in binding buffer (20 mM HEPES pH 7.5, 150 mM NaCl, 0.01% GDN, 10 mM MgCl_2_, 1 mM ATP and 1 µM SAG21k) at a 1:1.35 molar ratio and incubated at room temperature for 15 min. Then freshly made EDC/sulfo-NHS solution was added to the mixture to a final concentration of 7.5 mM/15 mM and the sample was further incubated at room temperature for 45 min. The sample was subjected to size-exclusion chromatography using a Superdex 200 Increase 10/300 GL column equilibrated with the binding buffer. The peak fractions of monomeric complex were pooled and concentrated to 11 mg ml^−1^ for cryo-EM studies.

For the SMO–PKA-C complex subjected to dual EDC/sulfo-NHS and BS3 crosslinking, purified and in vitro phosphorylated mouse SMO (residues 64–674) and native-source bPKA-C were first crosslinked with EDC/sulfo-NHS and purified by size-exclusion chromatography as described above. The peak fractions of monomeric complex from were pooled and concentrated. Then, BS3 was added to the concentrated sample at a final concentration of 2 mM and the sample was incubated at room temperature for 45 min. Then the reaction was stopped by addition of 50 mM Tris-HCl pH 8.0 and the complex was purified by size-exclusion chromatography. The peak fractions of monomeric complex were pooled and concentrated to 10 mg ml^−1^ for cryo-EM studies.

### Cryo-EM grid preparation, data acquisition and processing

SMO–PKA-C complex samples were frozen on glow-discharged UltrAuFoil grids (Quantifoil, R 1.2/1.3, 300-mesh) using a Vitrobot Mark IV (Thermo Fisher Scientific). Data were collected on a 300-kV Titan Krios transmission EM instrument as 40-frame videos with a total exposure of 50 e^−^ per Å^2^. The raw pixel size was 1.06 Å. Procedures for cryo-EM data processing are provided in Supplementary Data File 3 and statistics for data collection and processing are provided in Supplementary Table 2. Generally, cryo-EM videos were imported into cryoSPARC and motion-corrected with patch motion correction. Contrast transfer function (CTF) parameters were estimated by Patch CTF estimation or CTFFIND4. Micrographs with CTF-fit resolution better than 3.5 Å or 4 Å were selected for particle picking. Blob picker was used to generate two-dimensional (2D) templates using a small subset of micrographs. Then, these 2D templates were used by template picker to pick particles from the whole dataset. Particles were cleaned by iterative 2D classification. Particles from good 2D classes (2× binned, with a pixel size of 2.12 Å) were subjected to ab initio reconstruction. The best three-dimensional (3D) class was further classified by running a new ab initio reconstruction job. For each dataset, one 3D class was selected from the ab initio reconstruction jobs and used to generate an EM map through nonuniform refinement. Although the nominal resolutions are reported at 5–9 Å, the reconstructed maps lack the expected level of detail. This discrepancy indicates severe particle heterogeneity, which likely caused misalignment and overfitting during the global refinement. Therefore, each of these maps represent a low-resolution consensus envelope of multiple conformational states, rather than a true high-resolution structure.

The EM maps for (1) the SMO–PKA-C sample mixed before grid preparation; (2) the SMO–PKA-C sample in MSP1E3D1 nanodiscs; (3) the disulfide-trapped SMO-L637C–PKA-C complex; (4) the BS3-crosslinked SMO–PKA-C complex; (5) the EDC/sulfo-NHS-crosslinked SMO–PKA-C complex; and (6) the SMO–PKA-C complex subjected to dual EDC/sulfo-NHS and BS3 crosslinking were deposited to the EM Data Bank (EMDB) under accession codes EMD-74330, EMD-74331, EMD-74332, EMD-72508, EMD-74333 and EMD-74334, respectively.

### Statistics and reproducibility

All statistics used GraphPad Prism (version 10.6.1) unless stated otherwise, using *t*-tests or analyses of variance, as indicated in Supplementary Table 4. No statistical method was used to predetermine sample size. However, we used similar numbers of cells and animals to previous studies in our field that applied these experimental models. All experiments shown were reproduced on at least two independent occasions. For all statistical analyses, *n* reflects the number of independent experimental or biological replicates. No data were excluded from the analyses. Randomization and blinding methods were not used.

### Reporting summary

Further information on research design is available in the Nature Portfolio Reporting Summary linked to this article.

## Online content

Any methods, additional references, Nature Portfolio reporting summaries, source data, extended data, supplementary information, acknowledgements, peer review information; details of author contributions and competing interests; and statements of data and code availability are available at 10.1038/s41594-026-01800-z.

## Supplementary information

Supplementary Information Supplementary Tables 1–4, Figs. 1–6 and Discussions 1–9.

Reporting Summary

Supplementary Data File 1 AlphaFold 2.3 models of SMO–PKA-C complex.

Supplementary Data File 2 AlphaFold 3 models of SMO–PKA-C complex.

Supplementary Data File 3 Cryo-EM sample preparation and data processing.

Supplementary Data File 4 Complete list of HDX-MS peptides and uptake values for both SMO and PKA-C.

## Source data

Source Data Fig. 3 Unprocessed SDS–PAGE gel image.

Source Data Fig. 4 Unprocessed SDS–PAGE gel image.

Source Data Fig. 5 GLI reporter and SPR unprocessed data.

Source Data Fig. 6 GLI reporter unprocessed data and unprocessed SDS–PAGE gel image.

Source Data Fig. 6 Unprocessed quantification of disulfide-trapping data.

Source Data Fig. 7 GLI reporter unprocessed data.

Source Data Extended Data Fig. 1 CD unprocessed data.

Source Data Extended Data Fig. 3 Unprocessed SDS–PAGE gel image.

Source Data Extended Data Fig. 5 Unprocessed SDS–PAGE gel and western blot images.

Source Data Extended Data Fig. 5 GLI reporter unprocessed data.

Source Data Extended Data Fig. 6 MS, GLI reporter and MD unprocessed data.

Source Data Extended Data Fig. 7 Unprocessed SDS–PAGE gel image.

Source Data Extended Data Fig. 7 Unprocessed quantification of disulfide-trapping data.

## Data Availability

AlphaFold models of the SMO–PKA-C complexes were deposited to Zenodo (10.5281/zenodo.13826712)^110^. The MD simulation trajectories were deposited to GPCRmd (https://www.gpcrmd.org/dynadb/publications/1537/). MS data were deposited to the Panorama server (phosphoproteomics; PXD055272) or ProteomeXchange (HDX-MS; PXD067578). Cryo-EM maps were deposited to EMDB (EMD-74330, EMD-74331, EMD-74332, EMD-72508, EMD-74333 and EMD-74334). All other data supporting the findings in this study are available within the paper and its Supplementary Information. All data and unique biological materials are available upon request from the authors. Source data are provided with this paper.

## Code availability

Custom code was used for CD data processing and can be accessed from Zenodo (10.5281/zenodo.18970396)^141^.
