# Supplementary material for: Structural mechanism for noncanonical GPCR signaling in the Hedgehog pathway
Source: Nat Struct Mol Biol. 2026 Apr 30;33(5):795–809. doi: 10.1038/s41594-026-01800-z (PMC13186710; doi:10.1038/s41594-026-01800-z)
Supplement: Supplementary file 1 — Supplementary Tables 1–4, Figs. 1–6 and Discussions 1–9. [file 41594_2026_1800_MOESM1_ESM.pdf]

nature structural & molecular biology

Supplementary information https://doi.org/10.1038/s41594-026-01800-z

Structural mechanism for noncanonical GPCR signaling in the Hedgehog pathway

In the format provided by the authors and unedited

Supplementary Table 1. Molecular dynamics simulation parameters.

Simulated System	Number of Runs	Runtime	Number of Atoms	Dimensions (Angstroms)
SMO/PKA-C complex conf 1	3	2 μs	253,308	119 x 119 x 191
SMO/PKA-C complex conf 2	3	2 μs	216,350	118 x 118 x 195
SMO conf 1 without PKA-C	3	1 μs	216,355	119 x 119 x 164
SMO PKI-like and RII-like helices - WT	3	500 ns	41,218	90 x 90 x 90
SMO PKI-like and RII-like helices - V626D	3	500 ns	41,192	90 x 90 x 90
SMO PKI-like and RII-like helices - V626D, V630D	3	500 ns	41,217	90 x 90 x 90

Supplementary Table 2: Cryo-EM data collection, refinement and validation statistics

	SMO / PKA-C mixed immediately prior to grid preparation (EMD-74330)	SMO / PKA-C complex in MSP1E3D1 nanodiscs (EMD-74331)	Disulfide-trapped SMO-L637C / PKA-C complex (EMD-74332)
**Data collection and processing**			
Magnification	81,000	81,000	81,000
Voltage (kV)	300	300	300
Electron exposure (e–/Å^2)	50	50	50
Defocus range (μm)	-1 to -3.5	-1 to -3.5	-1 to -3.5
Pixel size (Å)	2.12	2.12	2.12
Symmetry imposed	C1	C1	C1
Initial particle images (no.)	282,081	6,122,660	6,805,032
Final particle images (no.)	27,222	43,052	212,082
Map resolution (Å)	6.35	8.57	5.41
FSC threshold	0.143	0.143	0.143
Map resolution range (Å)	6.0-15.7	7.5-18.5	5.0-12.7

	BS3-crosslinked SMO / PKA-C complex (EMD-72508)	EDC/Sulfo-NHS-crosslinked SMO / PKA-C complex (EMD-74333)	SMO / PKA-C complex subjected to dual EDC/Sulfo-NHS and BS3 crosslinking (EMD-74334)
**Data collection and processing**			
Magnification	81,000	81,000	81,000
Voltage (kV)	300	300	300
Electron exposure (e–/Å^2)	50	50	50
Defocus range (μm)	-1 to -3.5	-1 to -3.5	-1 to -3.5
Pixel size (Å)	2.12	2.12	2.12
Symmetry imposed	C1	C1	C1
Initial particle images (no.)	483,280	9,040,330	10,866,369
Final particle images (no.)	21,122	330,512	265,795
Map resolution (Å)	8.59	5.08	5.30
FSC threshold	0.143	0.143	0.143
Map resolution range (Å)	8.4-14.9	4.7-14.0	4.9-15.4

Supplementary Table 3. HDX-MS reaction details and parameters.

Protein side	SMO Side				PKA-C Side		
Dataset	pSMO	pSMO/ PKA-C	SMO	SMO/P KA-C	PKA-C	PKA- C/pSM O	PKA- C/SMO
HDX reaction details	3 µL sample (15-20 µM) + 57 µL deuterium exchange buffer (94.99% D2O, 20 mM HEPES pH 8.0, 150 mM NaCl, 0.025% Glycodiosgenin, 1 mM ATP, 10 mM $MgCl_2$). Final deuteration – 89.99%. All deuterium exchange carried out at 25°C.						
	Reactions quenched with 60 µL with 1.5 M GdnHCl and 0.25 M TCEP to bring pH to 2.5 at ~0°C						
	Runs for pSMO carried out in presence of SMO agonist SAG21K (1 µM)<br>Runs for SMO carried out in presence of inverse agonist KAAD cyclopamine (1 µM)						
HDX time course	0, 1, 5, 10 min						
Replicates	3 Technical Replicates, 1 Biological Replicate						
Number of peptides	72				76		
Sequence coverage	56.70%				86.00%		
Peptide redundancy	2.12				2.91		
Significant difference threshold	p-value<0.02 (Hybrid significance testing in Deuteros v2.0)						
Back exchange	19.80%						

Supplementary Table 4. Tests of statistical significance[a] for all figures.

Figure	Condition 1	Condition 2	Significant?	p-value
5A	SMO: WT	SMO: I573A	yes	0.002076
5A	SMO: WT	SMO: F577A	yes	0.008238
5A	SMO: WT	SMO: IFR → AAA	yes	0.00009
5A	SMO: WT	SMO: del570-581	yes	0.000126
5B	SMO: WT	SMO: del570-581	yes	0.000319
5B	SMO: WT	SMO: V262D	yes	0.000428
5B	SMO: WT	SMO: V626D, V630D	yes	0.001418
5B	SMO: WT	SMO: K575P	yes	0.03195
5E	SMO: WT	SMO: IFR → AAA	yes	0.0047 [b]
5E	SMO: WT	SMO: 5KE	yes	0.0025 [b]
6C	SMO: WT	SMO: 5KE	yes	9.03E-04
6E	pSMO-S570C + PKA-C-(-)cys-G136C	WT-pSMO + PKA-C-(-)cys	yes	<0.0001 [b]
6E	pSMO-S570C + PKA-C-(-)cys-G136C	WT-pSMO + PKA-C-(-)cys-G136C	yes	<0.0001 [b]
6E	pSMO-S570C + PKA-C-(-)cys-G136C	pSMO-S570C + PKA-C-(-)cys	yes	<0.0001 [b]
6E	pSMO-S570C + PKA-C-(-)cys-G136C	SMO-S570C + PKA-C-(-)cys	yes	0.0012 [b]
6E	pSMO-S570C + PKA-C-(-)cys-G136C	SMO-S570C + PKA-C-(-)cys-G136C	yes	0.0002 [b]
7C	SMO: WT	SMO: del602-609	yes	0.001933
ED5C	SMO: WT	SMO: I573A	yes	0.039581
ED5C	SMO: WT	SMO: F577A	yes	0.040831
ED5C	SMO: WT	SMO: IFR → AAA	yes	0.029263
ED5C	SMO: WT	SMO: K575P	yes	0.014941
ED5C	SMO: WT	SMO: V626D	yes	0.028441
ED5C	SMO: WT	SMO: V626D, V630D	yes	0.021876
ED5C	SMO: WT	SMO: K571E, K575E, K579E	yes	0.030256
ED5C	SMO: WT	SMO: K565E, R566E	yes	0.044755
ED5C	SMO: WT	SMO: K568E, K569E	yes	0.012035
ED5C	SMO: WT	SMO: 5KE	yes	0.030968
ED5F	SMO: delCT	SMO: WT	yes	<0.0001 [c]
ED5F	SMO: delCT	SMO: 5KE	yes	<0.0001 [c]
ED5F	SMO: delCT	SMO: IFR → AAA	yes	<0.0001 [c]

ED5F	SMO: delCT	SMO: K575P	yes	<0.0001 [c]
ED5F	SMO: delCT	SMO: V626D, V630D	yes	<0.0001 [c]
ED5F	SMO: delCT	SMO: 602-609Ala	yes	<0.0001 [c]
ED5F	SMO: delCT	SMO: del602-609	yes	<0.0001 [c]
ED5F	SMO: WT	SMO: 5KE	yes	<0.0001 [c]
ED5F	SMO: WT	SMO: IFR → AAA	yes	0.0222 [c]
ED5F	SMO: WT	SMO: K575P	no	0.1108 [c]
ED5F	SMO: WT	SMO: V626D, V630D	no	0.7653 [c]
ED5F	SMO: WT	SMO: 602-609Ala	no	0.2221 [c]
ED5F	SMO: WT	SMO: del602-609	yes	<0.0001 [c]
ED6A	Phospho-S615: Vehicle	Phospho-S615: KAADcyc	yes	4.60E-07 [d]
ED6A	Phospho-S615: SAG21k	Phospho-S615: SAG21k + Cmpd101	yes	2.62E-07 [d]
ED6A	Total SMO: Vehicle	Total SMO: KAADcyc	no	0.0631729 [d]
ED6A	Total SMO: SAG21k	Total SMO: SAG21k + Cmpd101	no	0.16327519 [d]
ED6E	SMO: WT	SMO: del570-581	yes	0.006875
ED6E	SMO: WT	SMO: K565E, R566E, K568E, K569E	yes	0.006843
ED6E	SMO: WT	SMO: K565E, R566E	yes	0.006512
ED6E	SMO: WT	SMO: K568E, K569E	yes	0.003091
ED6E	SMO: WT	SMO: K571E, K575E, K579E	yes	0.006729
ED7D	pSMO-A574C + PKA-C-(-)cys-G136C	WT-pSMO + PKA-C-(-)cys	yes	<0.0001 [b]
ED7D	pSMO-A574C + PKA-C-(-)cys-G136C	WT-pSMO + PKA-C-(-)cys-G136C	yes	<0.0001 [b]
ED7D	pSMO-A574C + PKA-C-(-)cys-G136C	pSMO-A574C + PKA-C-(-)cys	yes	0.0039 [b]
ED7D	pSMO-A574C + PKA-C-(-)cys-G136C	SMO-A574C + PKA-C-(-)cys	yes	<0.0001 [b]
ED7D	pSMO-A574C + PKA-C-(-)cys-G136C	SMO-A574C + PKA-C-(-)cys-G136C	yes	<0.0001 [b]

[a] All tests of statistical significance were performed as an unpaired Welch's t-test (two-sided) with multiple comparisons corrected using the Holm-Šídák method unless noted otherwise in the table.

[b] Statistical significance was assessed using an unpaired t-test (two-tailed).

[c] Statistical significance was assessed using an unpaired Mann-Whitney test (two tailed).
[d] Statistical significance was assessed using a Student's t-test with Benjamini-Hochberg correction.

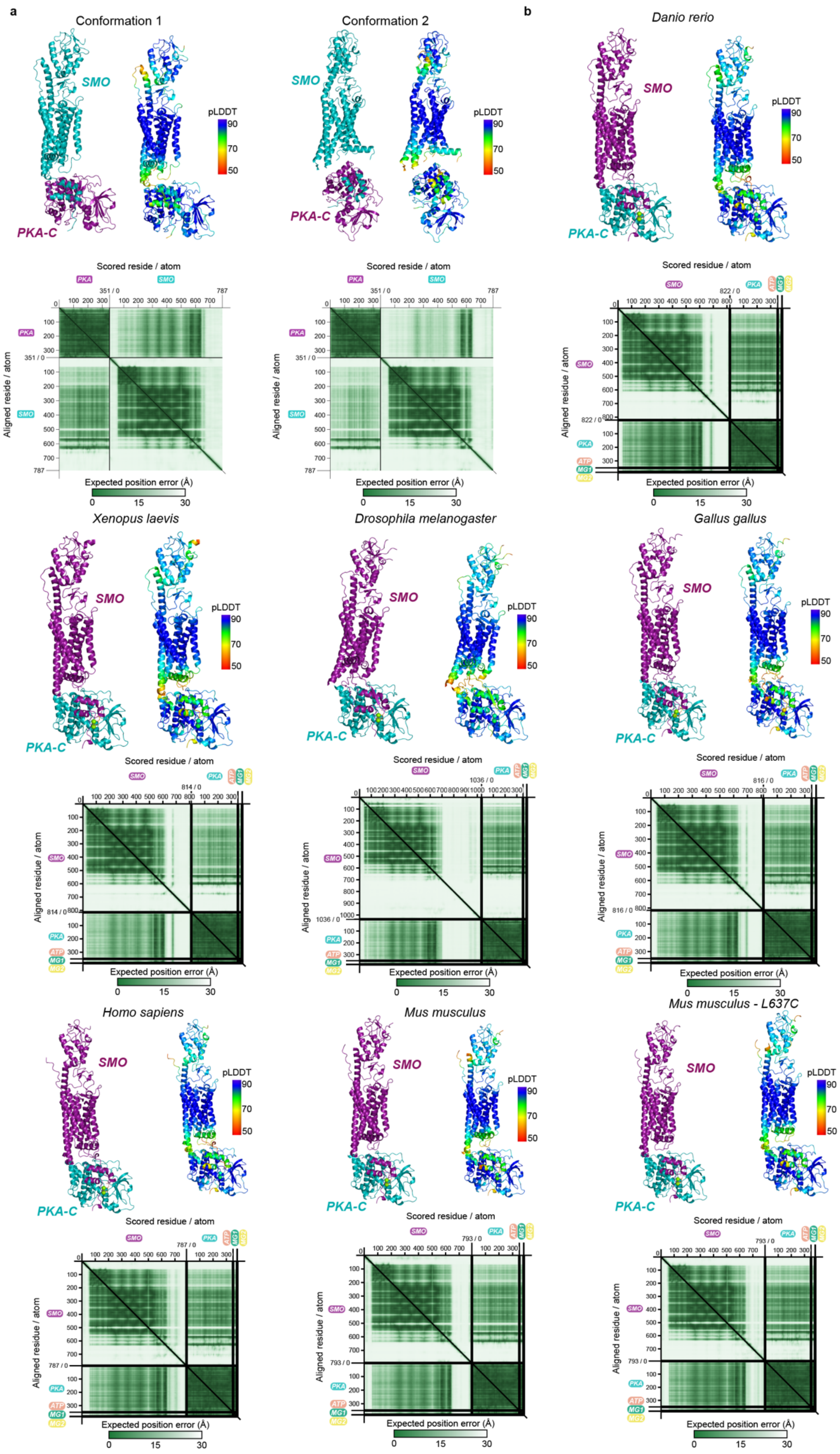

Supplementary Figure 1. **AlphaFold models and confidence metrics across species. a,** AlphaFold 2.3 model, pLDDT overlay, and PAE plot for the mouse SMO/PKA-C complex conformation 1 (left) and conformation 2 (right). **b,** Same as in **a**, using AlphaFold 3 across various species or constructs.

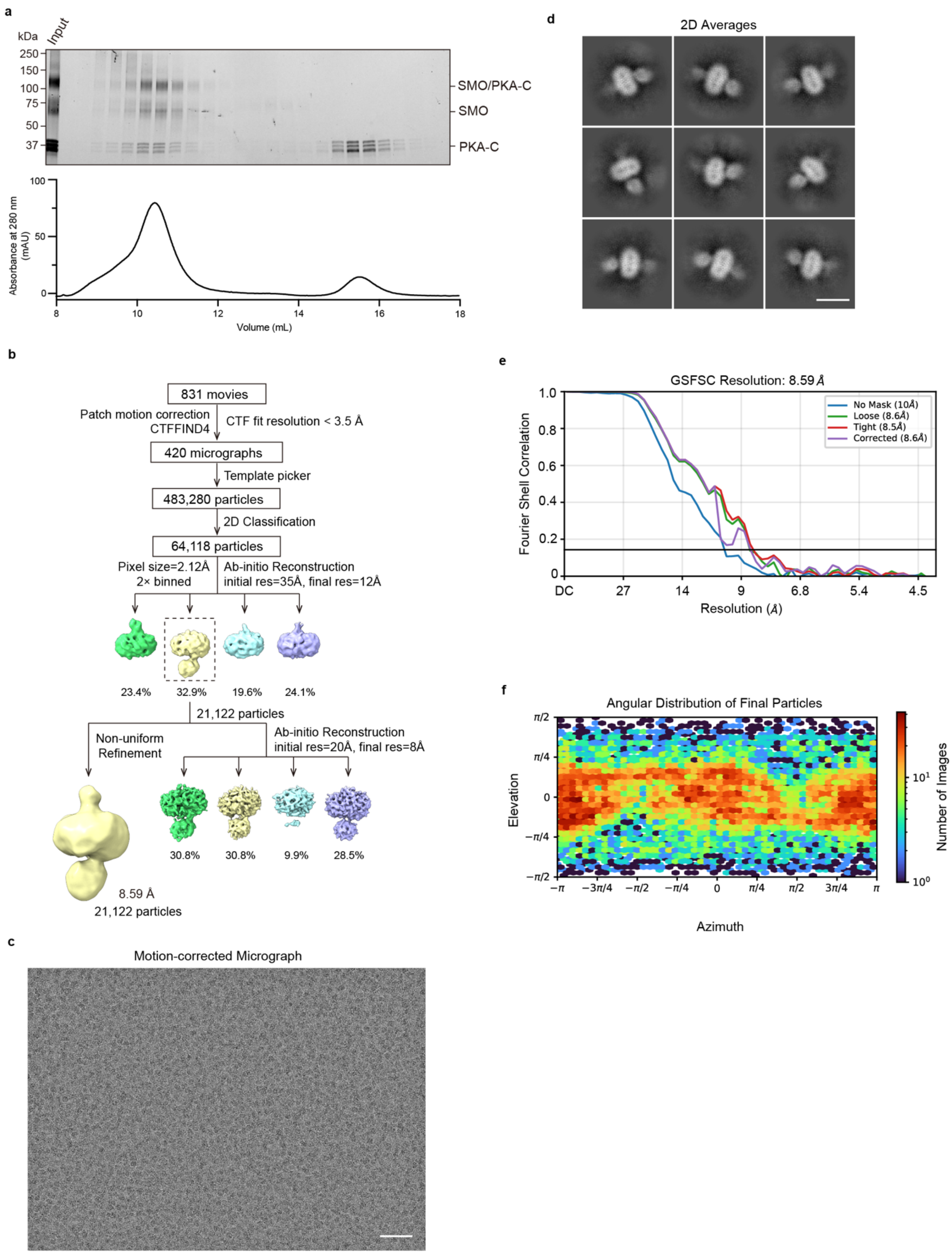

Supplementary Figure 2. **SMO/PKA-C complex preparation and cryoEM data processing. a,** Size-exclusion chromatography and SDS-PAGE analysis of BS3-crosslinked SMO/PKA-C complex. **b,** Workflow of cryoEM data processing. **c,** Representative cryoEM micrograph (scale bar: 50 nm). **d,** Representative 2D class averages (scale bar: 10 nm). **e,** Gold-standard Fourier shell correlation (FSC) curves of the EM map. **f,** Angular distribution plot of final particles.

a

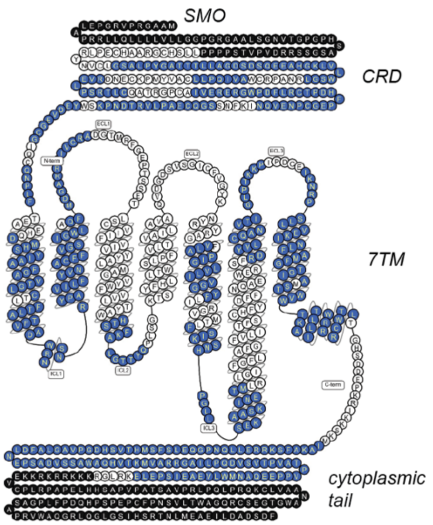

b

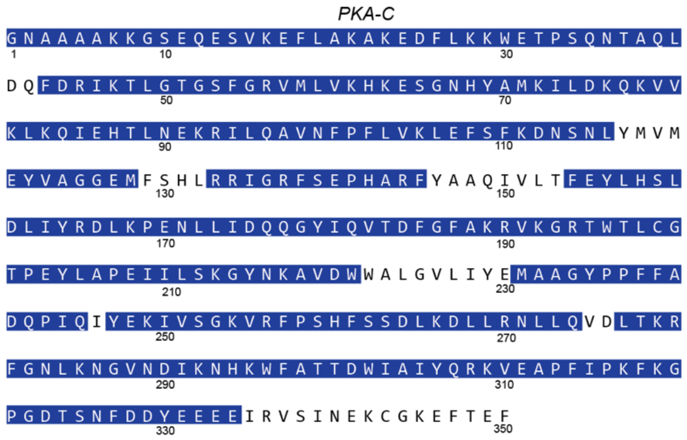

c

Runs	SMO (L637C)	PKA-C	SMO Phosphorylation Status	Protein Analyzed
1	x		phosphorylated	SMO
2	x		non-phosphorylated	
3	x	x	phosphorylated	
4	x	x	non-phosphorylated	
5		x	phosphorylated	PKA-C
6		x	non-phosphorylated	
7	x	x	phosphorylated	
8	x	x	non-phosphorylated	

d

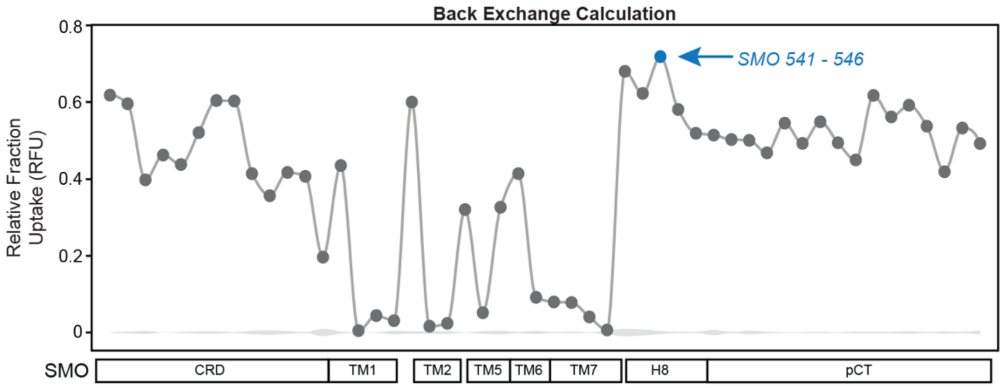

Supplementary Figure 3: SMO and PKA-C sequence coverage in HDX-MS, and summary statistics of HDX-MS runs. a, Left: HDX-MS sequence coverage of the SMO L637C construct presented as a “snake plot”. Residues covered by the MS measurements are colored blue, while residues that were not detected by MS are colored white. **b,** HDX-MS sequence coverage of PKA-C. Colors are as in (**a**). **c,** Summary of protein coverage, # of peptides, and redundancy for MS runs. **d,** list of MS runs presented in this study (see Methods for details on sample preparation). **e,** Back-exchange for SMO 541-546, the most extensively deuterated peptide in our data set, was measured at 19.8% (see Methods).

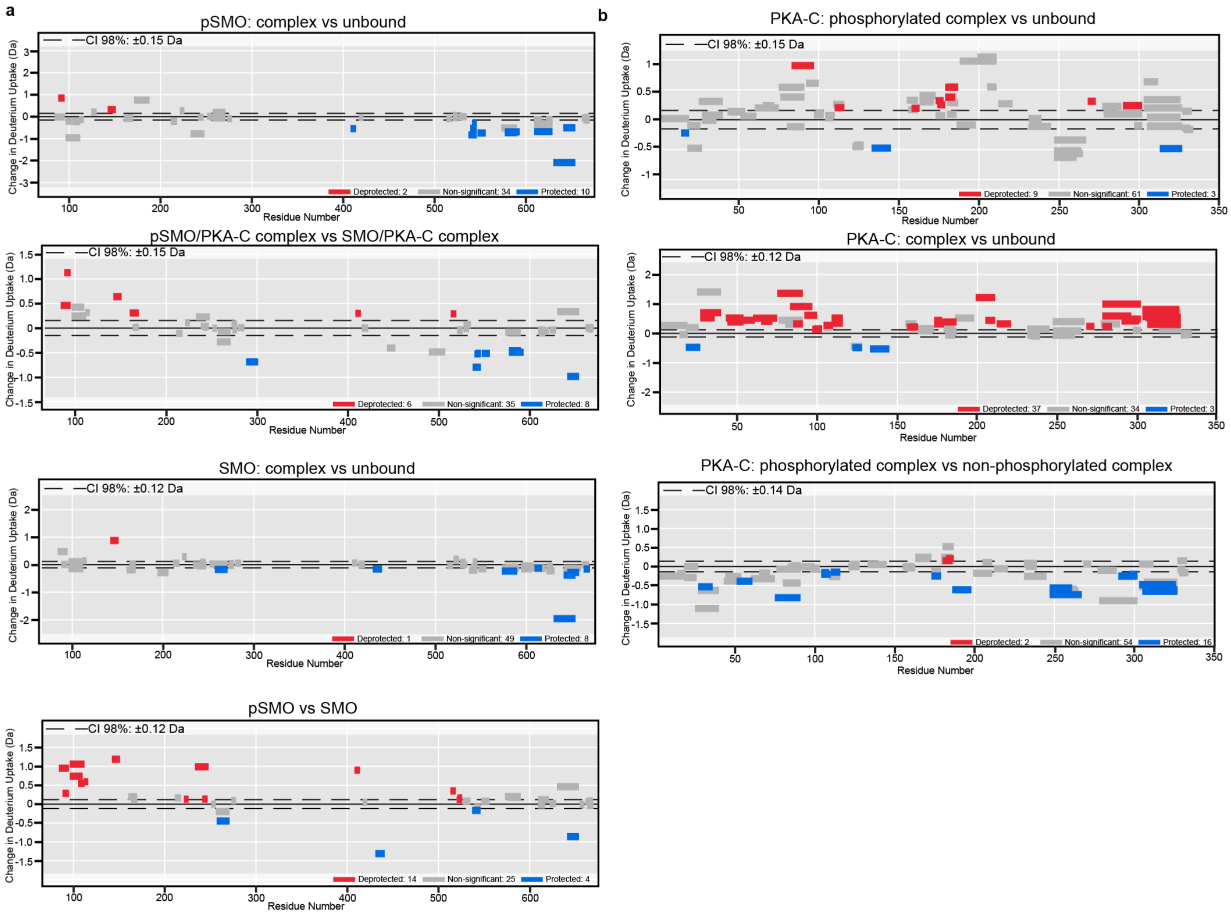

Supplementary Figure 4: Statistical analysis of HDX-MS data. Woods plots for peptides in SMO (a) and PKA-C (b), showing residue number (X-axis), change in deuterium uptake (Y-axis), and 98% confidence interval (dashed lines). Statistically significant protection and deprotection are indicated in blue and red, respectively. “Complex” denotes the indicated protein within the SMO/PKA-C complex (with pSMO or nonphosphorylated SMO, as indicated), and “unbound” denotes the apo protein.

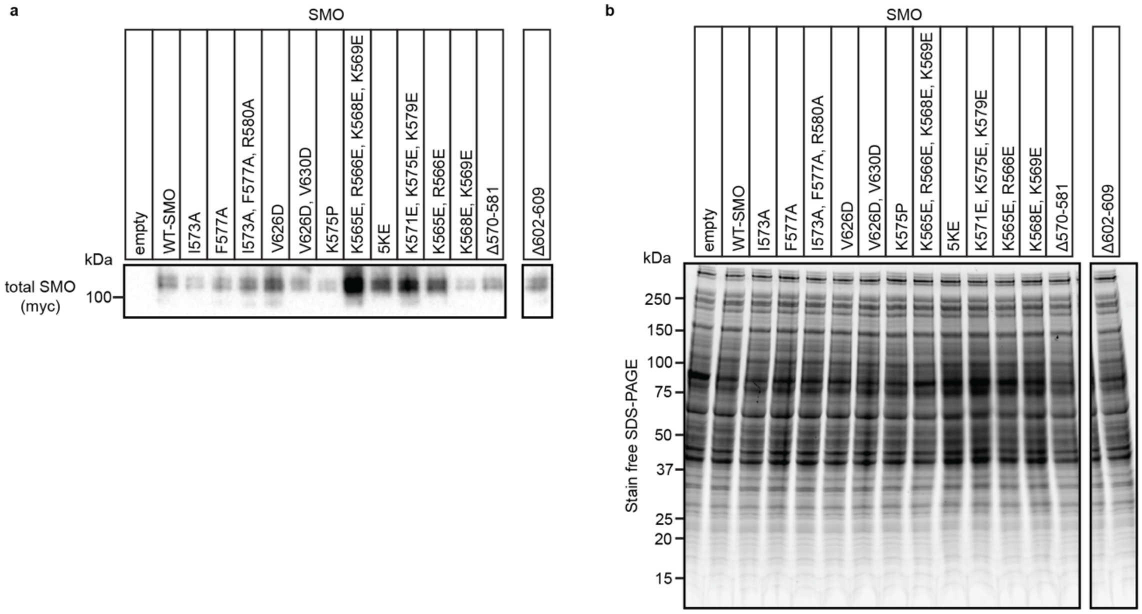

Supplementary Figure 5. a, Expression levels of wild-type and mutant myc-tagged SMO constructs in lysates from transfected HEK293 cells, assessed by anti-myc immunoblotting (see Methods). **b,** Stain-free imaging of total protein, shown as a loading control for the blot.

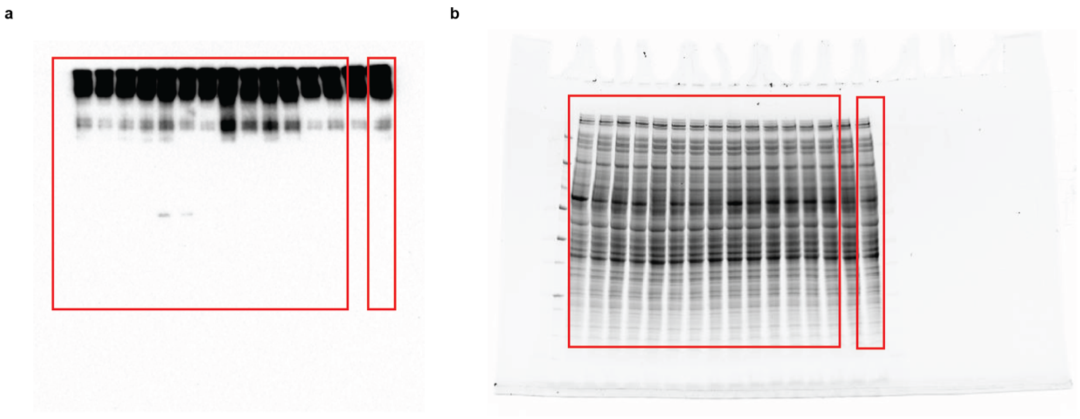

Supplementary Figure 6. Uncropped Western-blot **(a)** and SDS-PAGE gel **(b)** for data in **Supplementary Figure 5 a** and **b**, respectively.

Supplementary Discussion 1 - 9

1. **AlphaFold predictions of IDRs in conditionally folded states:** Whereas some IDRs appear to be constitutively unstructured, others display conditional folding - they are unstructured in their *apo*, unmodified forms, but adopt partially or fully structured conformations upon post-translational modification and/or binding to other proteins[1–3]. Nevertheless, even the conditionally folded states of IDRs are challenging to characterize using empirical structural methods, due to their conformational heterogeneity[1,2,4,5]. Remarkably, AlphaFold, which derives structural models using multiple sequence alignments (MSAs) and coevolutionary analysis[6,7], is well-suited to analysis of conditionally folded IDRs, as it often predicts them in their conditionally folded states, even when the requisite post-translational modifications and/or binding partners are not included in the model[8,9]. Thus, although AlphaFold 2.3 does not permit specification of phosphorylation sites, the SMO / PKA-C models appear to capture phosphorylated, conditionally folded forms of SMO. In support of this: (a) modeling SMO / PKA-C interactions with AlphaFold3, which enables phosphorylation, produced models that are very similar to those generated by AlphaFold 2.3.0 **(Fig. 1b, Extended Data Fig. 1c)**; (b) the models agree closely with our HDX-MS, disulfide trapping, SPR, and functional studies of phosphorylated SMO / PKA-C complexes (**Fig. 4, 7**); (c) several PKA-C-binding secondary structural elements in the SMO pCT (e.g, pseudosubstrate motif, RII-like helix) are visible even in models of SMO alone. i.e., lacking PKA-C **(Extended Data Fig. 1b)**. These observations underscore AlphaFold's propensity to predict IDRs in their conditionally folded states.

2. **Two SMO / PKA-C models produced by AlphaFold 2.3.0:** AlphaFold 2.3.0 produces two classes of models for the SMO / PKA-C complex, which we term conformation 1 (conf1) and conformation 2 (conf2) **(Extended Data Fig. 1c)**. Alignment of the two models on their PKA-C subunit reveals that the SMO pCT / PKA-C module is rotated with respect to the SMO 7TM domain. As a result, the SMO pCT, including the pseudosubstrate motif, RII-like helix, and reentrant loop, are in near-superimposable configurations in both models, while the SMO ICLs engage one of two distinct surfaces of PKA-C in each model. Both models have high ipTM scores (79-84 for conf1, 80-81 for conf2) and are consistent with our HDX-MS studies. While we primarily refer to the AlphaFold 3 model (similar to conf1) in our manuscript, conf2 may also be physiologically relevant. Intriguingly, conformational heterogeneity is also seen in GPCR-arrestin complexes, where the arrestin can rotate, presenting different surfaces to the GPCR 7TM domain [10].

3. **HDX-MS studies of SMO / PKA-C complexes, and analysis of SMO pseudosubstrate motif and PKA-C active site cleft:** HDX-MS is a powerful technique to monitor changes in hydrogen bonding and solvent accessibility of backbone amides in two interacting proteins (SMO and PKA-C, in this case) upon complex formation, thereby providing a peptide-level readout of conformational changes in each protein upon binding to one another. To enable our HDX-MS studies, we utilized disulfide trapping to stabilize the SMO / PKA-C complex. This strategy was advantageous because it (a) prevented untoward complex dissociation, and (b) allowed us to trap the nonphosphorylated state of the complex, permitting explicit comparison to the phosphorylated state, and thereby revealing how phosphorylation affects this complex. Indeed, despite the high relative concentrations of SMO and PKA-C in these disulfide-trapped complexes, little protection occurs at SMO helix 8 or the RII-like helix unless SMO is phosphorylated **(Fig. 6)**, highlighting the importance of phosphorylation for stabilizing the SMO pCT and facilitating PKA-C binding.

   When interpreting our HDX-MS data, however, it is important to ensure that the results do not simply reflect artifactual, nonspecific interactions that may arise from covalently linking two proteins together into a complex. This scenario is unlikely because (a) SMO / PKA-C interaction results in protection of specific SMO and PKA-C regions known to be essential for each protein's functionality **(Extended Data Fig. 4a, b)**; (b)

protection depends on agonist-induced GRK2 phosphorylation, as we observed substantially less protection of disulfide-trapped SMO / PKA-C complexes bound to the SMO inverse agonist KAAD-cyclopamine (KAADcyc) and lacking phosphorylation **(Fig. 6d, Extended Data Fig. 7a, b)**. In contrast, if the HDX-MS results were nonspecific, protection would be independent of SMO phosphorylation and not involve specific, functionally relevant regions of SMO and PKA-C.

The SMO inhibitor sequence is protected upon binding PKA-C **(Extended Data Fig. 4d, e)** regardless of SMO phosphorylation, due to the disulfide trap (SMO L637C to PKA-C C199). However, under physiological conditions (in which SMO and PKA-C are not covalently linked), this interaction likely occurs in a SMO activity-dependent manner, i.e. only when SMO is phosphorylated. Interestingly, the PKA-C active site cleft and substrate-binding region (C helix, activation loop, P+1 loop) become more dynamic upon SMO binding **(Extended Data Fig. 4d, e).** This may be due to: (a) the SMO L637C – PKA-C C199 covalent bond trapping the kinase in a partially open conformation, as observed in disulfide-trapped PKA holoenzymes[11,12], and (b) the elevated pH (8.0) used, which favors disulfide trapping[12] but likely hinders kinase domain closure due to the deprotonation of histidine H87 within the active-site cleft[13]. Thus, while SMO undergoes protection, parts of PKA-C become more dynamic, consistent with our model.

4. **Specificity of disulfide trapping across the RII-like helix:** Disulfide trapping at the RII-like region (PKA-C G136C + SMO S570C or A574C) yielded, in addition to the desired SMO / PKA-C product, several side products, including PKA-C dimers and a band resembling SMO/PKA-C complexes formed without the PKA-C cysteine substitution. However, such side products are not uncommon in chemical crosslinking or disulfide trapping experiments, and the desired crosslinked species is not always the predominant product (for example, see Chen et al., Nature 2021, on rhodopsin–GRK1 crosslinking[14]). Disulfide trapping is inherently prone to nonspecific products because disulfide bond formation is kinetically driven and essentially irreversible, particularly under the oxidizing conditions typically used in these assays (see “Methods”). This contrasts with equilibrium binding assays, in which weak or nonspecific interactions can be removed by extensive washing steps. Importantly, in our experiments the correctly trapped SMO / PKA-C species could be clearly resolved by SDS–PAGE and showed a specific dependence on both the SMO G136C mutation and SMO phosphorylation status, with signals exceeding those from the corresponding negative controls (wild-type SMO and nonphosphorylated SMO G136C), even though some background products persisted. To further limit nonspecific products, we titrated the concentration of PKA-C, which decreased the concentrations of these nonspecific products and demonstrated clear, specific disulfide trapping between PKA-C G136C and SMO S570C (**Extended Data Fig. 5b**). Together, these results indicate that disulfide trapping at the RII-like helix interface is both robust and specific.

5. **SPR studies of PKA-C interactions with wild-type and mutant SMO:** The SMO IFR→AAA mutation significantly impairs GLI transcriptional activation in cultured cells **(Fig. 5a)** and reduces SMO/PKA-C binding in the in vitro SPR assay **(Fig. 5 c-e)**, supporting the idea that this mutation disrupts the SMO RII-like helix/PKA-C hinge interface. The impact of this mutation is less pronounced *in vitro* than *in vivo*, possibly due to: (1) a higher sensitivity of the *in vivo* GLI transcriptional assay to changes in amounts of the SMO / PKA-C complex over a relatively narrow range; (2) a greater extent of GRK2/3 phosphorylation of SMO in our *in vitro* SPR assay compared to our cell-based transcriptional activity assays[15], which might stabilize the SMO / PKA-C complex and thereby blunt the deleterious effects of a mutation compared to an *in vivo* setting. Nevertheless, the weaker interaction of the SMO IFR → AAA mutant with PKA-C is consistent with our hypothesis that the RII-like helix participates in SMO / PKA-C interactions. We also note the higher affinity observed for the SMO / PKA-C complex in this study (K_D = 82 nM) compared to our previous measurements on PKA-C interactions with a soluble, nonphosphorylated SMO pCT fragment (K_D = 752 nM)[16]; we attribute this to the use of phosphorylated, near-full-length SMO and a membrane-like environment (nanodiscs) in the present study, reflecting a more physiological state of the complex.

6. **Role of lipids in SMO / PKA-C interactions:** Lipids stabilize conventional GPCR signaling complexes by increasing local effector concentrations at membranes[14,17–23] and may play analogous roles in the SMO / PKA-C complex. In support of this hypothesis, HDX-MS studies revealed protection of a PKA-C C-lobe region (313-327) predicted by AlphaFold to face the membrane **(Fig. 7e, Extended Data Fig. 9b)**, suggestive of direct interactions with the membrane (or the detergent micelle *in vitro*). These interactions may be electrostatic: the SMO / PKA-C complex presents an electropositive surface near the membrane, favoring interactions with negatively charged phospholipid head groups **(Extended Data Fig. 9c)**, and phosphorylated residues in the SMO pCT engage the positively charged phospholipid counterions in MD simulations **(Extended Data Fig 6d)**. In addition, PKA-C is N-terminally myristoylated, and this group can mobilize from its hydrophobic PKA-C binding pocket to promote membrane interactions[24–30]. Although AlphaFold cannot yet incorporate membranes or N-myristoyl groups, it is intriguing that the PKA-C N-terminus points toward the membrane in our models, which may facilitate insertion of the myristoyl moiety **(Fig. 7a)**. Consistently, PKA-C's N-myristoyl group penetrated the membrane and remained there in MD simulations, stabilizing PKA-C's N-terminal helix relative to SMO and to the bilayer **(Extended Data Fig. 9d)**. Thus, lipid interactions may stabilize the SMO / PKA-C complex at the membrane inner leaflet.

7. **How GRK2/3 recognizes and phosphorylates active SMO:** When SMO binds to agonist, it undergoes a conformational change in which transmembrane helices 5 and 6 move outward to open a cavity at the cytoplasmic face of the 7TM domain[31]. This conformational change is stereotyped within the GPCR superfamily, as it is similar to ones that occur in conventional GPCRs upon ligand binding[32]. In the case of SMO, this conformational change is expected to promote interaction with PKA-C, as it enables recognition by GRK2/3 kinases, likely via insertion of the GRK aN helix into the SMO 7TM cavity leading to activation of the GRK kinase domain, as described for other GPCRs[14,15,33,34]. As with other GPCR substrates[14,34], GRKs are expected to rapidly dissociate from SMO following phosphorylation (allowing them to catalyze subsequent rounds of phosphorylation on additional SMO molecules). Nevertheless, future work—including structural studies of SMO–GRK complexes and detailed biochemical analysis of the SMO phosphorylation process—will be needed to define precisely how GRK2/3 engages and phosphorylates activated SMO.

8. **Limitations of AlphaFold modeling:** Our AlphaFold prediction shows high pLDDT scores within the RII-like helix and pseudosubstrate motif, indicating strong confidence in these regions. Lower scores elsewhere in the pCT reflect greater structural uncertainty. It is worth noting, however, that these low pLDDT regions may not arise from a deficiency in the AlphaFold prediction *per se,* but rather may reflect the lack of a single, stable conformation even in the presence of PKA-C. For example, the interaction predicted by AlphaFold between the SMO reentrant loop and 7TM cavity is stable in MD simulations, but the pLDDT scores are modest, raising the possibility that this loop might also engage additional binding surfaces (on SMO, PKA-C, or even other membrane proteins/lipids), thereby facilitating formation of the SMO / PKA-C complex via alternative mechanisms. Such conformational heterogeneity is common among many GPCRs[10,35,36], as exemplified by recent GPCR-arrestin structural studies[37–39] . AlphaFold generated one major class of models for the SMO / PKA-C complex as well as a second, less prevalent class**.** Future studies can address whether these two classes represent intermediates in the SMO-PKA signaling process vs unique signaling states that lead to distinct downstream outcomes, and may reveal additional conformations of the complex.

9. **Outstanding questions in SMO-GLI communication:** Our study raises several key questions regarding SMO-GLI communication in the Hh pathway. First, although we focused on SMO / PKA-C pseudosubstrate complexes in mouse, AlphaFold modeling predicts similar complexes in other metazoans **(Fig. 2c)**. Consistent with this, several key motifs—GRK phosphorylation sites and pseudosubstrate arginines—are at least partially conserved in *Drosophila* and contribute to Hh signaling[40,41], though some residue-level differences exist **(Extended Data Fig. 1g)**. Future biochemical studies in *Drosophila* and other species will

test whether our proposed structural mechanism is broadly conserved. Second, SMO / PKA-C complex formation in vertebrate cells could be assisted by scaffold proteins; in flies, Cos2 plays this role[42,43], although colocalization of vertebrate SMO and PKA-C in cilia (an organelle absent from most insect cells) may render a scaffold unnecessary. Identification and characterization of candidate SMO / PKA-C scaffolds in vertebrates will shed light on this issue. Third, in addition to directly binding and inhibiting PKA-C, SMO stimulation can lower ciliary PKA activity by mediating the ciliary exit of GPR161[44], a GPCR which both activates PKA-C via coupling to stimulatory G proteins and localizes PKA holoenzymes through its AKAP domain[45,46] (although these processes are not strictly required for Hh pathway activation[47,48]). How SMO activation triggers GPR161 ciliary exit, as well as how that process is coordinated with the direct SMO / PKA-C inhibition mechanism characterized here, remain to be elucidated. Fourth, given that SMO undergoes turnover in the cilium during pathway activation[49,50], whether PKA-C dissociates as SMO exits the cilium, whether the ciliary pool of PKA-C is replenished by a continuous influx of newly imported PKA-C as new SMO arrives, and how these dynamics might influence the magnitude or duration of Hh signal transduction, all remain unknown and are important directions for future investigation. Finally, our study focused on the SMO / PKA-C interaction, but SMO-GLI communication clearly involves additional factors besides PKA, such as Suppressor of Fused (SUFU), which binds directly to GLI proteins and blocks their activation[51,52]. Furthermore, *Drosophila* Smo activates the kinase Fused to phosphorylate the GLI ortholog Ci, and this phosphorylation is essential for full Ci activation[53,54]; similar processes may occur in mammals, although the identities of these GLI-activating kinases and the mechanisms by which SMO may affect their functions remain unresolved[55–57]. Understanding how all of these elements integrate with the direct SMO/PKA-C pathway represents an important future goal for the field.

REFERENCES:

1. Wright, P. E. & Dyson, H. J. Intrinsically disordered proteins in cellular signalling and regulation. *Nat. Rev. Mol. Cell Biol.* **16**, 18–29 (2015).
2. Bah, A. & Forman-Kay, J. D. Modulation of Intrinsically Disordered Protein Function by Post-translational Modifications. *J. Biol. Chem.* **291**, 6696–6705 (2016).
3. Holehouse, A. S. & Kragelund, B. B. The molecular basis for cellular function of intrinsically disordered protein regions. *Nat. Rev. Mol. Cell Biol.* (2023) doi:10.1038/s41580-023-00673-0.
4. Venkatakrishnan, A. J. *et al.* Structured and disordered facets of the GPCR fold. *Curr. Opin. Struct. Biol.* **27**, 129–137 (2014).
5. Flock, T., Weatheritt, R. J., Latysheva, N. S. & Babu, M. M. Controlling entropy to tune the functions of intrinsically disordered regions. *Curr. Opin. Struct. Biol.* **26**, 62–72 (2014).
6. Jumper, J. *et al.* Highly accurate protein structure prediction with AlphaFold. *Nature* (2021) doi:10.1038/s41586-021-03819-2.
7. Tunyasuvunakool, K. *et al.* Highly accurate protein structure prediction for the human proteome. *Nature* (2021) doi:10.1038/s41586-021-03828-1.

8. Alderson, T. R., Pritišanac, I., Kolarić, Đ., Moses, A. M. & Forman-Kay, J. D. Systematic identification of conditionally folded intrinsically disordered regions by AlphaFold2. *Proc. Natl. Acad. Sci. U. S. A.* **120**, e2304302120 (2023).
9. Piovesan, D., Monzon, A. M. & Tosatto, S. C. E. Intrinsic protein disorder and conditional folding in AlphaFoldDB. *Protein Sci.* **31**, e4466 (2022).
10. Chen, Q. & Tesmer, J. J. G. G protein-coupled receptor interactions with arrestins and GPCR kinases: The unresolved issue of signal bias. *J. Biol. Chem.* **298**, 102279 (2022).
11. First, E. A. & Taylor, S. S. Induced interchain disulfide bonding in cAMP-dependent protein kinase II. *J. Biol. Chem.* **259**, 4011–4014 (1984).
12. First, E. A., Bubis, J. & Taylor, S. S. Subunit interaction sites between the regulatory and catalytic subunits of cAMP-dependent protein kinase. Identification of a specific interchain disulfide bond. *J. Biol. Chem.* **263**, 5176–5182 (1988).
13. Cox, S. & Taylor, S. S. Kinetic analysis of cAMP-dependent protein kinase: mutations at histidine 87 affect peptide binding and pH dependence. *Biochemistry* **34**, 16203–16209 (1995).
14. Chen, Q. *et al.* Structures of rhodopsin in complex with G-protein-coupled receptor kinase 1. *Nature* **595**, 600–605 (2021).
15. Walker, M. F. *et al.* GRK2 kinases in the primary cilium initiate SMOOTHENED-PKA signaling in the Hedgehog cascade. *PLoS Biol.* **22**, e3002685 (2024).
16. Happ, J. T. *et al.* A PKA inhibitor motif within SMOOTHENED controls Hedgehog signal transduction. *Nat. Struct. Mol. Biol.* **29**, 990–999 (2022).
17. Janetzko, J. *et al.* Membrane phosphoinositides regulate GPCR-β-arrestin complex assembly and dynamics. *Cell* **185**, 4560-4573.e19 (2022).
18. Staus, D. P. *et al.* Structure of the M2 muscarinic receptor-β-arrestin complex in a lipid nanodisc. *Nature* **579**, 297–302 (2020).
19. Lally, C. C. M., Bauer, B., Selent, J. & Sommer, M. E. C-edge loops of arrestin function as a membrane anchor. *Nat. Commun.* **8**, 14258 (2017).
20. Marrari, Y., Crouthamel, M., Irannejad, R. & Wedegaertner, P. B. Assembly and trafficking of heterotrimeric G proteins. *Biochemistry* **46**, 7665–7677 (2007).

21. Higgins, J. B. & Casey, P. J. The role of prenylation in G-protein assembly and function. *Cell. Signal.* **8**, 433–437 (1996).

22. Thakur, N. *et al.* Anionic phospholipids control mechanisms of GPCR-G protein recognition. *Nat. Commun.* **14**, 794 (2023).

23. Strohman, M. J. *et al.* Local membrane charge regulates β2 adrenergic receptor coupling to Gi3. *Nat. Commun.* **10**, 2234 (2019).

24. Gaffarogullari, E. C. *et al.* A myristoyl/phosphoserine switch controls cAMP-dependent protein kinase association to membranes. *J. Mol. Biol.* **411**, 823–836 (2011).

25. Xiong, W.-H., Qin, M. & Zhong, H. Myristoylation alone is sufficient for PKA catalytic subunits to associate with the plasma membrane to regulate neuronal functions. *Proc. Natl. Acad. Sci. U. S. A.* **118**, (2021).

26. Tillo, S. E. *et al.* Liberated PKA Catalytic Subunits Associate with the Membrane via Myristoylation to Preferentially Phosphorylate Membrane Substrates. *Cell Rep.* **19**, 617–629 (2017).

27. Zhang, P. *et al.* An Isoform-Specific Myristylation Switch Targets Type II PKA Holoenzymes to Membranes. *Structure* **23**, 1563–1572 (2015).

28. Mihályi, C., Iordanov, I., Szöllősi, A. & Csanády, L. Structural determinants of protein kinase A essential for CFTR channel activation. *bioRxiv* (2024) doi:10.1101/2024.05.27.596024.

29. Fiedorczuk, K. *et al.* The structures of protein kinase A in complex with CFTR: mechanisms of phosphorylation and reversible activation. *bioRxiv* (2024) doi:10.1101/2024.05.28.596263.

30. Gold, M. G. Swimming regulations for protein kinase A catalytic subunit. *Biochem. Soc. Trans.* **47**, 1355–1366 (2019).

31. Deshpande, I. *et al.* Smoothened stimulation by membrane sterols drives Hedgehog pathway activity. *Nature* **571**, 284–288 (2019).

32. Weis, W. I. & Kobilka, B. K. The molecular basis of G protein-coupled receptor activation. *Annu. Rev. Biochem.* **87**, 897–919 (2018).

33. Arveseth, C. D. *et al.* Smoothened transduces Hedgehog signals via activity-dependent sequestration of PKA catalytic subunits. *PLoS Biol.* **19**, e3001191 (2021).

34. Duan, J. *et al.* GPCR activation and GRK2 assembly by a biased intracellular agonist. *Nature* **620**, 676–681 (2023).

35. Wingler, L. M. & Lefkowitz, R. J. Conformational basis of G protein-coupled receptor signaling versatility. *Trends Cell Biol.* **30**, 736–747 (2020).

36. Hilger, D., Masureel, M. & Kobilka, B. K. Structure and dynamics of GPCR signaling complexes. *Nat. Struct. Mol. Biol.* **25**, 4–12 (2018).

37. Maharana, J. *et al.* Molecular insights into atypical modes of β-arrestin interaction with seven transmembrane receptors. *Science* **383**, 101–108 (2024).

38. Chen, Q. *et al.* Effect of phosphorylation barcodes on arrestin binding to a chemokine receptor. *Nature* **643**, 280–287 (2025).

39. Grimes, J. *et al.* Plasma membrane preassociation drives β-arrestin coupling to receptors and activation. *Cell* **186**, 2238-2255.e20 (2023).

40. Maier, D., Cheng, S., Faubert, D. & Hipfner, D. R. A broadly conserved g-protein-coupled receptor kinase phosphorylation mechanism controls Drosophila smoothened activity. *PLoS Genet.* **10**, e1004399 (2014).

41. Doll, R. M., Boutros, M. & Port, F. A temperature-tolerant CRISPR base editor mediates highly efficient and precise gene editing in Drosophila. *Sci Adv* **9**, eadj1568 (2023).

42. Ranieri, N., Thérond, P. P. & Ruel, L. Switch of PKA substrates from Cubitus interruptus to Smoothened in the Hedgehog signalosome complex. *Nat. Commun.* **5**, 1–14 (2014).

43. Li, S., Ma, G., Wang, B. & Jiang, J. Hedgehog induces formation of PKA-Smoothened complexes to promote Smoothened phosphorylation and pathway activation. *Sci. Signal.* **7**, ra62 (2014).

44. Hilgendorf, K. I., Myers, B. R. & Reiter, J. F. Emerging mechanistic understanding of cilia function in cellular signalling. *Nat. Rev. Mol. Cell Biol.* 1–19 (2024).

45. Hoppe, N. *et al.* GPR161 structure uncovers the redundant role of sterol-regulated ciliary cAMP signaling in the Hedgehog pathway. *Nat. Struct. Mol. Biol.* **31**, 667–677 (2024).

46. Pal, K. *et al.* Smoothened determines β-arrestin-mediated removal of the G protein-coupled receptor Gpr161 from the primary cilium. *J. Cell Biol.* **212**, 861–875 (2016).

47. Pusapati, G. V. *et al.* G protein-coupled receptors control the sensitivity of cells to the morphogen Sonic Hedgehog. *Sci. Signal.* **11**, (2018).

48. Hwang, S.-H., Somatilaka, B. N., White, K. & Mukhopadhyay, S. Ciliary and extraciliary Gpr161 pools repress hedgehog signaling in a tissue-specific manner. *Elife* **10**, (2021).

49. Kim, J. *et al.* Simultaneous measurement of smoothened entry into and exit from the primary cilium. *PLoS One* **9**, e104070 (2014).

50. Pusapati, G. V. *et al.* CRISPR Screens Uncover Genes that Regulate Target Cell Sensitivity to the Morphogen Sonic Hedgehog. *Dev. Cell* **44**, 113-129.e8 (2018).

51. Ohlmeyer, J. T. & Kalderon, D. Hedgehog stimulates maturation of Cubitus interruptus into a labile transcriptional activator. *Nature* **396**, 749–753 (1998).

52. Svärd, J. *et al.* Genetic elimination of suppressor of fused reveals an essential repressor function in the mammalian hedgehog signaling pathway. *Dev. Cell* **10**, 409 (2006).

53. Thérond, P. P., Knight, J. D., Kornberg, T. B. & Bishop, J. M. Phosphorylation of the fused protein kinase in response to signaling from hedgehog. *Proc. Natl. Acad. Sci. U. S. A.* **93**, 4224–4228 (1996).

54. Zhou, Q. & Kalderon, D. Hedgehog activates fused through phosphorylation to elicit a full spectrum of pathway responses. *Dev. Cell* **20**, 802–814 (2011).

55. Wilson, C. W. *et al.* Fused has evolved divergent roles in vertebrate Hedgehog signalling and motile ciliogenesis. *Nature* **459**, 98–102 (2009).

56. Niewiadomski, P. *et al.* Gli protein activity is controlled by multisite phosphorylation in vertebrate Hedgehog signaling. *Cell Rep.* **6**, 168–181 (2014).

57. Zhou, M., Han, Y. & Jiang, J. Ulk4 promotes Shh signaling by regulating Stk36 ciliary localization and Gli2 phosphorylation. *Elife* **12**, (2023).
