# Supplementary material for: Structural mechanism for noncanonical GPCR signaling in the Hedgehog pathway
Source: Nat Struct Mol Biol. 2026 Apr 30;33(5):795–809. doi: 10.1038/s41594-026-01800-z (PMC13186710; doi:10.1038/s41594-026-01800-z)
Supplement: Supplementary file 2 — Reporting Summary [file 41594_2026_1800_MOESM2_ESM.pdf]

nature portfolio

Corresponding author(s): Myers, Benjamin R.

Last updated by author(s): Mar 15, 2026

Reporting Summary

Nature Portfolio wishes to improve the reproducibility of the work that we publish. This form provides structure for consistency and transparency in reporting. For further information on Nature Portfolio policies, see our Editorial Policies and the Editorial Policy Checklist.

Statistics

For all statistical analyses, confirm that the following items are present in the figure legend, table legend, main text, or Methods section.

n/a	Confirmed	
☐	☒	The exact sample size (*n*) for each experimental group/condition, given as a discrete number and unit of measurement
☐	☒	A statement on whether measurements were taken from distinct samples or whether the same sample was measured repeatedly
☐	☒	The statistical test(s) used AND whether they are one- or two-sided<br>*Only common tests should be described solely by name; describe more complex techniques in the Methods section.*
☒	☐	A description of all covariates tested
☒	☐	A description of any assumptions or corrections, such as tests of normality and adjustment for multiple comparisons
☐	☒	A full description of the statistical parameters including central tendency (e.g. means) or other basic estimates (e.g. regression coefficient) AND variation (e.g. standard deviation) or associated estimates of uncertainty (e.g. confidence intervals)
☐	☒	For null hypothesis testing, the test statistic (e.g. *F*, *t*, *r*) with confidence intervals, effect sizes, degrees of freedom and *P* value noted<br>*Give P values as exact values whenever suitable.*
☒	☐	For Bayesian analysis, information on the choice of priors and Markov chain Monte Carlo settings
☒	☐	For hierarchical and complex designs, identification of the appropriate level for tests and full reporting of outcomes
☒	☐	Estimates of effect sizes (e.g. Cohen's *d*, Pearson's *r*), indicating how they were calculated

Our web collection on statistics for biologists contains articles on many of the points above.

Software and code

Policy information about availability of computer code

Data collection	Leica Application Suite X 3.5.7 (imaging acquisition), Biorad ImageLab 6.1.0 (SDS-PAGE, western blots), Tecan SparkControl 2.2 (BRET measurements), Biacore T200 Evaluation Software 3.0 (SPR)
Data analysis	AlphaFold 3; AlphaFold 2.3.0; PyMOL (v.2.5); Biacore 3000 Evaluation Software 4.1.1; GraphPad Prism 8.0.1; CHARMM-GUI; ImageJ; Protein Lynx Global Server v3.0 (PLGS); MaxQuant data analysis algorithm (version 1.6.12.0); Skyline (version 20.2.0.343). Deuteros 2.0 (HDXMS); CryoSPARC (cryoEM data analysis). Custom code was used for CD data processing and can be accessed in Zenodo (https://doi.org/10.5281/zenodo.18970396), as described in Code Availability Statement.

For manuscripts utilizing custom algorithms or software that are central to the research but not yet described in published literature, software must be made available to editors and reviewers. We strongly encourage code deposition in a community repository (e.g. GitHub). See the Nature Portfolio guidelines for submitting code & software for further information.

Data

Policy information about availability of data

All manuscripts must include a data availability statement. This statement should provide the following information, where applicable:

- Accession codes, unique identifiers, or web links for publicly available datasets
- A description of any restrictions on data availability
- For clinical datasets or third party data, please ensure that the statement adheres to our policy

AlphaFold models of the SMO / PKA-C complexes have been deposited in the Zenodo database (see "Methods"). The molecular dynamics simulation trajectories have been deposited in GPCRmd (see "Methods"). Mass spectrometry data have been deposited at the Panorama server (phosphoproteomics) or ProteomeXchange (HDX-MS) (see "Methods"). CryoEM maps have been deposited at EMDB (see "Methods"). All unique biological materials are available upon request from the authors.

Research involving human participants, their data, or biological material

Policy information about studies with human participants or human data. See also policy information about sex, gender (identity/presentation), and sexual orientation and race, ethnicity and racism.

Reporting on sex and gender	*Use the terms sex (biological attribute) and gender (shaped by social and cultural circumstances) carefully in order to avoid confusing both terms. Indicate if findings apply to only one sex or gender; describe whether sex and gender were considered in study design; whether sex and/or gender was determined based on self-reporting or assigned and methods used. Provide in the source data disaggregated sex and gender data, where this information has been collected, and if consent has been obtained for sharing of individual-level data; provide overall numbers in this Reporting Summary. Please state if this information has not been collected. Report sex- and gender-based analyses where performed, justify reasons for lack of sex- and gender-based analysis.*
Reporting on race, ethnicity, or other socially relevant groupings	*Please specify the socially constructed or socially relevant categorization variable(s) used in your manuscript and explain why they were used. Please note that such variables should not be used as proxies for other socially constructed/relevant variables (for example, race or ethnicity should not be used as a proxy for socioeconomic status). Provide clear definitions of the relevant terms used, how they were provided (by the participants/respondents, the researchers, or third parties), and the method(s) used to classify people into the different categories (e.g. self-report, census or administrative data, social media data, etc.) Please provide details about how you controlled for confounding variables in your analyses.*
Population characteristics	*Describe the covariate-relevant population characteristics of the human research participants (e.g. age, genotypic information, past and current diagnosis and treatment categories). If you filled out the behavioural & social sciences study design questions and have nothing to add here, write "See above."*
Recruitment	*Describe how participants were recruited. Outline any potential self-selection bias or other biases that may be present and how these are likely to impact results.*
Ethics oversight	*Identify the organization(s) that approved the study protocol.*

Note that full information on the approval of the study protocol must also be provided in the manuscript.

Field-specific reporting

Please select the one below that is the best fit for your research. If you are not sure, read the appropriate sections before making your selection.

☒ Life sciences ☐ Behavioural & social sciences ☐ Ecological, evolutionary & environmental sciences

For a reference copy of the document with all sections, see nature.com/documents/nr-reporting-summary-flat.pdf

Life sciences study design

All studies must disclose on these points even when the disclosure is negative.

Sample size	No statistical methods were used to pre-determine sample size. Sample sizes were determined based on our extensive prior experience and are standard for peer-reviewed studies in our field. The size of each sample (n) is reported in the relevant Figure Legend or in the Method section.
Data exclusions	No data were excluded.
Replication	All results were replicated in at least two independent experiments performed on two separate occasions. All attempts at replication were successful.
Randomization	All experiments were initiated from multiple independent pools of transfected cells derived from the same frozen vial of cells, and then subjected to indicated experimental conditions. Randomization was not applicable to our study, as is standard for biochemical, cultured cell,

	and biophysical assays in our field.
Blinding	Blinding was not applicable to our study, as is standard for biochemical, cultured cell, and biophysical assays in our field.

Reporting for specific materials, systems and methods

We require information from authors about some types of materials, experimental systems and methods used in many studies. Here, indicate whether each material, system or method listed is relevant to your study. If you are not sure if a list item applies to your research, read the appropriate section before selecting a response.

Materials & experimental systems

n/a	Involved in the study
☐	☒ Antibodies
☐	☒ Eukaryotic cell lines
☒	☐ Palaeontology and archaeology
☒	☐ Animals and other organisms
☒	☐ Clinical data
☒	☐ Dual use research of concern
☒	☐ Plants

Methods

n/a	Involved in the study
☒	☐ ChIP-seq
☒	☐ Flow cytometry
☒	☐ MRI-based neuroimaging

Antibodies

Antibodies used	1. Mouse anti-FLAG M2 – Sigma-Aldrich (F3165)<br>2. Rat anti-Arl13b – BiCell Scientific (90413)<br>3. Rabbit anti-AcTubulin – Enzo Life Sciences (BLM-SA452-0100)<br>4. Rabbit anti-pSMO – 7TM Antibodies (7TM0239A)<br>5. Mouse anti-myc, Clone 4A6 – Millipore (05-724)
Validation	1. Validated using immunoblot of HEK293 lysates heterologously expressing FLAG -tagged proteins.<br>2. Validated via Western blot (lysate from control vs Arl13b knockout fibroblasts)<br>3. Validated via microscopy (staining of cells with or without AcTubulin=positive primary cilia)<br>4. Validated via microscopy (ciliary localization of pSMO in wild-type vs. Smo-/- fibroblasts)<br>5. Validated via microscopy (cells expressing a myc-tagged Scc1 protein)

Eukaryotic cell lines

Policy information about cell lines and Sex and Gender in Research

Cell line source(s)	1. HEK293-Freestyle: Thermo Fisher Scientific<br>2. HEK293FT: Thermo Fisher Scientific<br>3. IMCD3 Flp-in: gift from P. Jackson, Stanford University<br>4. Smo-/- MEFs: gift from P. Beachy, Stanford University<br>5. Sf9 cells; gift from K.C. Garcia, Stanford University<br>6. HEK293 Flp-in T-rex: gift from D. Julius, UCSF<br>7. NIH3T3 Flp-in: gift from X. Ge, UC-Merced
Authentication	None of the cell lines were authenticated
Mycoplasma contamination	Cells are tested annually for mycoplasma contamination (MycoAlert Mycoplasma Detection Kit, Lonza), and all tested negative.
Commonly misidentified lines<br>(See ICLAC register)	no commonly misidentified cell lines were used

Plants

Seed stocks	*Report on the source of all seed stocks or other plant material used. If applicable, state the seed stock centre and catalogue number. If plant specimens were collected from the field, describe the collection location, date and sampling procedures.*
Novel plant genotypes	*Describe the methods by which all novel plant genotypes were produced. This includes those generated by transgenic approaches, gene editing, chemical/radiation-based mutagenesis and hybridization. For transgenic lines, describe the transformation method, the number of independent lines analyzed and the generation upon which experiments were performed. For gene-edited lines, describe the editor used, the endogenous sequence targeted for editing, the targeting guide RNA sequence (if applicable) and how the editor was applied.*
Authentication	*Describe any authentication procedures for each seed stock used or novel genotype generated. Describe any experiments used to assess the effect of a mutation and, where applicable, how potential secondary effects (e.g. second site T-DNA insertions, mosiacism, off-target gene editing) were examined.*
