# Supplementary material for: Structural mechanism for noncanonical GPCR signaling in the Hedgehog pathway
Source: Nat Struct Mol Biol. 2026 Apr 30;33(5):795–809. doi: 10.1038/s41594-026-01800-z (PMC13186710; doi:10.1038/s41594-026-01800-z)
Supplement: Supplementary file 5 — Cryo-EM sample preparation and data processing. [file 41594_2026_1800_MOESM5_ESM.pdf]

Sample 1: SMO / PKA-C mixed immediately prior to grid preparation

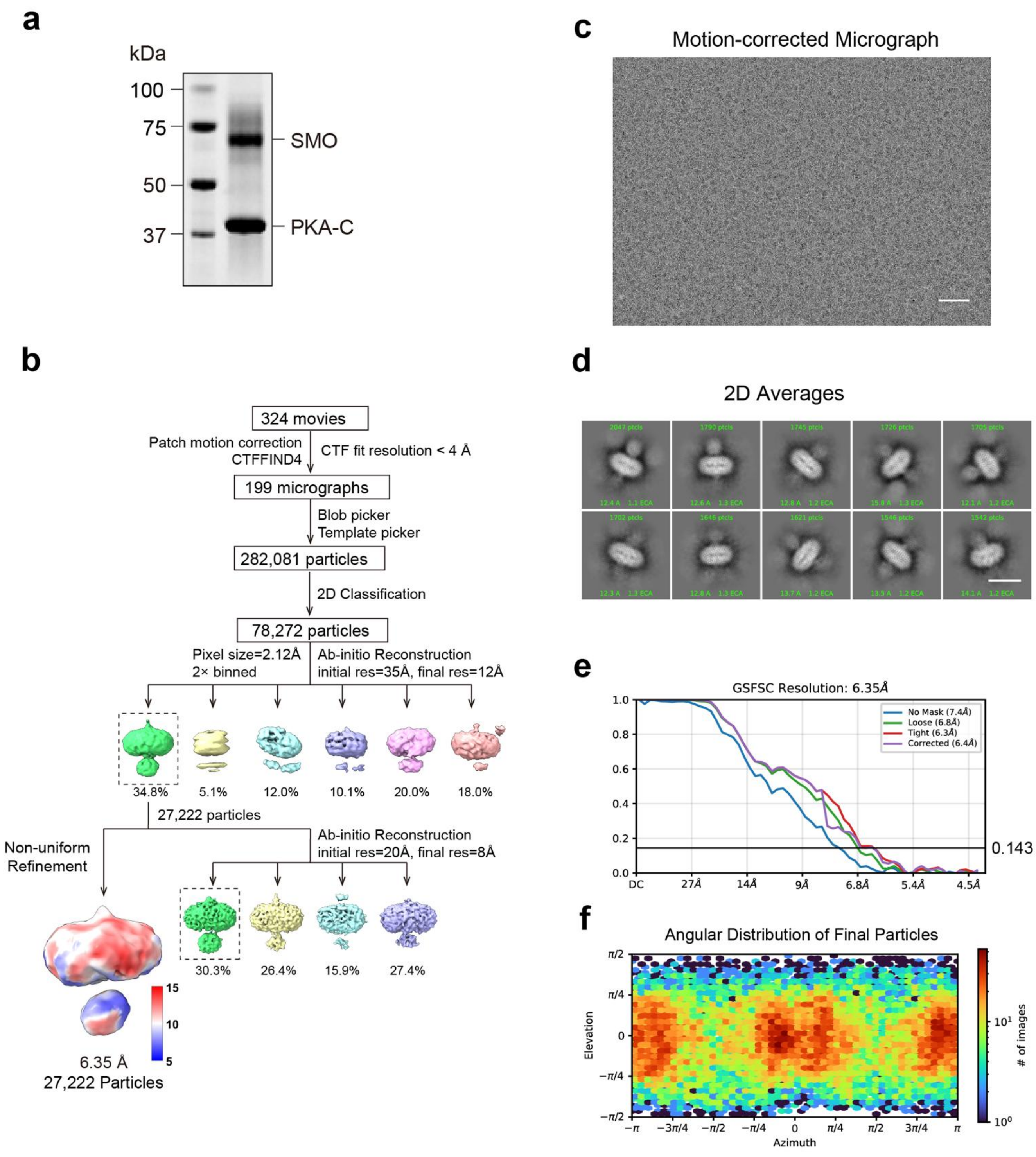

Sample 1: SMO / PKA-C mixed immediately prior to grid preparation. a, SDS-PAGE analysis. **b,** Workflow of cryoEM data processing. **c,** Representative cryoEM micrograph (scale bar: 50 nm). **d,** Representative 2D class averages (scale bar: 10 nm). **e,** Gold-standard Fourier shell correlation (FSC) curves of the EM map. **f,** Angular distribution plot of final particles.

Sample 2: SMO / PKA-C complex in MSP1E3D1 nanodiscs

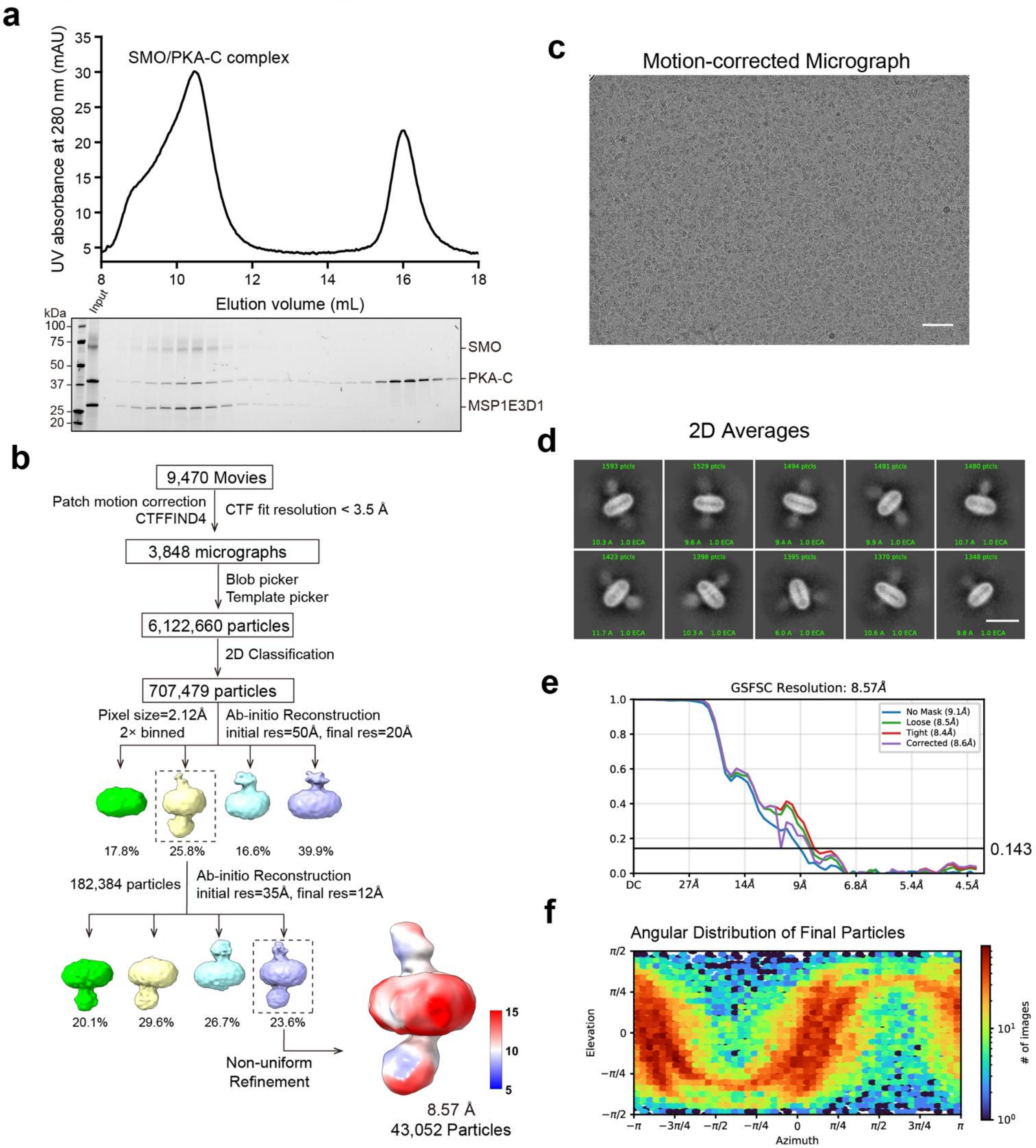

Sample 2: SMO / PKA-C complex in MSP1E3D1 nanodiscs. a, Size-exclusion chromatography and SDS-PAGE analysis. **b,** Workflow of cryoEM data processing. **c,** Representative cryoEM micrograph (scale bar: 50 nm). **d,** Representative 2D class averages (scale bar: 10 nm). **e,** Gold-standard Fourier shell correlation (FSC) curves of the EM map. **f,** Angular distribution plot of final particles.

Sample 3: Disulfide-trapped SMO-L637C / PKA-C complex

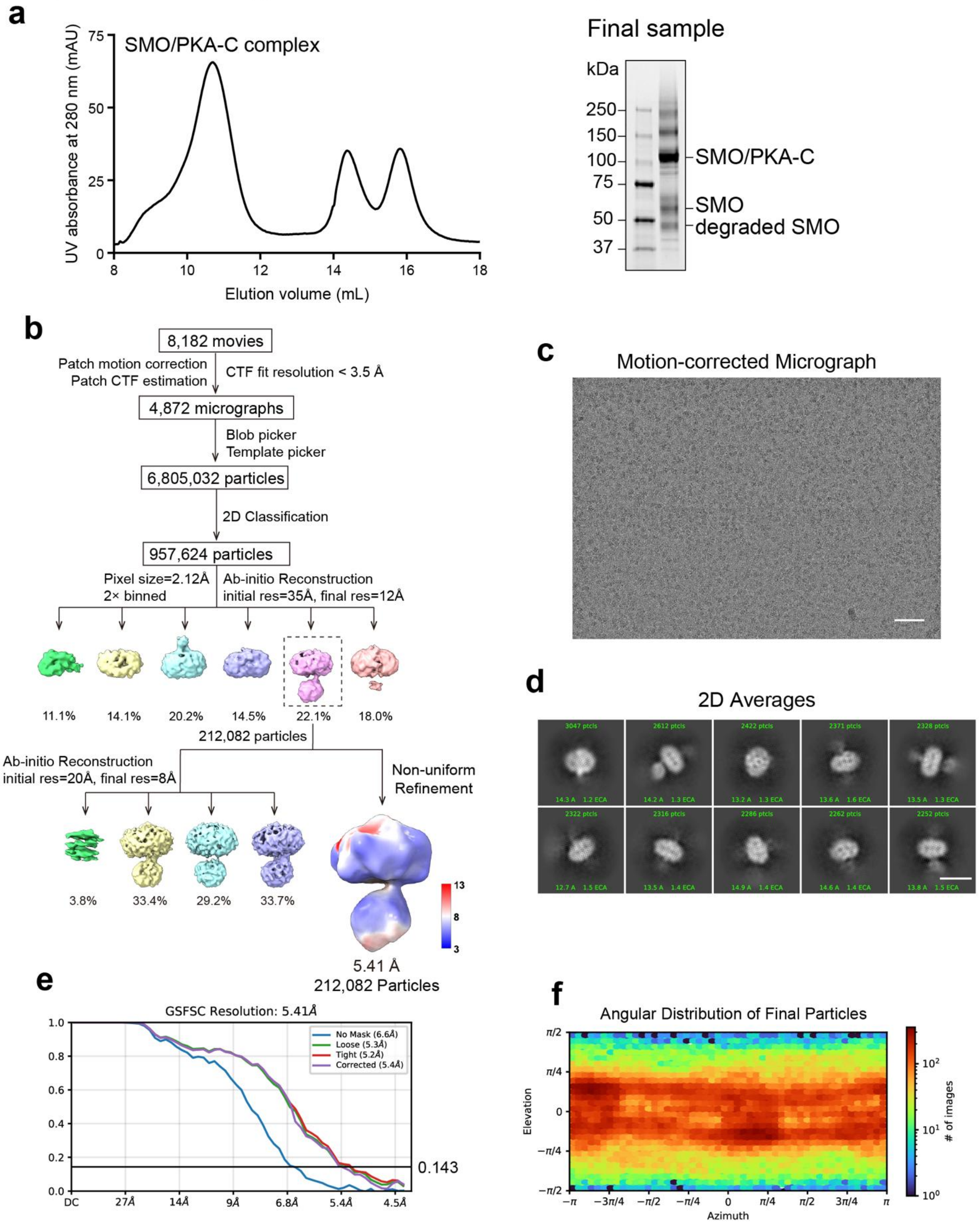

Sample 3: Disulfide-trapped SMO-L637C / PKA-C complex. a, Size-exclusion chromatography and SDS-PAGE analysis. **b,** Workflow of cryoEM data processing. **c,** Representative cryoEM micrograph

(scale bar: 50 nm). **d,** Representative 2D class averages (scale bar: 10 nm). **e,** Gold-standard Fourier shell correlation (FSC) curves of the EM map. **f,** Angular distribution plot of final particles.

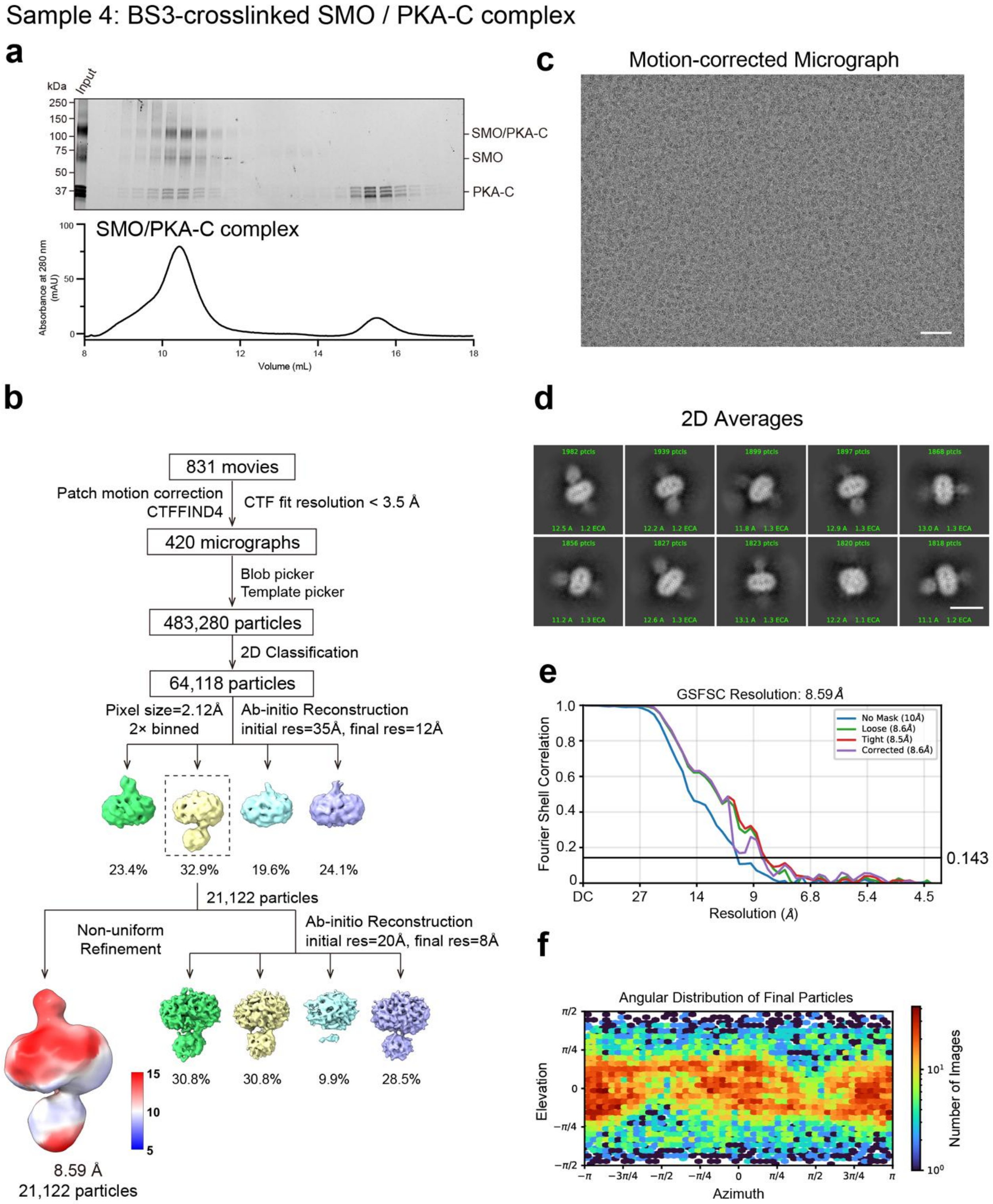

Sample 4: BS3-crosslinked SMO / PKA-C complex. a, Size-exclusion chromatography and SDS-PAGE analysis. **b,** Workflow of cryoEM data processing. **c,** Representative cryoEM micrograph (scale

bar: 50 nm). **d,** Representative 2D class averages (scale bar: 10 nm). **e,** Gold-standard Fourier shell correlation (FSC) curves of the EM map. **f,** Angular distribution plot of final particles.

Sample 5: EDC/Sulfo-NHS-crosslinked SMO / PKA-C complex

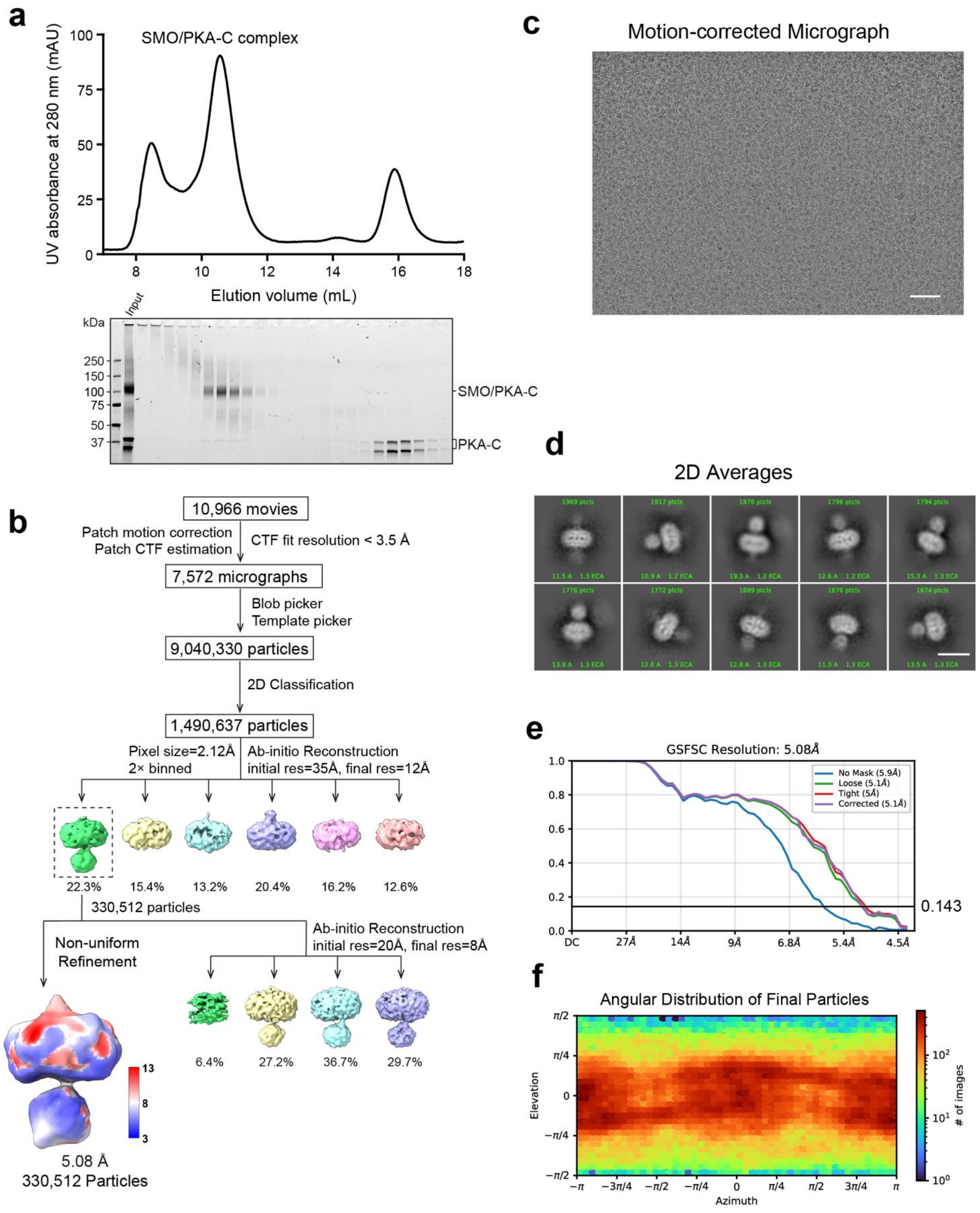

Sample 5: EDC/Sulfo-NHS-crosslinked SMO / PKA-C complex. a, Size-exclusion chromatography and SDS-PAGE analysis. **b,** Workflow of cryoEM data processing. **c,** Representative cryoEM micrograph (scale bar: 50 nm). **d,** Representative 2D class averages (scale bar: 10 nm). **e,** Gold-

standard Fourier shell correlation (FSC) curves of the EM map. **f,** Angular distribution plot of final particles.

Sample 6: SMO / PKA-C complex subjected to dual EDC/Sulfo-NHS and BS3 crosslinking

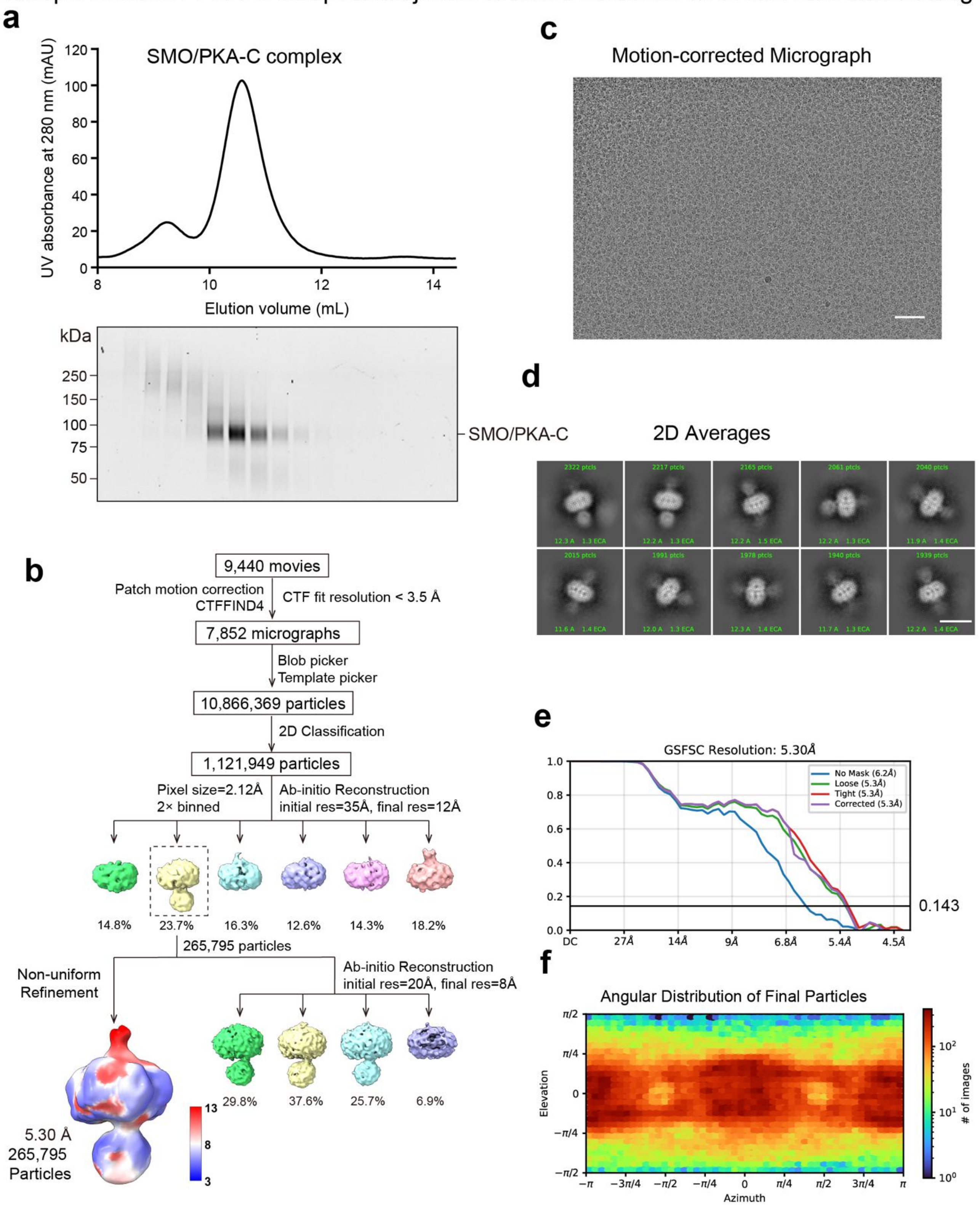

Sample 6: SMO / PKA-C complex subjected to dual EDC/Sulfo-NHS and BS3 crosslinking. a, Size-exclusion chromatography and SDS-PAGE analysis. **b,** Workflow of cryoEM data processing. **c,** Representative cryoEM micrograph (scale bar: 50 nm). **d,** Representative 2D class averages (scale bar: 10 nm). **e,** Gold-standard Fourier shell correlation (FSC) curves of the EM map. **f,** Angular distribution plot of final particles.

Sample 1:

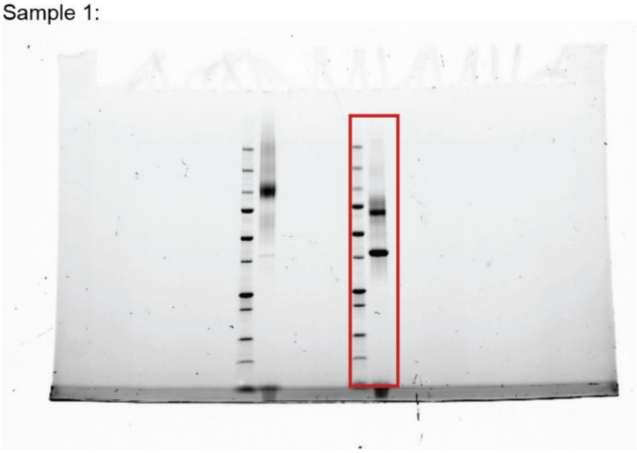

Sample 2:

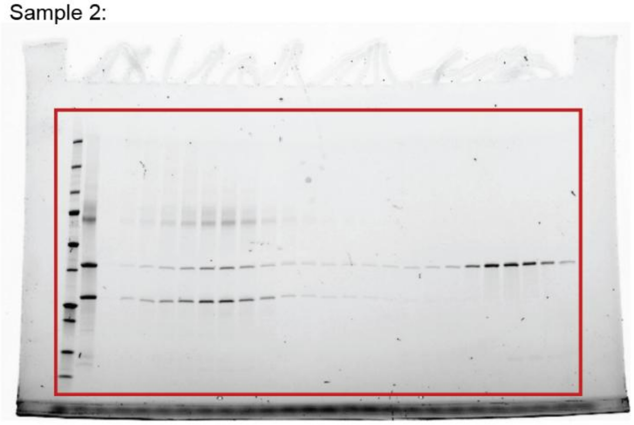

Sample 3:

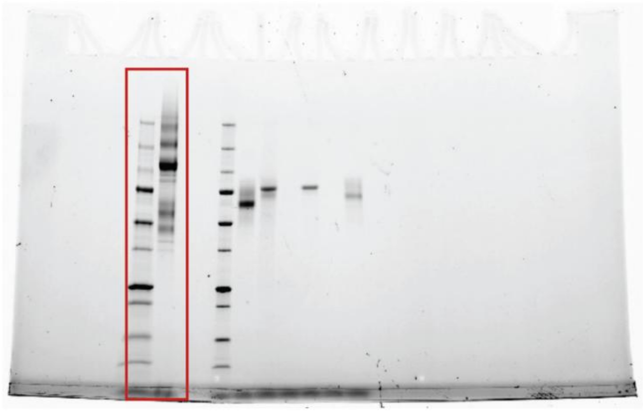

Sample 4:

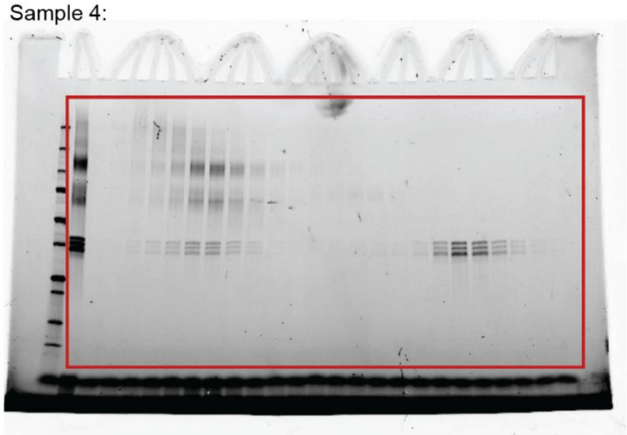

Sample 5:

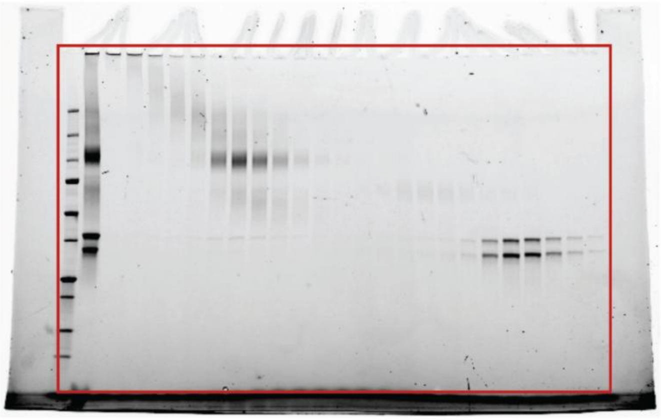

Sample 6:

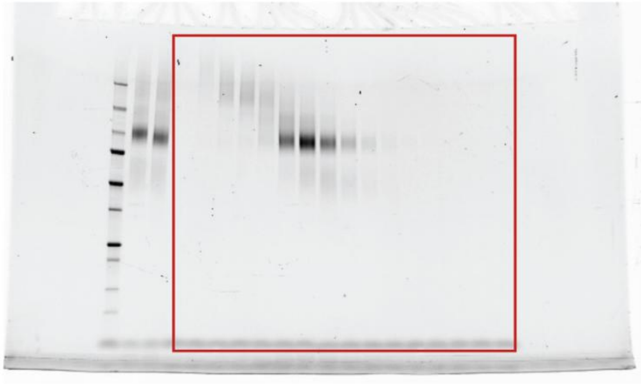

Uncropped SDS-PAGE gels corresponding to samples above
