# Supplementary figures and images for: Structural mechanism for noncanonical GPCR signaling in the Hedgehog pathway
Source: Nat Struct Mol Biol. 2026 Apr 30;33(5):795–809. doi: 10.1038/s41594-026-01800-z (PMC13186710; doi:10.1038/s41594-026-01800-z)

Source Data Fig. 2b

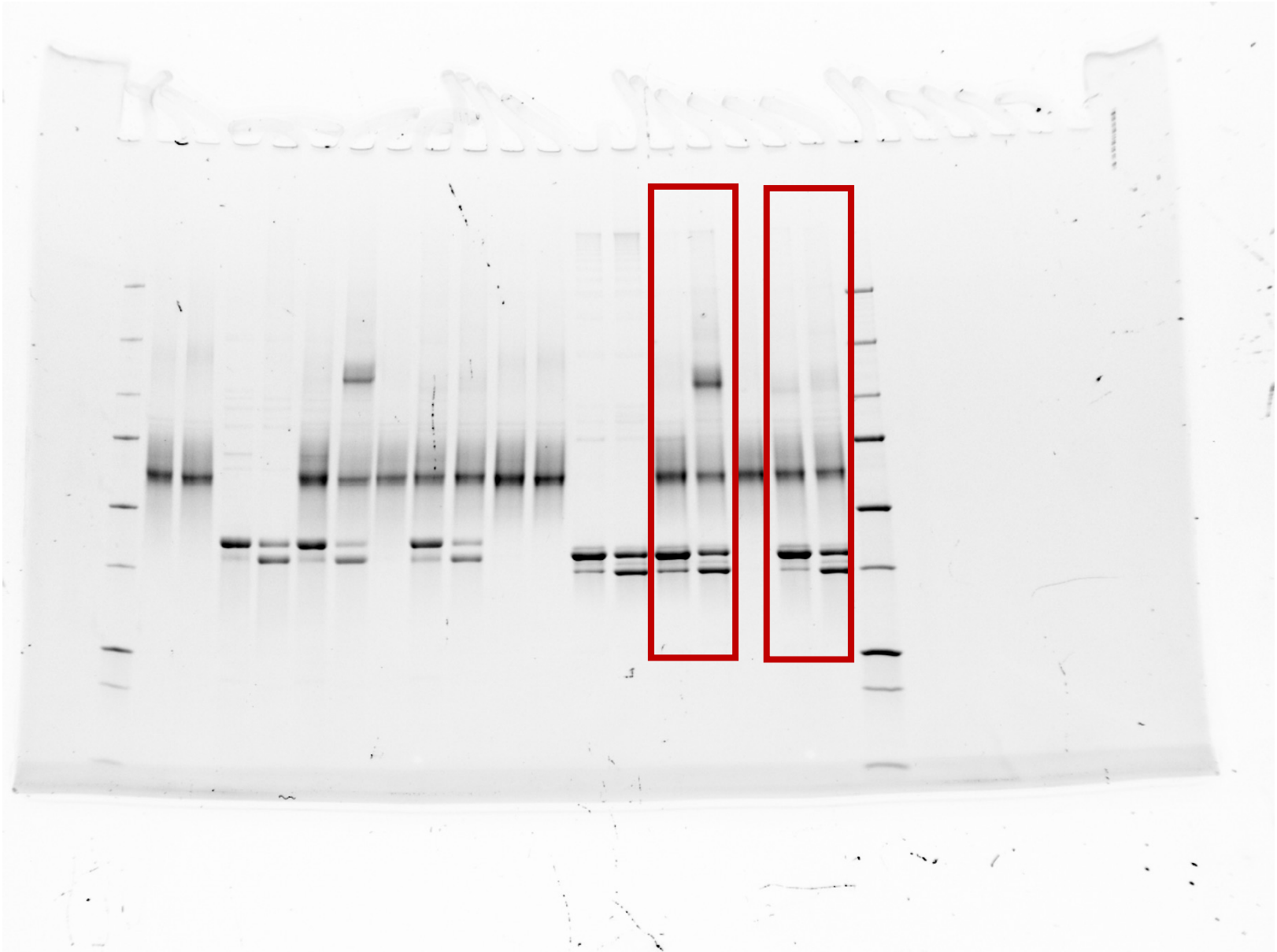

Supplement: Supplementary file 7 — Unprocessed SDS–PAGE gel image. [file 41594_2026_1800_MOESM7_ESM.pdf]

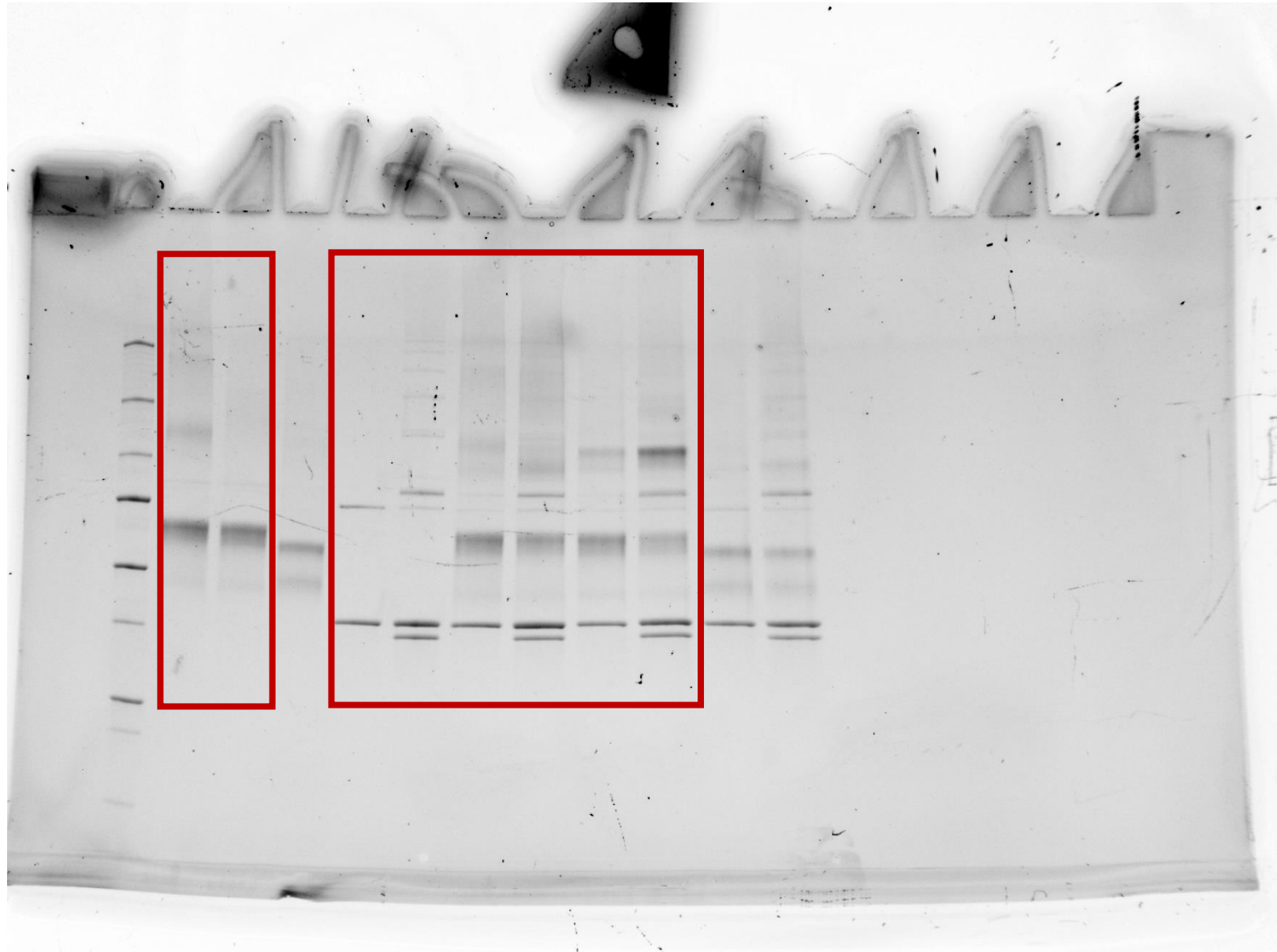

Supplement: Supplementary file 8 — Unprocessed SDS–PAGE gel image. [file 41594_2026_1800_MOESM8_ESM.pdf]

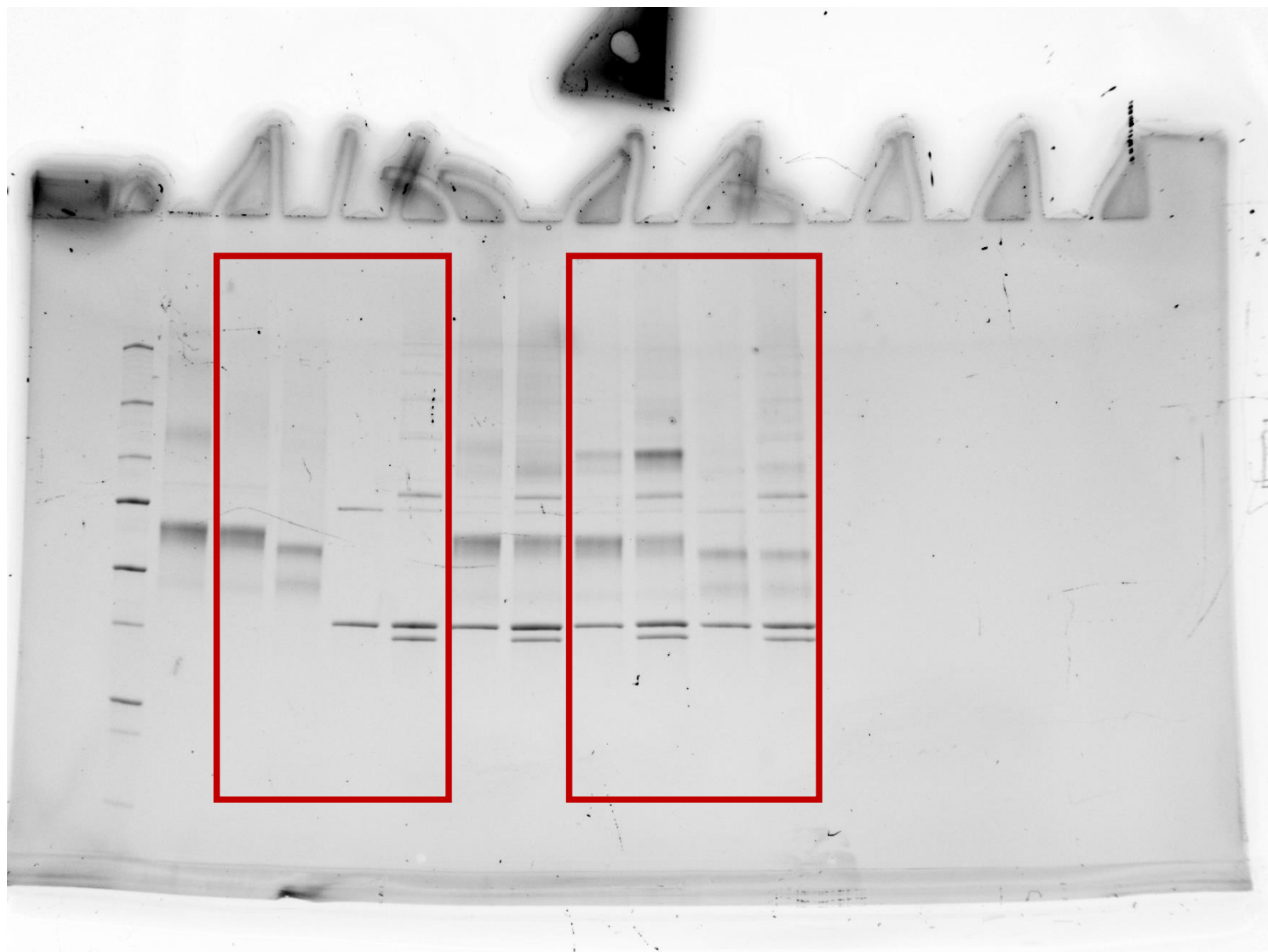

Supplement: Supplementary file 10 — GLI reporter unprocessed data and unprocessed SDS–PAGE gel image. [file 41594_2026_1800_MOESM10_ESM.pdf]

Source Data ED fig. 3a

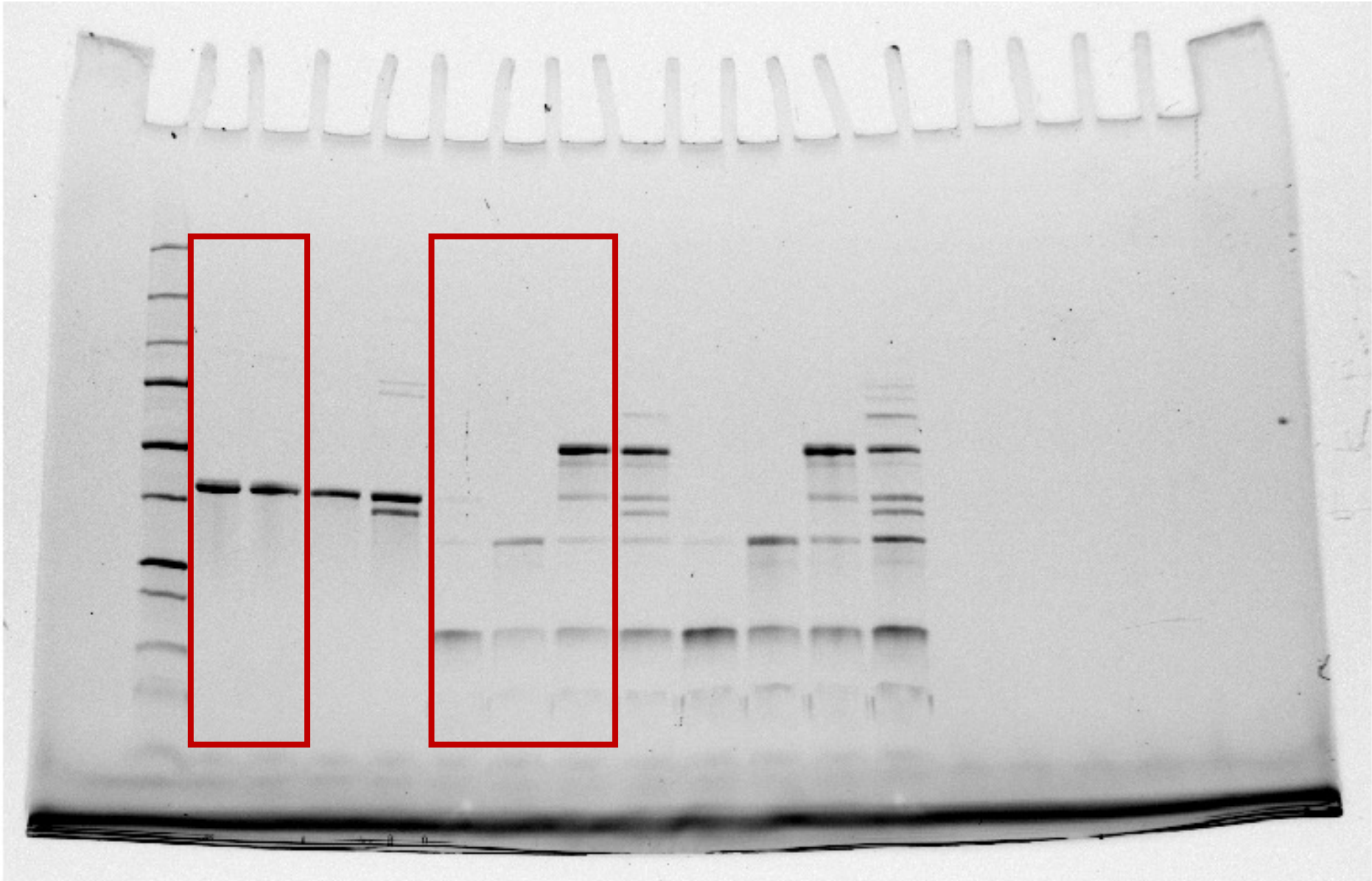

Source Data ED fig. 3b

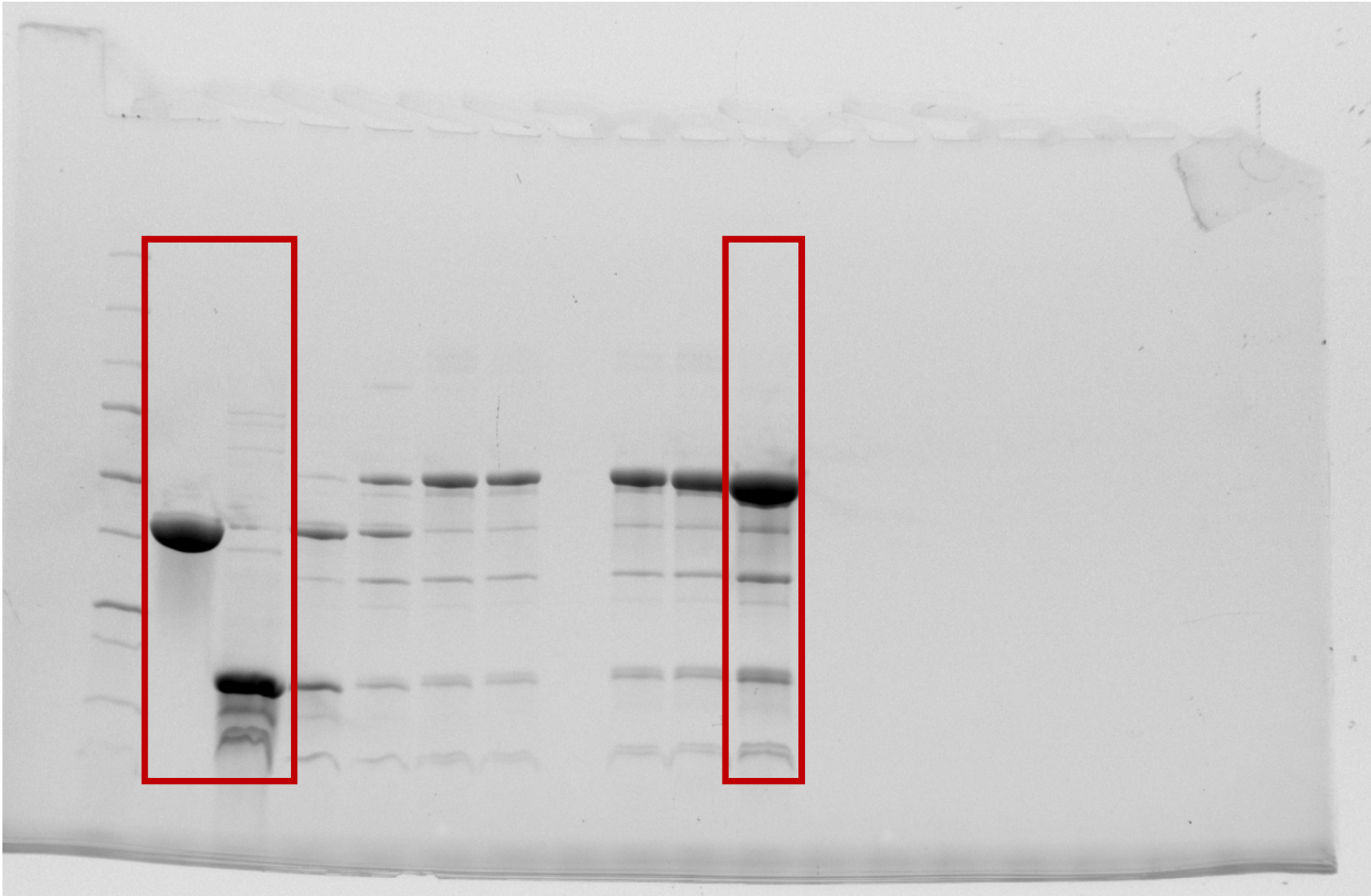

Supplement: Supplementary file 14 — Unprocessed SDS–PAGE gel image. [file 41594_2026_1800_MOESM14_ESM.pdf]

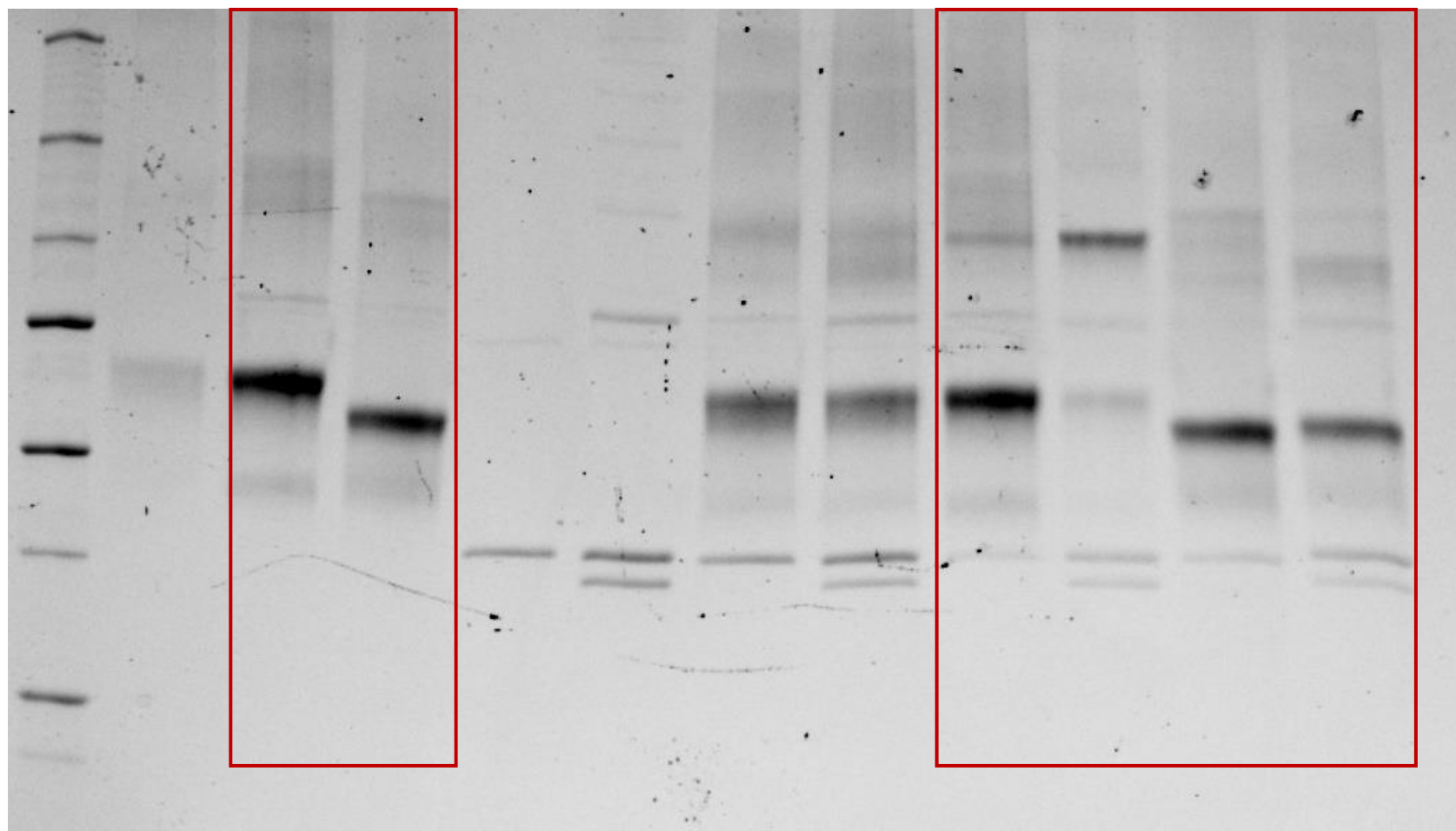

Supplement: Supplementary file 18 — Unprocessed SDS–PAGE gel image. [file 41594_2026_1800_MOESM18_ESM.pdf]
