# Supplementary material for: Structural mechanism for noncanonical GPCR signaling in the Hedgehog pathway
Source: Nat Struct Mol Biol. 2026 Apr 30;33(5):795–809. doi: 10.1038/s41594-026-01800-z (PMC13186710; doi:10.1038/s41594-026-01800-z)

Source Data ED fig. 5b top

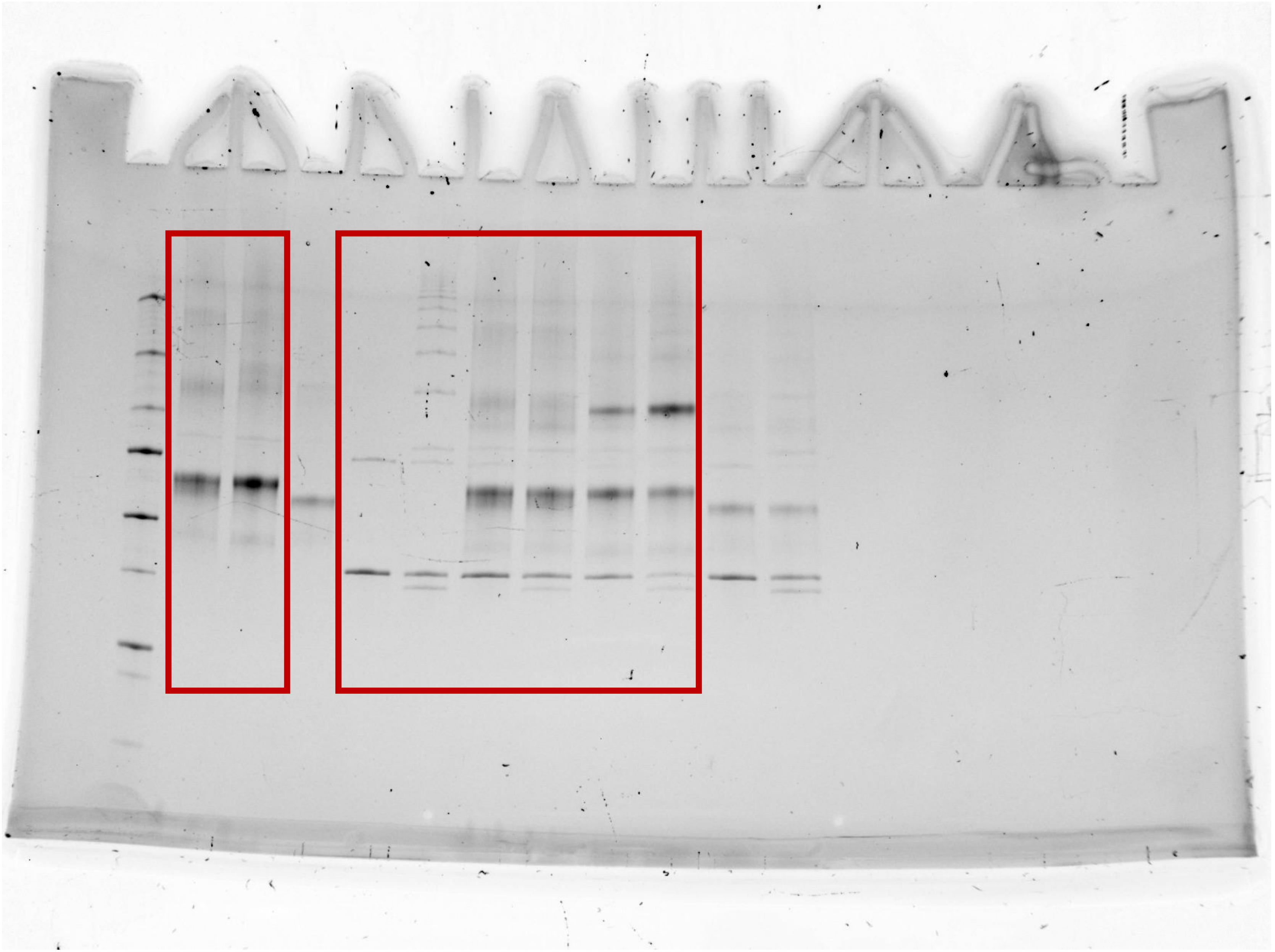

Source Data ED fig. 5b bottom

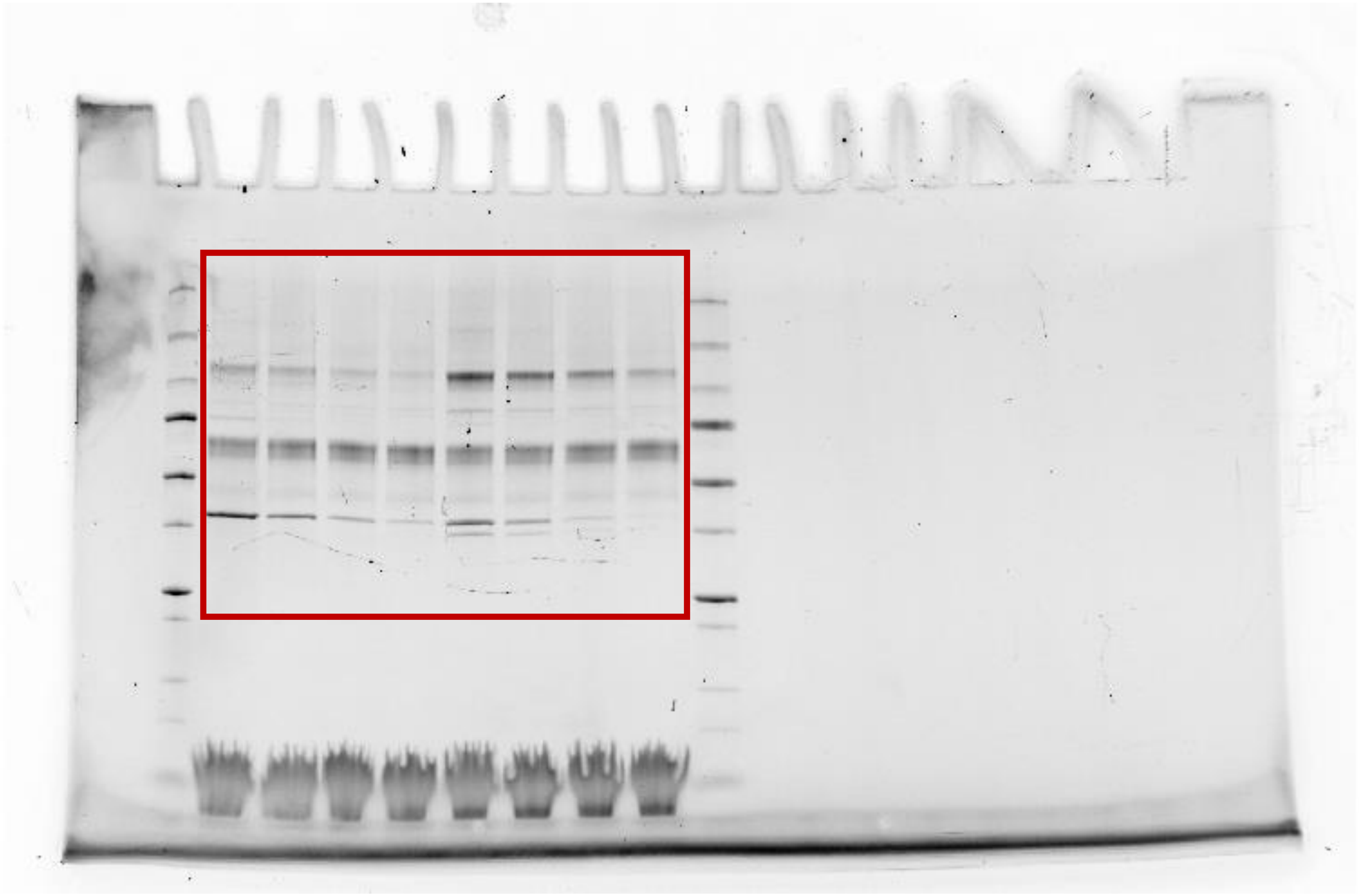

Source Data ED fig. 5e total SMO (myc) blot

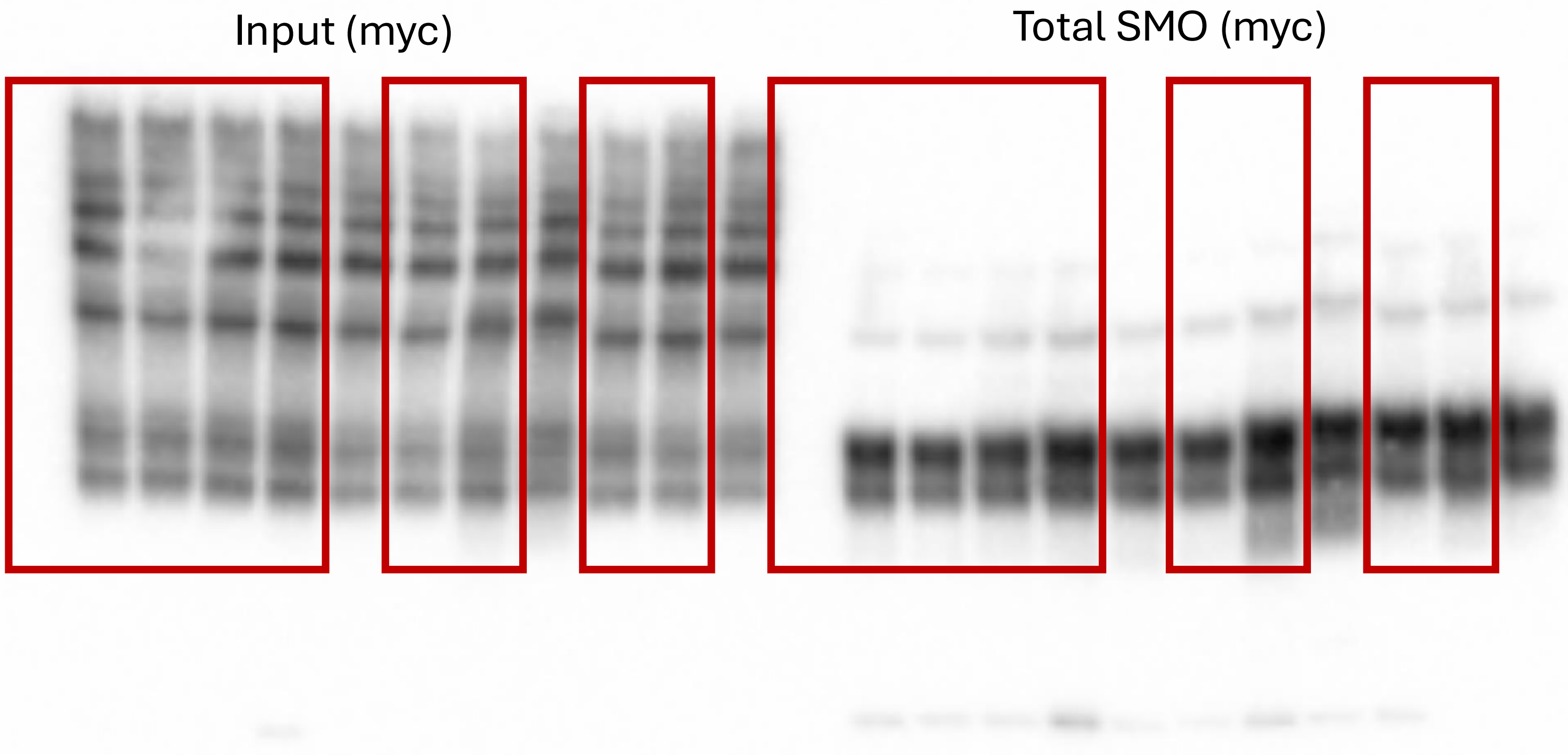

Source Data ED fig. 5e pSMO #7 blot

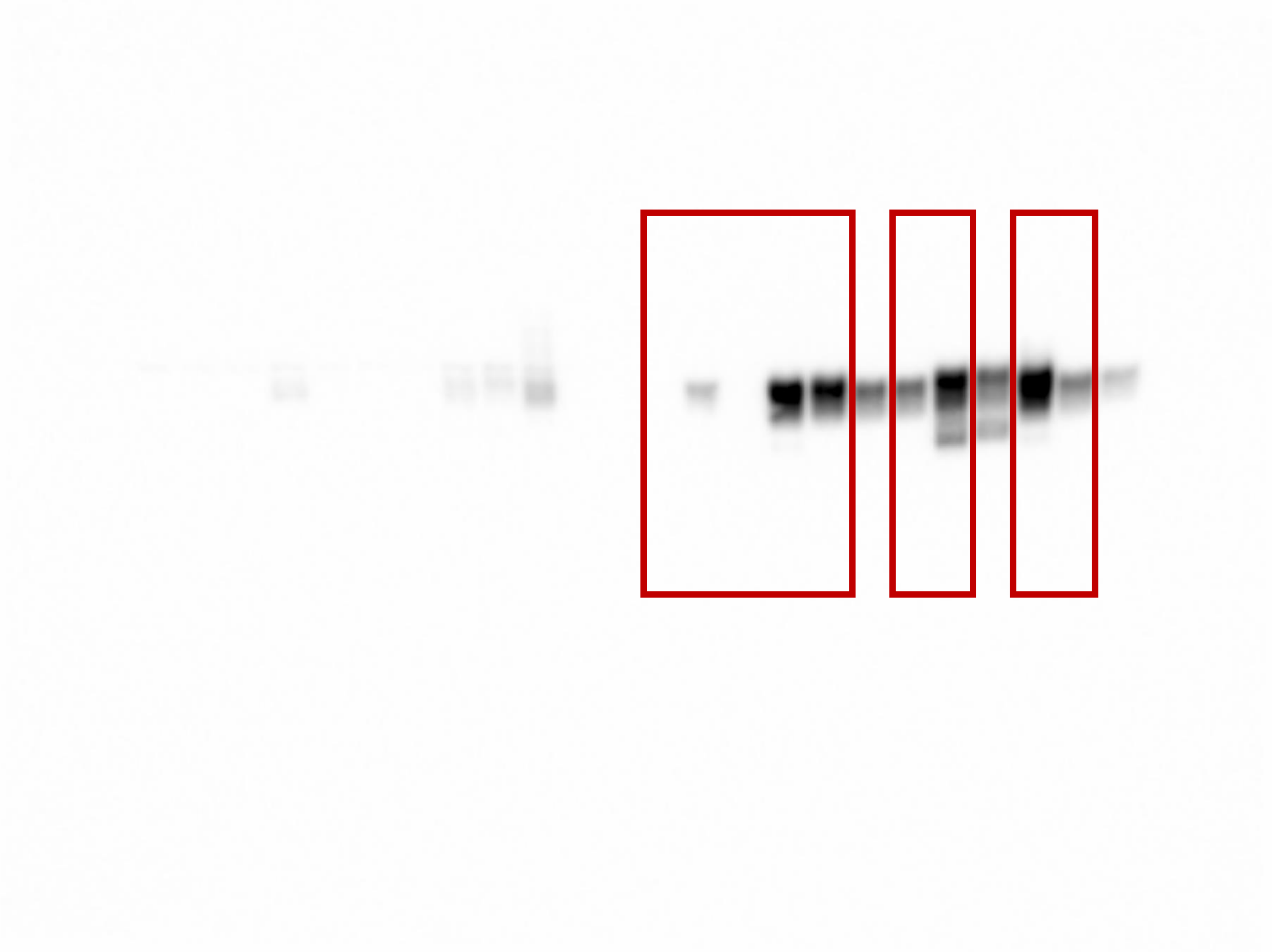

Source Data ED fig. 5e total SMO (myc) stain free blot with molecular weight markers

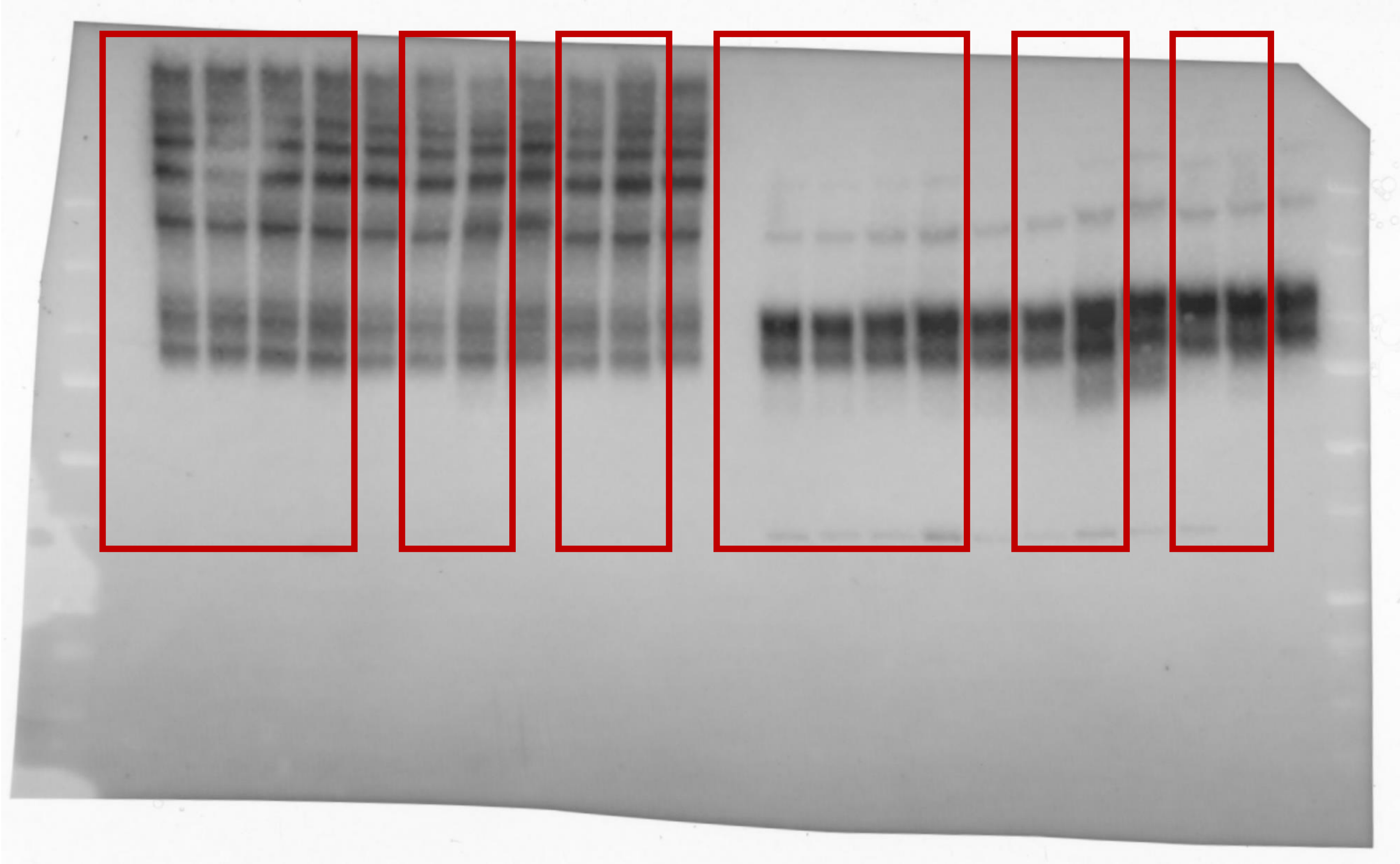

Supplement: Supplementary file 15 — Unprocessed SDS–PAGE gel and western blot images. [file 41594_2026_1800_MOESM15_ESM.pdf]
